# A synoptic review of the ant genera (Hymenoptera, Formicidae) of the Philippines

**DOI:** 10.3897/zookeys.200.2447

**Published:** 2012-06-05

**Authors:** David M. General, Gary D. Alpert

**Affiliations:** 1 College of Sciences, Palawan State University, Puerto Princesa City, Palawan Island, Philippines; 2 Museum of Comparative Zoology, Harvard University, Cambridge, Massachusetts, 02138, USA

**Keywords:** Formicidae, Philippines, ant genera, keys, new records, species list

## Abstract

An overview of the history of myrmecology in the Philippine archipelago is presented. Keys are provided to the 11 ant subfamilies and the 92 ant genera known from the Philippines. Eleven ant genera (12%), including 3 undescribed genera, are recorded for the first time from the Philippines. The biology and ecology of the 92 genera, illustrated by full-face and profile photo-images, of Philippine ants are summarized in the form of brief generic accounts. A bibliography of significant taxonomic and behavioral papers on Philippine ants and a checklist of valid species and subspecies and their island distributions are provided.

## Introduction

The study of ants can be difficult, particularly in the tropics. Tropical ant faunas are only partly explored and information concerning them is widely scattered throughout the scientific literature. Taxonomic references are frequently hard to access, badly outdated, and difficult for the non-specialist to use. The systematics of several major genera (e.g., *Camponotus*, *Crematogaster*, and *Pachycondyla*) is still chaotic and unrecognized synonyms and undescribed species abound. Taxonomic progress is relatively slow because most type material of described material is deposited in Europe, North America, or Japan, and museums are understandably hesitant to send fragile types to workers in the developing world (Naskrecki 2004).

Introductory accounts of important tropical ant faunas are badly needed to provide an entry point for biologists interested in studying ants. The purpose of this paper is to provide such an introduction to the ant fauna of the Philippines.

Simplified keys to subfamilies and to genera are presented. Most character states in the keys are discernible with a 40X stereomicroscope. Technical terms and subtle character states are minimized. Full-face and profile color photo-images are included to illustrate the different genera found in the Philippines. A glossary is also included to help the student understand the precise technical terms used in the keys. And the most up-to-date systematic arrangement of genera is used (Bolton et al. 2006, Bolton 2011). We hope that this paper will stimulate local interest in the study of Philippine ants.

### A short history of Philippine myrmecology

The study of Philippine ant fauna began in the second half of the 1800’s, during the last century of the Spanish colonial period. Small numbers of Philippine ants were collected by European travelers who then sold or gave their collections to the great myrmecologists of that era, notably Auguste Forel and Carlo Emery. Emery described ants collected in Manila and Antipolo, Luzon Island by E. Simon, a Frenchman (Emery 1893c). Frederick Smith described a few species from the Philippines without specifying the provenance of the specimens (F. Smith 1858). These researchers described some new species, but made no effort to characterize the Philippine ant fauna as a whole.

During the early American colonial era, Richard C. MacGregor, a US-trained Australian ornithologist, Charles S. Banks and other biologists working for the Bureau of Science, Manila (Van Steenis-Kruseman 2006), traveled the archipelago and gave or sold their insect collections to Americans like Dr. W.H. Ashmead of the United States National Museum (USNM) and the great Dr. William Morton Wheeler of Harvard University. Ashmead described new species collected by Dr. P.L. Stangl of the U.S. Army, Prof. L.E. Griffin of Missouri Valley University, Dr. E.B. Copeland of the Government Laboratories in Manila, and Dr. M.H. Smith of the US Fish Commission (Ashmead 1904a, 1904b). Dr. Francis X. Williams, who studied under W.M. Wheeler, collected ants while he was looking for insect predators of agricultural pests, as a researcher for the USDA. He took advantage of the expertise of Dr. Charles F. Baker, a professor at the University of the Philippines College of Agriculture at Los Baños, Laguna. Baker collected insects prodigiously, amassing hundreds of Schmitt boxes, which were bequeathed to USNM (Evans 1985).

The first intensive ant collecting was done by Dr. James W. Chapman, Wheeler’s colleague at Harvard, who arrived in the 1910’s to be a missionary and teacher at Silliman University in Dumaguete, Negros Oriental Province, Negros Island. Chapman concentrated on studying the ants of the Cuernos de Negros Mountains, but also collected in northern Luzon and Mindanao. During the Second World War, he continued to collect ants even as Japanese soldiers were hunting him down. Fortunately, he survived capture and incarceration, and his collection, which he had hidden, was largely intact (Chapman and Chapman 1947). Most of these specimens, still in their original jars, are deposited in the Museum of Comparative Zoology (MCZ) at Harvard University. Chapman later published a list of the ants of Asia (Chapman and Capco 1951).

Interestingly, Jesuit priests were important early collectors of Hymenoptera, including ants. Ashmead studied the specimens collected by Fr. W.A. Stanton, S.J. and Fr. Robert E. Brown, S.J. (Ashmead 1904a, 1904b). Fr. Brown collected the holotype queen of the controversial genus *Pseudaphomomyrmex*, recently synonymized under the genus *Tapinoma* (Ashmead 1905, LaPolla and Longino 2006, Fisher and Bolton 2007). Fr. B.B. Lowery S.J. also collected ants in the Philippines in the 1960s (Rigato 1994, Ward 2001, Shattuck 2011). His collections have been studied by researchers all over the world.

During the Commonwealth period and after the Second World War, some Filipinos also collected ants. This fact can be gleaned from the locality labels on specimens in certain museums. Specimens collected by Domingo Empeso, H.M. Torrevillas, A. Reyes, and M. Ramos are in the collections of the MCZ and the Bernice P. Bishop Museum in Hawaii. In 1966, Dr. Clare Baltazar published a monograph on Philippine Hymenoptera, which included 235 entries for ant species (Baltazar 1966).

### Current status of Philippine myrmecology

Since the efforts of the late Dr. James W. Chapman in the 1920s to 1940s, there has been relatively little intensive collecting and the studies of ants in ecological research have been few (Calilung 2000, Caceres-Plopenio, unpubl. M.S. thesis). A recent transect study (Alpert and General, in prep.) surveyed the ants of a 27-year-old narra private reforestation project on the slopes of Mt. Isarog in the Bicol region of Luzon Island. New generic records and new species are recorded. A subsequent transect study by Joanaviva Caceres-Plopenio in a nearby area added more species distribution records and possibly new species (Caceres-Plopenio, unpubl. M.S. thesis; K. Eguchi, pers. comm.). More recently, Perry Buenavente conducted ant surveys on several mountains in Luzon and Mindanao during expeditions of the National Museum of the Philippines, accumulating many new collections (P. Buenavente, pers. comm.).

Inventories for undergraduate and graduate theses remain an important source of specimens, especially in unexplored study sites. Fortunately, the collection methods are now more standardized (Agosti et al. 2000), allowing the comparison of datasets from different localities. Government permits are required for any collecting of specimens on Philippine soil and export of the same. And the Philippine government is the perpetual owner of all specimens, including holotype specimens.

The study of Philippine ant diversity is still in its infancy. A cursory examination of the species list (see Appendix) shows a strong sampling bias in the known distribution of species. Luzon Island has at least 265 species while Mindanao Island, only slightly smaller in area, has but 99 recorded species. Neither island is close to being well-collected. The large islands in the central Philippines, with the exception of Negros, namely Leyte, Mindoro, Panay, and Samar, are very poorly represented in the list. Any attempt at understanding the biogeography of Philippine ants is therefore premature.

Perhaps the best documented ant fauna in the Philippines is that of the Cuernos de Negros Mountains near Dumaguete City, Negros Island. This area was the favorite hunting ground of Chapman, who collected there for about two decades (Chapman and Chapman 1947). Nevertheless, with only 123 species known from Negros Island, more intensive and systematic collection methods will certainly turn up new species and new distributional records there.

Much has changed since the publication of Baltazar’s monograph. Many of the genera have been revised, many species have been added to the list, and a number of names have been synonymized. This paper presents an updated list containing 474 valid species and subspecies names for ants in the Philippines.

### Recent taxonomic contributions relevant to the study of Philippine ants

There has been some recent taxonomic progress. Recent monographs relevant to the Philippine ant fauna include: *Acanthomyrmex* (Moffett 1986, Agosti 1992), *Acropyga* (LaPolla 2004), *Anillomyrma* (Eguchi et al. 2010), *Anochetus* (Zettel 2012) *Calyptomyrmex* (Shattuck 2011), *Cardiocondyla* (Seifert 2003), *Carebara* (Fernandez 2004), *Euprenolepis* (LaPolla 2009), *Forelophilus* (Zettel and Zimmerman 2007), *Gnamptogenys* of the Old World (Lattke 2004), *Iridomyrmex* (Heterick and Shattuck 2011), *Leptomyrmex* (Lucky and Ward 2010), *Liomyrmex* (Rigato and Bolton 2001), *Lordomyrma* (Taylor 2012), *Lophomyrmex* (Rigato 1994), *Mayriella* (Shattuck and Barnett 2007), *Meranoplus* (Schödl 1998), *Monomorium* (Heterick 2001), *Myrmoteras* (Moffett 1985, Agosti 1992, Zettel and Sorger 2011), *Mystrium* (Bihn and Verhaagh 2007), *Odontomachus* (Sorger and Zettel 2011), *Paratopula* (Bolton 1988), *Prionopelta* (Shattuck 2008a), *Pristomyrmex* (Wang 2003, Zettel 2006), *Proceratium* (Baroni Urbani and de Andrade 2003), *Probolomyrmex* (Eguchi et al. 2006), *Pyramica* (now also *Strumigenys* sensu Baroni Urbani and de Andrade 2007)(Bolton 2000), *Recurvidris* (Bolton 1992, Zettel 2008), *Rhoptromyrmex* (Bolton 1986), *Strumigenys* (Bolton 2000), *Technomyrmex* (Bolton 2007), *Tetraponera* (Ward 2001) and *Vombisidris* (Zettel and Sorger 2010a).

### Why study ants in the Philippines?

The high diversity of ants in the Philippines makes inventory studies interesting, with little of the monotony of encountering the same species over and over again. Ant surveys can detect the presence of invasive species and provide insights into the biogeography of the islands, a baseline for pre-operation inventories of mining sites and a measure of post-mining or post-logging remediation. There is much to be done and discovered, and many opportunities await the Filipino myrmecologist.

There are many interesting study sites in the Philippines. Particularly interesting are: old growth forests, old mangrove areas, small islands (preferably uninhabited), wooded ravines, reforested areas, and even microhabitats such as soil and forest canopies. Each island or province has its own opportunities. The ant faunas of the large central islands remain unexplored. Surprisingly, new species have been found in even the most disturbed localities, such as university campuses (DMG, unpub. notes), possibly relicts of a more diverse ant assemblage (S. Cover, pers. comm.).

### Geographical and bio-climatic features of the Philippines

The Philippines is so ecologically diverse that it is very difficult to characterize the country in a single paragraph. While the climate is generally tropical and maritime, there are zones which are distinctly different. The mean temperature ranges from 25.5° C in January to 28.3° C in May, however, high-elevation locations (>1,000 masl) are sub-temperate. Baguio City, in northern Luzon, has a mean annual temperature of only 18.3˚C. Some parts, such as southern Cotabato Province, Min- danao Island, are relatively dry with an average annual rainfall of only 978 mm (PAGASA 2010). In contrast, 5713 mm of rainfall was measured at 1650 masl on Mt. Isarog in 114 days from November 1993 to May 1994 (Heaney et al. 1999). There are also wet tropical zones, mainly in the eastern part of the country, and dry tropical zones that are in the rain shadow (west) of mountain ranges. Camarines Sur, a province in the Bicol Peninsula of Luzon Island, for instance, has three distinct climate patterns, depending on elevation and position relative to Mt. Isarog, the dominant feature of the landscape. There is also a typhoon corridor, mainly in the eastern part of the archipelago, which is visited by an average of 19 typhoons every year (PAGASA 2010).

The Philippines is composed of over 7,100 islands, most of which are uninhabited. There are islands, such as Sibuyan Island in the central Philippines, which have never been connected to larger islands, even during the last Ice Age (Heaney et al. 1999). Single mountains, usually volcanoes, also exist as island habitats within the broader lowlands. This complex blend of climatic diversity and opportunity for geographic isolation has likely led to high levels of endemism, a phenomenon that deserves much further exploration. For example, two adjacent islands, Biliran and Samar, each have their own species of *Meranoplus*, a ground-dwelling myrmicine ant (Schödl 1998; DMG, unpub. notes). The Palawan island group is unique in that many of its plants and animals, including some ants, are found nowhere else in the Philippines, or in the world. Palawan Island is the type locality of the enigmatic leptanilline genus *Noonilla*.

### Threats to Philippine ant diversity

The Philippines is considered one of the hottest of the biodiversity hotspots because of the severe human pressure on its highly endemic native flora and fauna. Time is running out for the the remaining primary forests. Despite decades of regulation and reforestation, forests continue to dwindle. For instance, all the privately reforested narra trees (Fabaceae: *Pterocarpus indicus* Willd.) of the study site of our 2003-4 transects (Alpert and General in prep.) had already been poached by 2009, wiping out 27 years of stewardship of the area. Even the stumps were removed (DMG, pers. obs.). Forest destruction seems to go on unabated, exacerbated by new large-scale mining projects that seem to target the mineral resources under primary forests.

Aside from habitat destruction, invasive ant species also impact the native ant assemblages, although the extent and severity of this influence is not known. In both urban and agricultural areas are found the worldwide invasive species, e.g., *Solenopsis geminata* (Fabricius 1804), *Tapinoma melanocephalum* (Fabricius 1793), and *Paratrechina longicornis* (Latreille 1802). Other invasive species have not been found, but this may be due to a lack of attention.

### Some forest habitats in the Philippines

We include some images of intact and heavily damaged forests to illustrate the potentials and problems for ant research in the Philippines. The Philippine ant fauna is very poorly explored and records are few and spotty at best. A researcher can essentially choose a mountain among several hundred mountains, get the necessary permits and be the first one to study the ants on that mountain. However, that mountain may also be very disturbed or degraded, leaving only the tramp species that abound in disturbed habitats. In addition to environmental damage, there is also an active communist insurgency and other serious security threats to researchers in the field. Other areas may harbor malaria mosquitoes and other serious health threats. Hence, local collaboration is quite necessary to minimize the risks of field work in the Philippines.

## Genus accounts of Philippine ant genera

In this contribution, the acronym “PH” is used to represent the archipelago of the Republic of the Philippines, in compliance with the International Organization for Standardization 3166-1 country codes (ISO 2010). The archipelago should also be referred to as “the Philippines” not “the Philippine Islands”. The expression “Philippine Islands” and the acronym “PI” are anachronisms and should no longer be used since they refer to the islands before the country gained independence in 1946.

The first electronic checklist of Philippine ants is available online at Discoverlife (Alpert et al. 2006). Another online resource is the Philippine page on AntWeb, hosted by the California Academy of Sciences (Alpert and General 2008).

**Figure 1.**
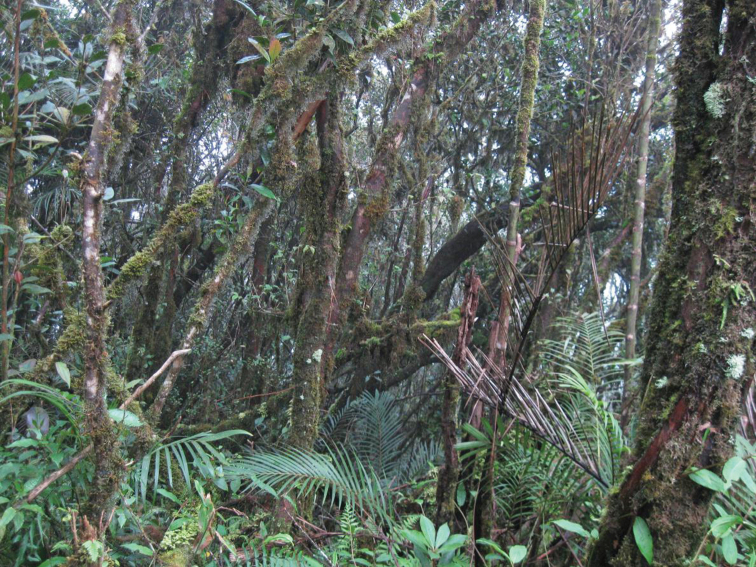
Montane forest, Mt. Palali, Sierra Madre Mountain Range, Luzon Island. Image used with permission from Arvin C. Diesmos.

**Figure 2.**
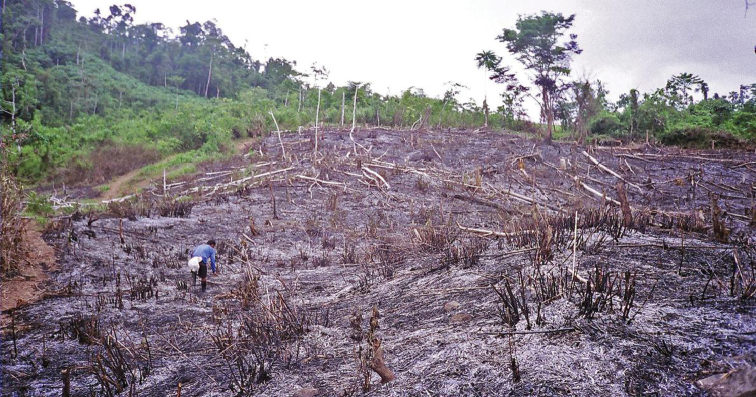
Slash and burn agriculture, Sierra Madre Mountain Range, Luzon Island. Image used with permission from Arvin C. Diesmos.

**Figure 3.**
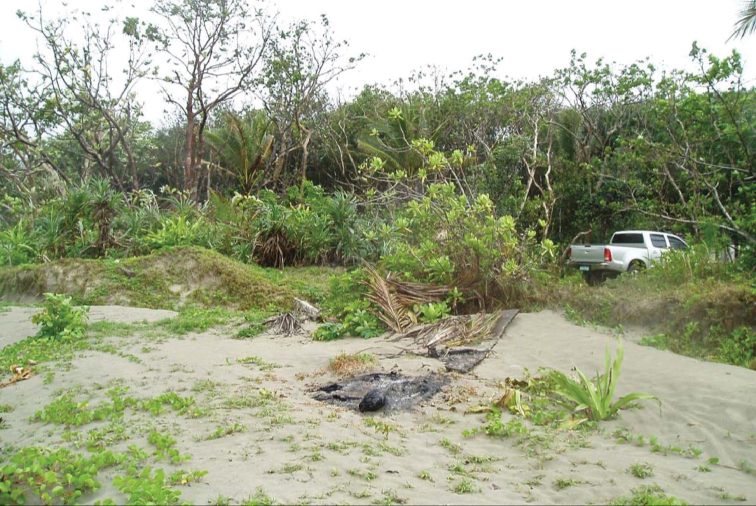
Beach forest, Aurora Province, Luzon Island. Image used with permission from Arvin C. Diesmos.

**Figure 4.**
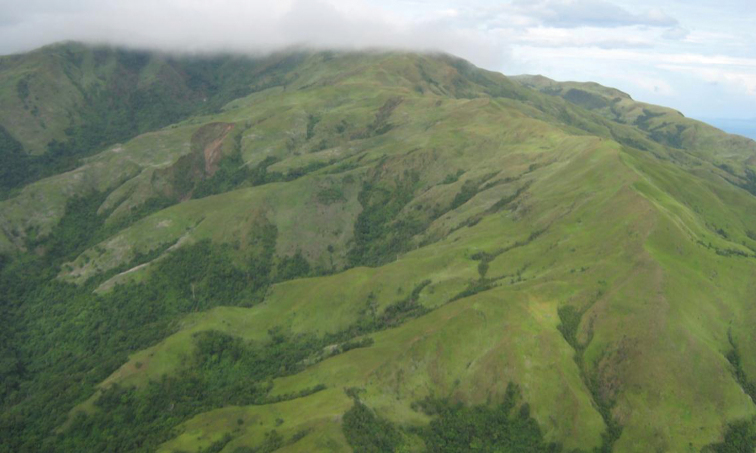
Deforested mountain, northwestern Mindoro Island.

**Figure 5.**
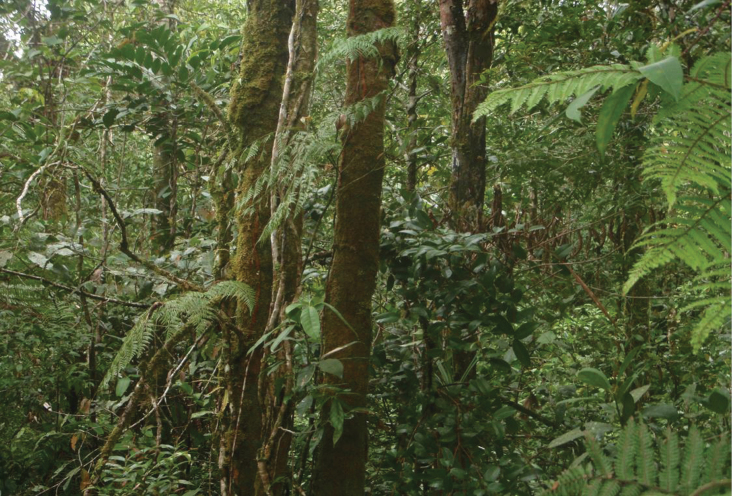
Mossy forest, Mt. Balatukan, northeastern Mindanao Island. Image used with permission from Perry Buenavente.

**Figure 6.**
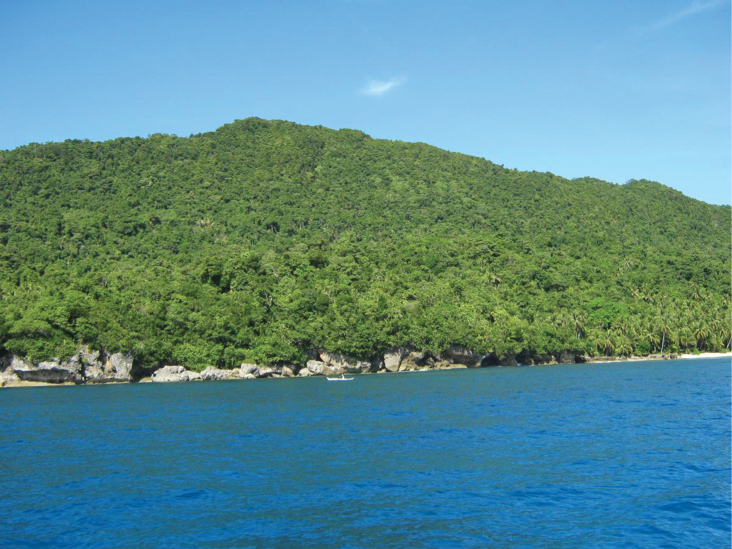
Lowland dipterocarp forest, Bucas Grande Island, Surigao del Norte Province.

**Figure 7.**
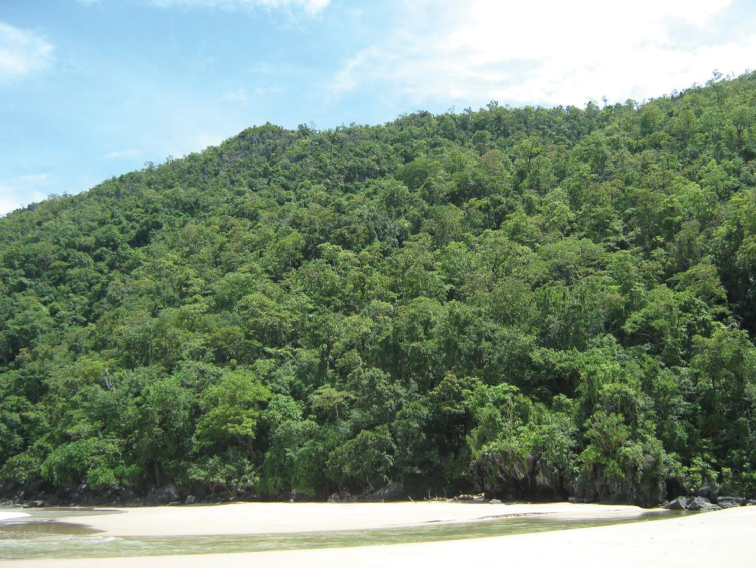
Lowland dipterocarp forest, at the mouth of the Puerto Princesa Subterranean River, St. Paul Bay, Puerto Princesa City, Palawan.

**Figure 8.**
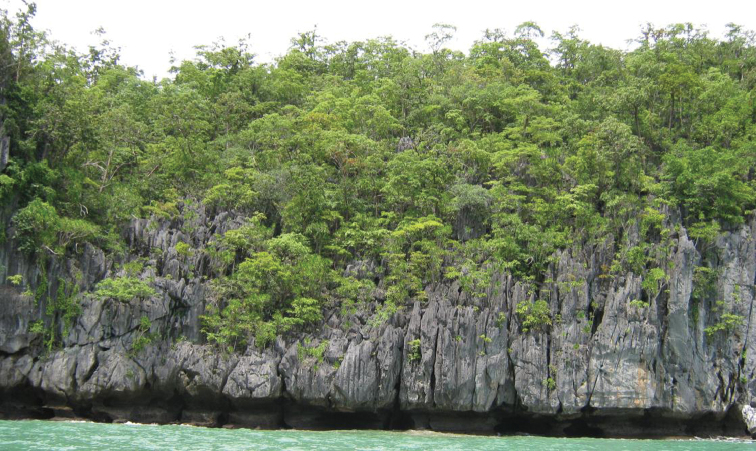
Limestone karst forest, St. Paul Bay, Puerto Princesa City, Palawan.

Because these are genus accounts, plural terms, e.g., “these ants” or “they” refer to species belonging to the genus. Singular terms, e.g., “this species” or “it” refers to a particular species in the genus. The genus account typically introduces Philippine material referable to the particular genus, then gives a brief description of the morphology or behavior of the ants belonging to the genus and finally suggests collecting techniques. Some behavioral aspects may be inferred from published studies of non-Philippine congeners.

### Genus *Acanthomyrmex* Emery, 1893a

Myrmicinae: 17 spp, 1 known from PH.

Figs. 9 A, B

The single known valid Philippine species, *Acanthomyrmex mindanao* Moffett, 1986, was described from specimens collected under bark in Mt. McKinley, Davao Region, southern Mindanao Island. Other specimens were collected in northern Mindanao Island: Momungan, Olangon, and Iligan City, Lanao del Sur Province and Gingoog City, Misamis Oriental Province. This species is also known from Sarawak, Malaysia. These ants may be collected by sifting leaf litter, baiting with small seeds, and looking under bark. Keys to species: Moffett (1986) (World, revision), Agosti (1992) (World, revision), Terayama et al. 1998.

**Figure 9.**
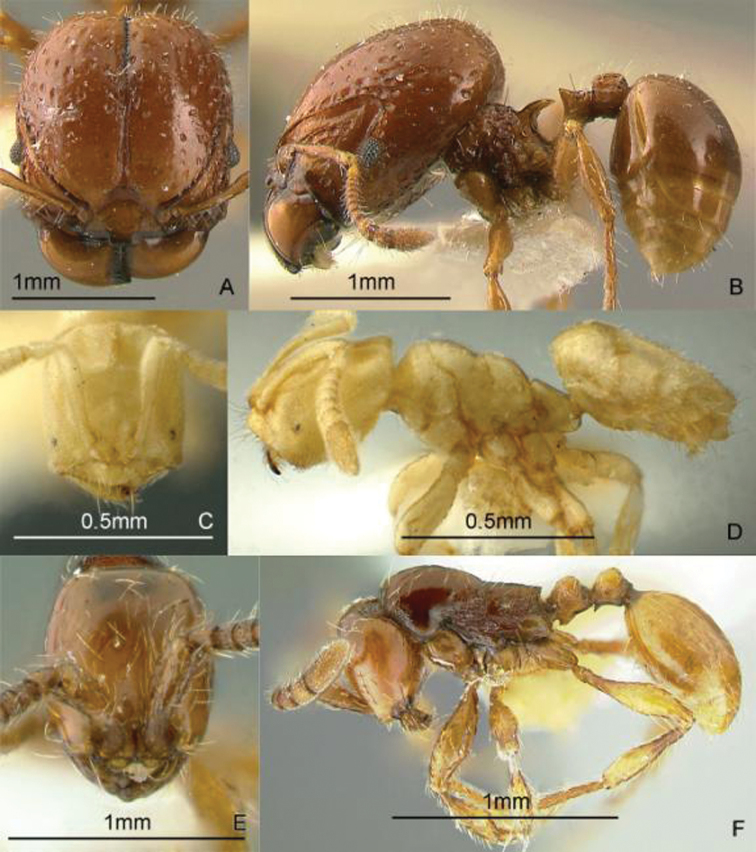
Full-face and profile images of Philippine ant genera. *Acanthomyrmex mindanao*, major worker **A, B** *Acropyga pallida*, minor worker **C, D** *Aenictus ceylonicus* (Mayr, 1866) **E, F**.

### Genus *Acropyga* Roger, 1862

Formicinae: 40 spp., 3 known from PH.

Figs. 9 C, D

**(New record).** This genus is pantropical. These tiny, yellow, hypogaeic ants are known to tend subterranean coccids. One species known from the Philippines, *Acropyga pallida* (Donisthorpe, 1938), was originally described from New Guinea. These ants may be collected by sifting leaf litter and soil cores up to about 10 cm deep. Key to species: LaPolla (2004).

### Genus *Aenictus* Shuckard, 1840

Aenictinae: 149 spp., 15 known from PH.

Figs. 9 E, F

This genus is widespread throughout the archipelago. Large colonies forage for insect prey in the leaf litter. These are true army ants, making bivouacs in tree hollows and other protected cavities. These ants may be collected by searching for conspicuous columns of emigrating or foraging ants, and carefully inspecting cavities in trees and logs. For raiding behavior, see Schneirla and Reyes (1966). Keys to species: Wheeler (1930c) (Philippines), Wilson (1964) (Indo-Australian, revision), Terayama and Yamane (1989) (Indonesia, Sumatra), Shattuck (2008b) (Australian, review of genus), Zettel and Sorger (2010b) (new Philippine species described).

### Genus *Anillomyrma* Emery, 1913b

Myrmicinae: 2 spp., 1 known from PH.

Figs. 10 C, D

Specimens of *Anillomyrma decamera* (Emery, 1901) were recently collected by Joanaviva Caceres-Plopenio in a transect study on Mt. Isarog, Luzon Island (Caceres, unpubl. M.S. thesis; Eguchi et al. 2010). These pale, tiny, thin-skinned subterranean ants seem to prefer sandy soils. These ants may be collected by sifting soil cores, preferably by Berlese or Winkler extraction, or baiting underground in sandy loam areas. Key to species: Eguchi et al. 2010 (World, revision).

**Figure 10.**
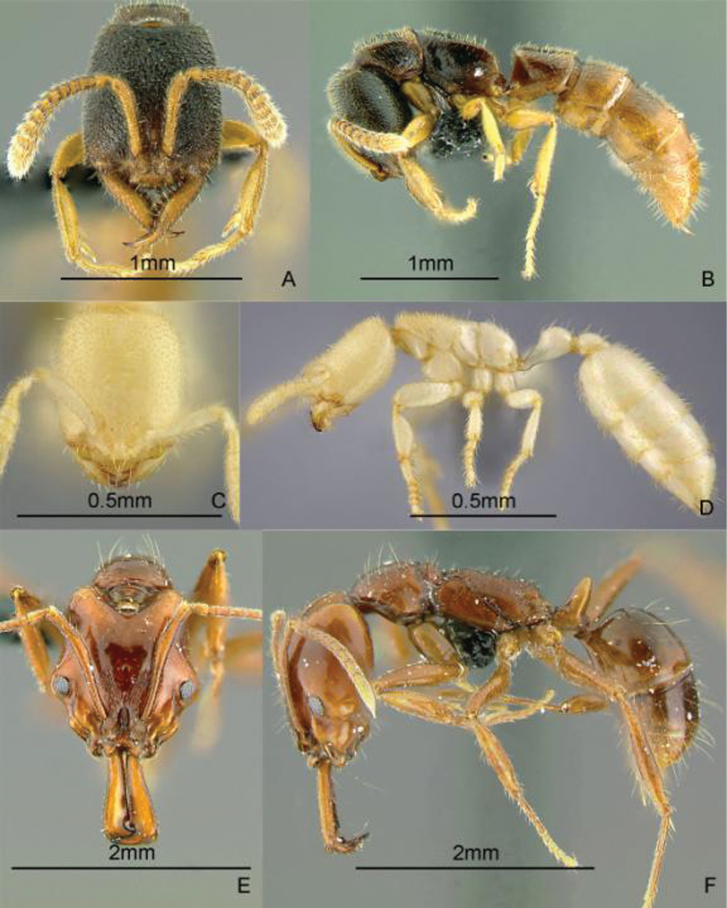
Full-face and profile images of Philippine ant genera. *Stigmatomma luzonicum* Wheeler and Chapman, 1925 (formerly *Amblyopone luzonica*)**A, B** *Anillomyrma decamera* (images reproduced with permission of Dr. Katsuyuki Eguchi) **C, D** *Anochetus isolatus* Mann, 1919 **E, F**.

### Genus *Anochetus* Mayr, 1861

Ponerinae: 87 spp., 13 known from PH.

Figs. 10 E, F

These small, fast-moving trap-jawed ants are general predators and usually found foraging singly on the ground or in the leaf litter. These ants are similar to *Odontomachus*, except that they are smaller and have blunt petiolar nodes. Key to species: Brown (1978) (World), Zettel (2012) (Philippine species).

### Genus *Anomalomyrma* Taylor, 1990a

Leptanillinae: 2 spp., 1 known from PH.

Figs. 11 A, B

The worker caste of this enigmatic genus was recently discovered and described, including *Anomalomyrma helenae* Borowiec, Schulz, Alpert & Baňař, 2011 from northern Palawan Island (Borowiec et al. 2011). These tiny subterranean ants have a petiole and postpetiole whose tergites and sternites are fused to form rigid tubes, a unique morphology among ants. These ants may be collected by leaf litter sifting and underground baiting near water sources.

**Figure 11.**
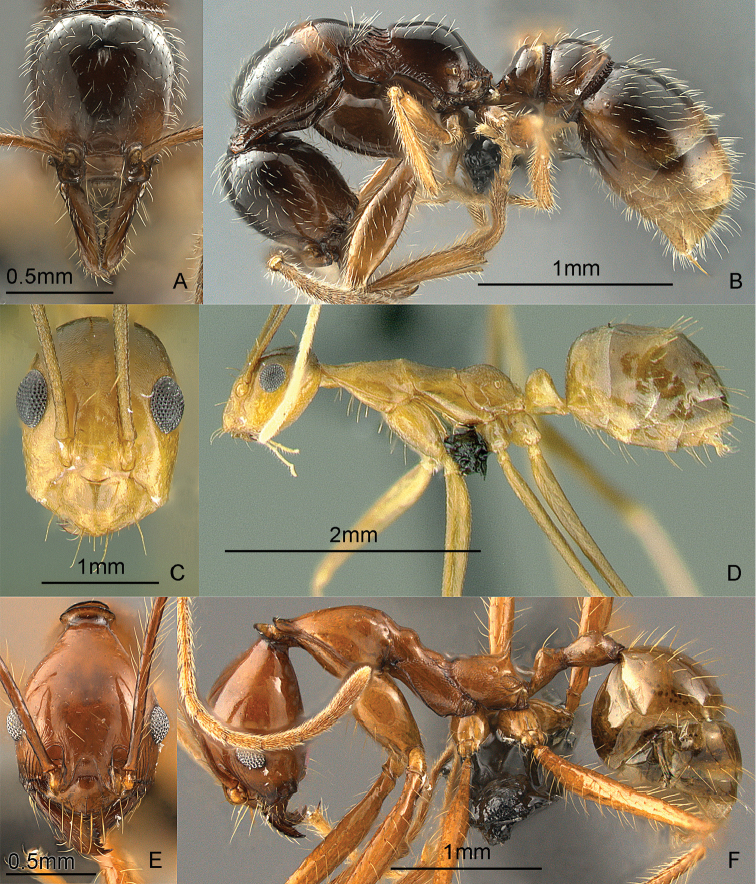
Full-face and profile images of Philippine ant genera. *Anomalomyrma helenae* **A, B** *Anoplolepis gracilipes* **C, D** *Aphaenogaster* species PH02 **E, F**.

### Genus *Anoplolepis* Santschi, 1914

Formicinae: 14 spp., 1 known from PH.

Figs. 11 C, D

The species found in the Philippines, *Anoplolepis gracilipes* (F. Smith, 1857), is a pantropical invasive ant, with 11-segmented antennae with extremely long antennal scapes and a slender, constricted mesosoma in dorsal view. It is locally dominant around its nest. This species may nest in the ground or in a tree hollow. The myrmecophilous associations observed by the present authors indicate that this ant may be native to the Philippines. Upon opening a nest in a tree hollow, we found that muscoid flies immediately hovered over the scampering workers and the brood. In a coconut farm in Candelaria, Quezon Province, DMG also observed and collected an immature reduviid bug mimicking the erratic movement, color, and size of this ant. It may be collected by carefully inspecting tree hollows and rotten logs, baiting with tuna and searching for trails of foraging workers on the ground and along branches.

### Genus *Aphaenogaster* Mayr, 1853

Myrmicinae: 227 spp., 2 known from PH.

Figs. 11 E, F

**(New record).** There are historical specimens of two undescribed species, collected by Chapman and his field crew, of this genus in the ant collection of the Museum of Comparative Zoology, Harvard University. There are also new collections from Min-

danao Island (Figs. 11 E,F) (DMG, unpubl. notes). These ants typically nest in rotten logs and the soil under the logs. These ants may be collected by pitfall trapping, breaking into rotten logs and searching for foragers on the ground.

### Genus *Basiceros* Schulz, 1906

Myrmicinae: 60 spp., 3 known from PH.

Figs. 12 A, B

Ants of this genus may be confused with some *Strumigenys* species, but they have more than 6 antennal segments. These small, cryptic, slow-moving ants have clavate hairs all over the body. They forage in the leaf-litter. When disturbed, these ants roll up into a tight ball which makes them even harder to find. After about a minute, they stretch out and start walking slowly. There are specimens of an unidentified species from a transect study at Mt. Isarog, Bicol Region, Luzon Island (Alpert and General in prep.). Specimens were collected at 550 meters. These ants may be collected by sifting leaf litter over a white sheet, waiting a while, and carefully inspecting seedlike objects that begin to walk slowly. Keys to species (as *Eurhopalothrix*): Brown and Kempf (1960) (World); Taylor (1968, 1980, 1990b) (Indo-Australian, Australasian), Baroni Urbani and de Andrade 2007 (synonymy of *Eurhopalothrix* under *Basiceros*).

**Figure 12.**
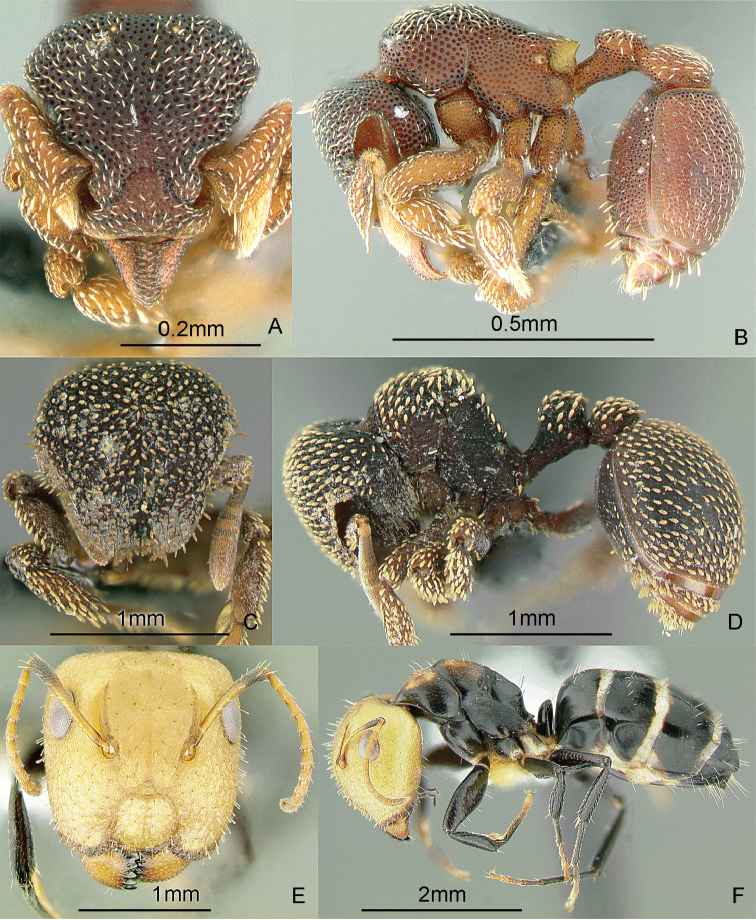
Full-face and profile images of Philippine ant genera. *Basiceros philippinum* (Brown and Kempf, 1960) **A, B** *Calyptomyrmex beccarii* Emery, 1887 **C, D** *Camponotus albocinctus* (Ashmead, 1905), major worker **E, F**.

### Genus *Calyptomyrmex* Emery, 1887b

Myrmicinae: 25 spp., 2 known from PH.

Figs. 12 C, D

These small, cryptic, slow-moving ants bear a hard, thick integument with numerous evenly-spaced clavate hairs. They curl up into a ball when disturbed and are found in the leaf litter or on the ground. These ants may be collected by sifting leaf litter on a white sheet, waiting a while and carefully inspecting seed-like objects. After about a minute, these seed-like objects may stretch out and start to walk slowly. Keys: Baroni Urbani (1975) (Indian subcontinent); Bolton (1981) (Afrotropical); Shattuck (2011) (Southeast Asian revision).

### Genus *Camponotus* Mayr, 1861

Formicinae: 1,584 spp., 30 known from PH.

Figs. 12 E, F

*Camponotus* is an extremely large genus in dire need of taxonomic revision. This is a widespread genus in the Philippines. Twenty-eight species are presently known from the Philippines, but this is probably only a fraction of the total. This genus is unusual among formicines in that the usual conspicuous ring of hairs around the acidipore is absent. This genus can be recognized by the placement of the antennal insertions, which are always set back (not adjacent to) from the posterior clypeal border. *Camponotus* are often medium to large ants; dimorphic or polymorphic workers that forage along trails from their nest in wood. Some species are nocturnal. Mimicry also occurs in this genus. There is a single undetermined minor worker in the Philippine collection of the Bernice P. Bishop Museum that looks very similar to ants of the genus *Dolichoderus* (GDA, unpub. notes). These ants may be collected by breaking into cavities in live wood or dead branches and searching for foragers on the ground, foliage, tree trunks and branches or in the canopy.

### Genus *Cardiocondyla* Emery, 1869a

Myrmicinae: 69 spp., 5 known from PH.

Figs. 13 A, B

This genus is known to have both normal winged and worker-like wingless males which fight and exhibit interesting courtship behavior (Heinze et al. 1993, Yamauchi et al. 2005, Mercier et al. 2007). These tiny, ground- and rock-dwelling ants bear the characteristic swollen postpetiole which is wider than long, and much larger than the petiole. Several species are pantropical tramps. There are 5 species known from the Philippines. One species, *Cardiocondyla sima* Wheeler, 1935b, is apparently widespread, reported in Lanao Province, Mindanao Island and in Mt. Isarog, Bicol Region, Luzon Island (Alpert and General in prep.). These ants may be collected by leaf litter sifting, pitfall trapping and baiting with cookie crumbs on exposed rocks in creeks and rivers and following the forager to its nest in the rock’s crevices. Key to species: Seifert (2003) (Selected species-groups only).

**Figure 13.**
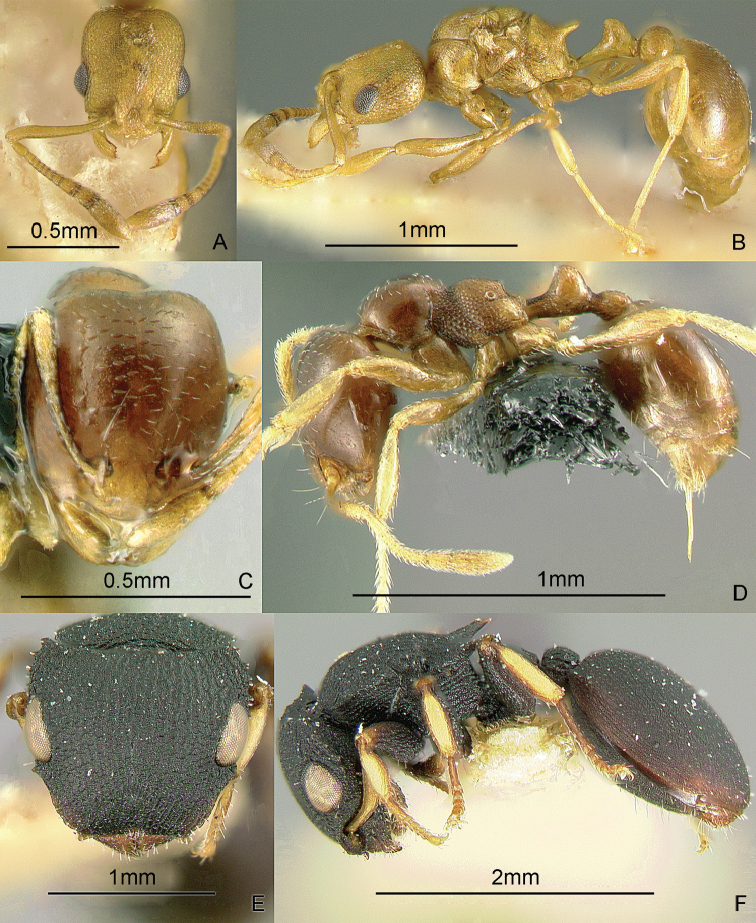
Full-face and profile images of Philippine ant genera. *Cardiocondyla sima*, queen **A, B** *Carebara alperti* Fernandez, 2010 **C, D** *Cataulacus chapmani* Bolton, 1974 **E, F**.

### Genus *Carebara* Westwood, 1840

Myrmicinae: 175 spp., 3 known from PH.

Figs. 13 C, D

The Philippine species of this pantropical genus are poorly known. There are specimens of two unidentified species from a transect study at Mt. Isarog, Bicol Region, Luzon Island (Alpert and General in prep.). Other species have been collected on Samar Island and in Misamis Occidental Province, Mindanao Island. These are very tiny ants with dimorphic workers. The major workers may possess a pair of tubercles or horns near the posterior margin of the head. These ants are ground-dwelling and may be collected by sifting soil and leaf litter. Key to species: Fernandez (2004) (New World only), Fernandez (2010) (description of first Philippine species).

### Genus *Cataulacus* F. Smith, 1853

Myrmicinae: 69 spp., 3 known from PH.

Figs. 13 E, F

These robust, hard-bodied ants have a wide, sculptured head and the antennal scrobe passing below the eye. They are known to be arboreal, can glide back to the tree trunk when they fall (Yanoviak et al. 2008) and nest in small hollow twigs or rotten branches of live trees. These ants may be collected by searching dead branches still attached to the tree and beating low vegetation over a white sheet. Key to species: Bolton (1974) (World revision).

### Genus *Centromyrmex* Mayr, 1866b

Ponerinae: 15 spp., 1 known from PH.

Figs. 14 A, B

*Centromyrmex feae* (Emery, 1889) is the only species recorded from the Philippines at present. These small, cryptic ants have a pronotum that is somewhat flattened dorsally, mandibles that sharply curve downward and backward (in side view), and middle tibiae with strong peg-like setae. The workers are weakly polymorphic, with slight differences among nestmates. They are hypogaeic. Some evidence indicates that they are associated with termites. These ants may be collected by sifting leaf litter and soil cores at least 10 cm deep.

**Figure 14.**
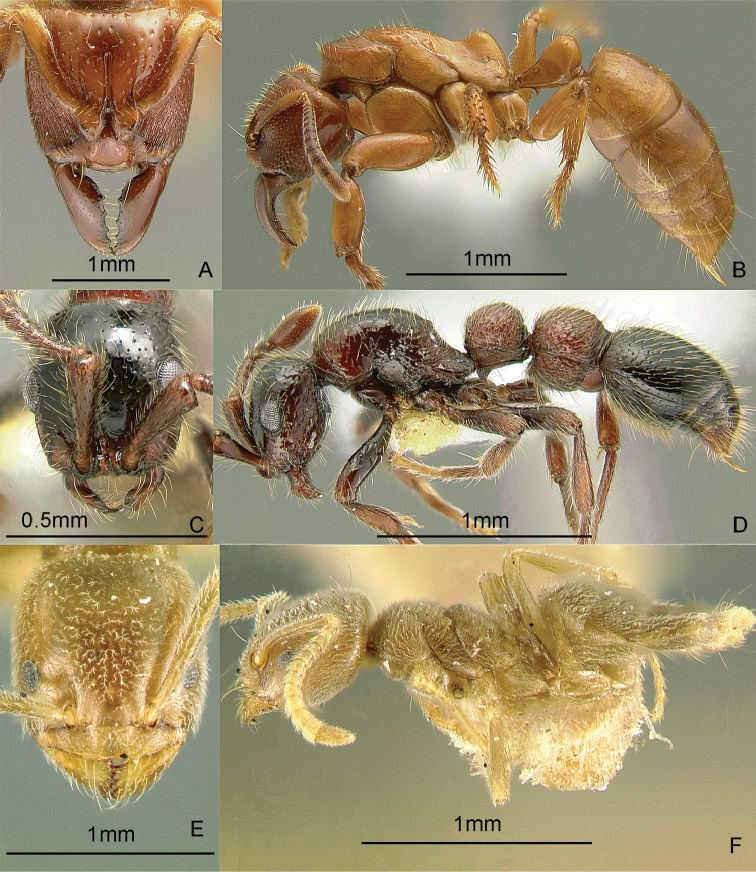
Full-face and profile images of Philippine ant genera. *Centromyrmex feae* **A, B** *Cerapachys rufithorax* Wheeler and Chapman, 1925 **C, D** *Chronoxenus* species PH01 **E, F**.

### Genus *Cerapachys* F. Smith, 1857

Cerapachyinae: 144 spp., 11 known from PH.

Figs. 14 C, D

These hard-bodied ants have a large, globular petiole and a larger, barrel-shaped postpetiole. They are predators on other ants and some species conduct group raids on other ant nests. During a raid, they steal the larvae and pupae which they sting. These stung prey remain alive for a long time, providing fresh food for the colony. In Mt. Isarog, Bicol Region, Luzon Island, they have been found nesting in twigs, with a larder of pupae of another ant genus, *Crematogaster* (Alpert and General in prep.). These ants may be collected by breaking open twigs on the ground and in the leaf litter, sifting leaf litter, and searching for columns of raiding workers. Key to species: Brown (1975) (World).

### Genus *Chronoxenus* Santschi, 1919

Dolichoderinae: 46 spp., 2 from PH.

Figs. 14 E, F

Dubovikov (2005) recently revived this genus from synonymy to receive all Oriental species of *Bothriomyrmex* Emery, 1869b. A specimen of an unidentified species, collected from Palawan Island in 1925, is in the ant collection of the Museum of Comparative Zoology, Harvard University. There are specimens of an undetermined species, collected in Laguna Province, Luzon Island, in the Insect Collection of the International Rice Research Institute. An excellent nest series with all the castes of possibly another species was recently collected by Perry Buenavente from Mt. Diwata, Agusan del Sur Province, Mindanao Island. These small ants, superficially similar to *Tapinoma*, have a petiole with an erect scale; with short, indistinct palps and a propodeum with a short dorsal face and a long declivity. These ants may be collected by sifting leaf litter including the humus layer. Emery (1925) (out of date); Shattuck (1992c) (generic revision of subfamily).

### Genus *Crematogaster* Lund, 1831

Myrmicinae: 780 spp., 16 known from PH.

Figs. 15 A, B

These small ants have a characteristically heart-shaped gaster that can flex over the mesosoma. They are often associated with coccids and aphids, sometimes building carton or soil shelters over these sap-sucking insects. They may be hypogaeic, epigaeic or arboreal. Similar to *Cataulacus*, they can glide back to the tree trunk when they fall, earning the nickname “acrobat ants” (Yanoviak et al. 2008). These ants may be collected by breaking open carton or soil that is at the apices of young branches, sifting leaf litter and soil, inspecting plant roots, beating low vegetation over a white sheet, and baiting with peanut butter on tree trunks or cookie crumbs on the ground. Key: Hosoishi and Ogata (2009a) (subgenus *Physocrema*); Hosoishi and Ogata (2009b) (checklist of Asian species).

**Figure 15.**
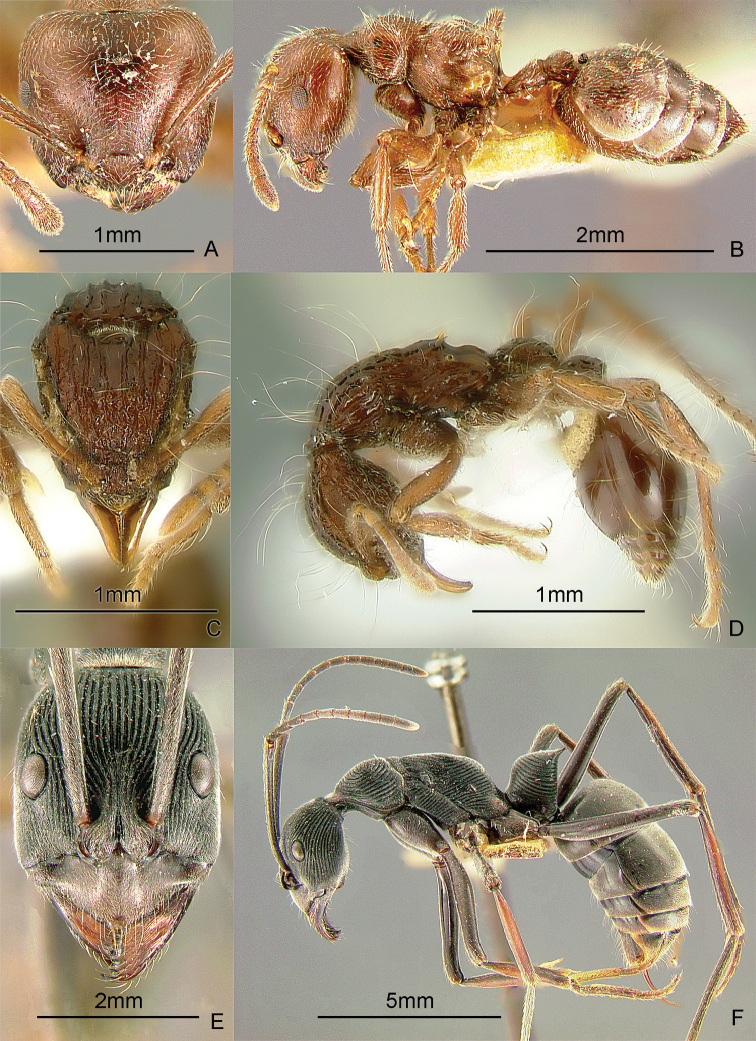
Full-face and profile images of Philippine ant genera. *Crematogaster difformis* F. Smith, 1857 **A, B** *Dacetinops cirrosus* Taylor, 1985 **C, D** *Diacamma panayense* Wheeler and Chapman, 1925 **E, F**.

### Genus *Dacetinops* Brown & Wilson, 1957

Myrmicinae: 7 spp., 1 known from PH.

Figs. 15 C, D

**(New record).** Dr. Herbert Zettel (Natural History Museum, Vienna, Austria) has a single specimen, tentatively identified as *Dacetinops cirrosus*, collected near Calbiga-a River, Mt. Pangasugan, Baybay, Leyte Island. These ants bear spongiform tissue on the ventral surfaces of the petiole, postpetiole, and the first gastral segment. They nest in rotten wood. These ants may be collected by sifting leaf litter and breaking into rotten twigs and wood on the forest floor. Key to species: Brown and Wilson (1957), Taylor (1985) (Papuasian).

### Genus *Diacamma* Mayr, 1862

Ponerinae: 44 spp., 5 known from PH.

Figs. 15 E, F

This genus is long overdue for revision. These ants are easily distinguished from other ponerines by the distinctive costate sculpturing that covers the head, mesosoma, and the petiole, which has 2 dorsal spines. The late Dr. W.L. Brown, Jr. believed that male characters may hold the key to producing stable species boundaries. These large black ants are ground-dwelling or arboreal and hunt singly for prey. Ants of this genus have a unique social structure in which the queen caste is absent and all workers have the potential to mate and lay eggs. Peeters and Higashi (1989) (reproductive dominance behavior).

### Genus *Dilobocondyla* Santschi, 1910

Myrmicinae: 11 spp., 3 known from PH.

Figs. 16 A, B

These small hard-bodied ants have the upper corners of the head drawn into broad points and have a distinctive barrel-shaped petiole. They are known to be arboreal but may forage on the ground as well. An unidentified species is reported from a transect study at Mt. Isarog, Camarines Sur Province, Luzon Island (Alpert and General in prep.). These ants may be collected by beating low vegetation over a white sheet and inspecting dead branches still attached to the tree for nests.

**Figure 16.**
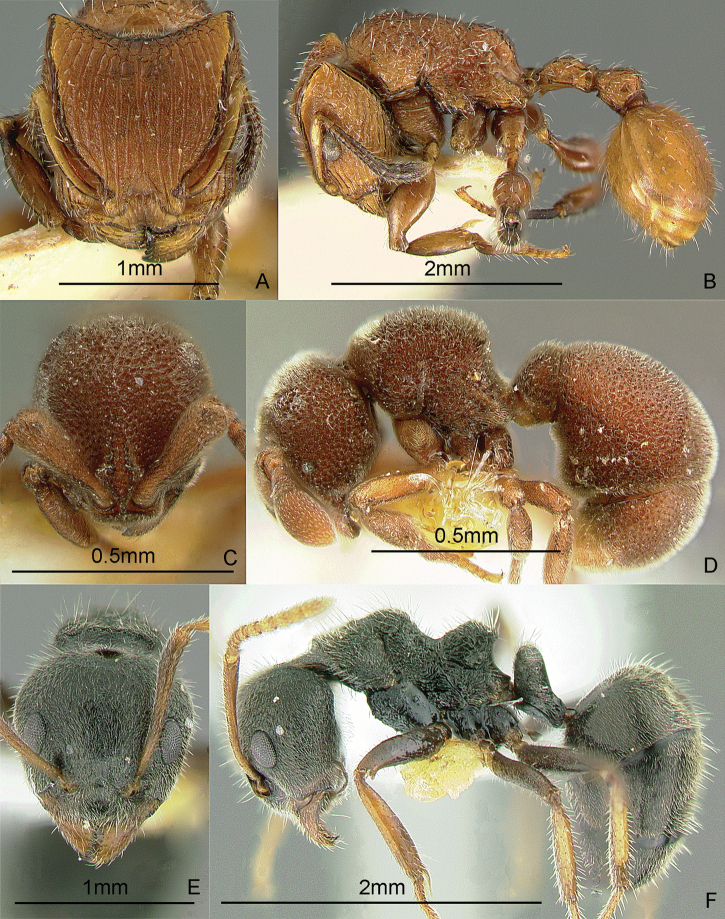
Full-face and profile images of Philippine ant genera. *Dilobocondyla chapmani* Wheeler, 1924 **A, B** *Discothyrea clavicornis* Emery, 1897a **C, D** *Dolichoderus thoracicus* (F. Smith, 1860a) **E, F**.

### Genus *Discothyrea* Roger, 1863

Proceratiinae: 32 spp., 5 known from PH.

Figs. 16 C, D

**(New record).** There are specimens of *Discothyrea bryanti* (Wheeler, 1917) and *Discothyrea clavicornis* (Shattuck, pers. comm.) from adjacent stations of a transect study at Mt. Isarog by Joanaviva Caceres-Plopenio in 2006. This is remarkable because few species of this genus are sympatric, much less on the same transect (Shattuck, pers. comm.). Another unidentified species was found from a different transect study at Mt. Isarog, Bicol Region, Luzon Island (Alpert and General in prep.). Specimens were collected at 550-700 meters above sea level. Two unidentified species were extracted from lowland forest leaf litter berlesate from Samar Island. Another unidentified species was collected from Palawan Island. These small cryptic ants have an extremely large apical antennal segment and the petiole broadly attached to the gaster. They are known to be predators of arthropod eggs. These ants may be collected by sifting leaf litter, pitfall trapping, and searching for nests under rocks. Brown (1958) (generic revision).

### Genus *Dolichoderus* Lund, 1831

Dolichoderinae: 148 spp, 3 known from PH.

Figs. 16 E, F

The most common species, *Dolichoderus thoracicus*, is the black, hard-bodied ant usually associated with the locally-popular fruit of the *lanzones* tree (Meliaceae: *Lansium domesticum* Corr.), and of the *makopa* tree (Myrtaceae: *Syzygium samarangense* (Blume) Merrill and Perry) tending the mealybugs (Hemiptera: Sternorrhyncha: Pseudococcidae) that are found in the fruit bunches (DMG, pers. obs.). This ant, which forages day and night, is also common in urban and highly disturbed areas. These ants are easy to collect, with newly-mated queens even venturing indoors at night (DMG, unpubl. notes).

### Genus *Echinopla* F. Smith, 1857

Formicinae: 26 spp., 5 known from PH.

Figs. 17 A, B

Specimens of two unidentified species were collected from low vegetation in a transect study in Mt. Isarog, Bicol Region, Luzon Island (Alpert and General in prep.). One of these unidentified species was collected by nocturnal beating of low vegetation. These hairy or fuzzy ants are unique in appearance and have hard bodies and petioles armed with teeth or denticles laterally. These ants may be collected by beating of low vegetation over a white sheet and inspecting of dead branches in the foliage and canopy.

**Figure 17.**
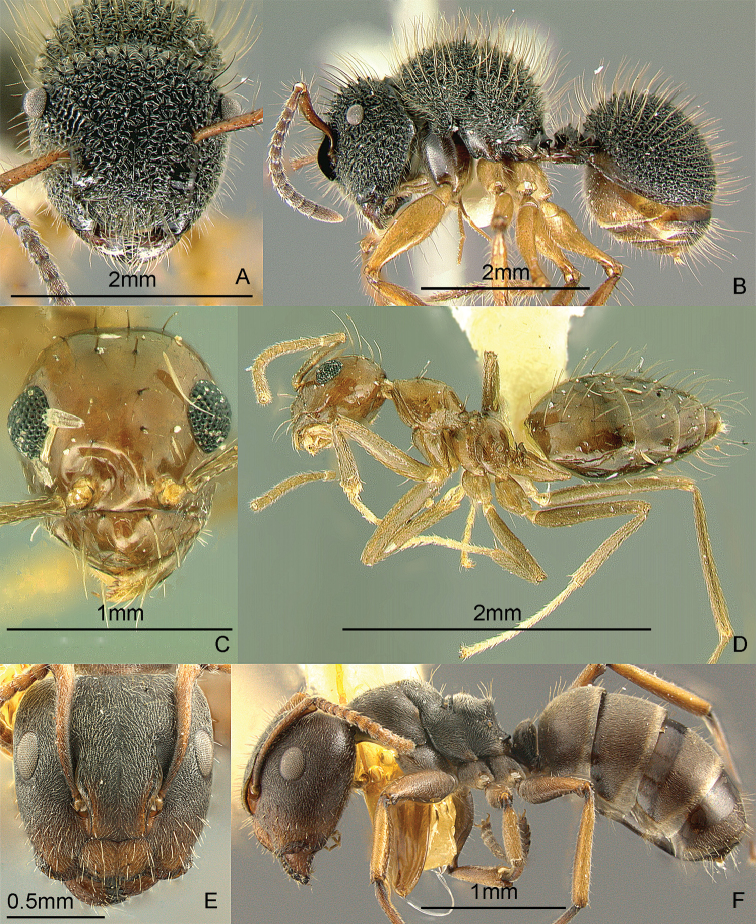
Full-face and profile images of Philippine ant genera. *Echinopla pallipes* F. Smith, 1857 **A, B** *Euprenolepis* species PH01 **C, D** *Forelophilus* species PH01 **E, F**.

### Genus *Euprenolepis* Emery, 1906

Formicinae: 6 spp., 2 known from PH.

Figs. 17 C, D

*Euprenolepis negrosensis* (Wheeler, 1930b) is known from the Philippines. It is rarely collected, perhaps because its tiny eyes indicate a subterranean life-habit. An unidentified species is depicted in Figs. 17 A, B. These ants have strongly curved mandibles such that the apical tooth is directed posteriolaterally. They forage underground, or in the leaf litter and on the ground, possibly for mushrooms, as found by Witte and Maschwitz (2008) in Malaysia. These ants may be collected by sifting leaf litter, pitfall trapping, soil core sampling and possibly by baiting with edible mushrooms. Key to species: LaPolla (2009) (World revision).

### Genus *Forelophilus* Kutter, 1931

Formicinae: 3 spp., 3 known from PH.

Figs. 17 E, F

This genus was previously known only from the type species, *Forelophilus overbecki* Kutter, 1931, described from Java. Two widespread species are now known from the Philippines, *Forelophilus stefanschoedli* Zettel and Zimmerman, 2007 known from the islands of Luzon, Leyte, and Mindanao, and *Forelophilus philippinensis* Zettel and Zimmerman, 2007 known from the islands of Luzon, Bayagnan, and Mindanao (Zettel and Zimmerman 2007). An unidentified third species is shown in Figures 17 E, F. These ants may be collected by beating low vegetation over a white sheet and hand collecting from foliage.

### Genus *Gauromyrmex* Menozzi, 1933

Myrmicinae: 2 spp., 1 known from PH.

Figs. 18 A, B

**(New record).** *Gauromyrmex acanthinus* (Karavaiev, 1935) is known from the Philippines. A single identified specimen is deposited in the MCZ Ant Collection. These small ants have a petiole with dorsolateral corners produced into acute angles or denticles. They are arboreal and may be collected by baiting with tuna or honey and by inspecting dead branches of live trees. Diagnosis: Bolton (2003).

**Figure 18.**
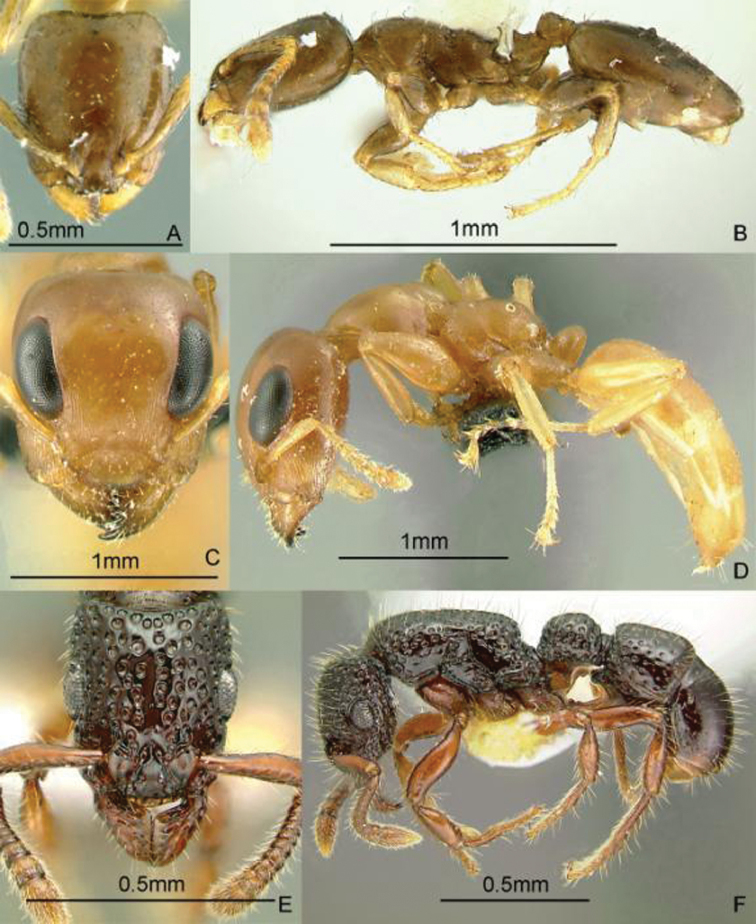
Full-face and profile images of Philippine ant genera. *Gauromyrmex acanthina* **A, B** *Gesomyrmex luzonensis*, major worker **C, D** *Gnamptogenys chapmani* Brown, 1958 **E, F**.

### Genus *Gesomyrmex* Mayr, 1868

Formicinae: 7 spp., 1 known from PH.

Figs. 18 C, D

*Gesomyrmex luzonensis* (Wheeler, 1916) is known from the Philippines. This ant is rarely collected but widespread, having been collected in a bamboo grove in Los Baños, Laguna Province, Luzon Island and in a hardwood cavity nest in Cuernos de Negros, Negros Island. All members of this genus are presumed to be nocturnal and arboreal in habitat. These ants have large bean-shaped eyes and mandibles that look like pinking scissors. They are polymorphic, with major, media, and minor workers. Wheeler (1930c) quoted J.W. Chapman’s description of the habits of *Gesomyrmex luzonensis*. Chapman found that the minors conduct most of the foraging, are attracted to ripe bananas, and are very timid. Key to species: Cole (1949); see also Wheeler (1929a, 1929b, 1930a).

### Genus *Gnamptogenys* Roger, 1863

Ectatomminae: 134 spp., 11 known from RP.

Figs. 18 E, F

These hard-bodied ants are specialized predators of other ant species. At least one species, *Gnamptogenys menadensis* (Mayr, 1887), has mostly fertile laying workers instead of a queen (Gobin et al. 1998, Gobin et al. 1999). They may be ground-dwelling or arboreal, diurnal or nocturnal. These ants may be collected by breaking open rotten wood on the ground, inspecting cavities in living trees and dead branches, sifting leaf litter, and pitfall trapping. Key to species: Lattke (2004) (SE Asia and Australasia).

### Genus *Harpegnathos* Jerdon, 1851

Ponerinae: 11 spp., 5 known from PH.

Figs. 19 A, B

These large cryptic ants bear characteristic long pliers-like mandibles and large eyes. They are usually ground-dwelling and hunt prey such as crickets in the leaf litter. A specimen of an unidentified arboreal species was collected on Mt. Isarog, Bicol Region, Luzon Island (DMG, unpubl. notes). These ants may be collected by sifting leaf litter, searching on the ground, and pitfall trapping.

**Figure 19.**
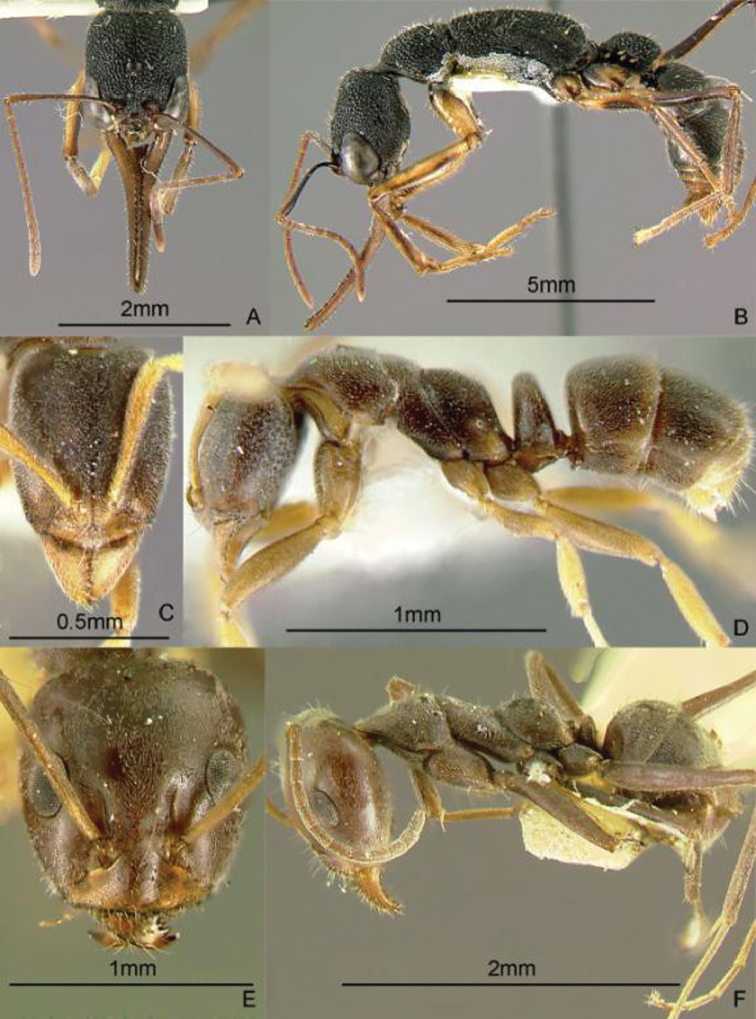
Full-face and profile images of Philippine ant genera. *Harpegnathos venator* Donisthorpe, 1937 **A, B** *Hypoponera confinis* (Roger, 1860) **C, D** *Iridomyrmex* species PH01 **E, F**.

### Genus *Hypoponera* Santschi, 1938

Ponerinae: 171 spp., 6 known from PH.

Figs. 19 C, D

The taxonomy of this large pantropical genus is currently chaotic. These small, cryptic ants are very similar to *Pachycondyla*, but lack a second simple spur on the hind tibia. They have a simple ventral process of the petiole, without a fenestra or posterior angles as in *Ponera*. These ants make small colonies in soil, rotten wood, and leaf litter. These ants may be collected by sifting leaf litter, beating low vegetation, pitfall trapping, and searching in rotten wood. There is no modern key to species, however, Barry Bolton is currently revising this genus (B. Bolton, pers. comm.).

### Genus *Iridomyrmex* Mayr, 1862

Dolichoderinae: 82 spp., 2 known from PH.

Figs. 19 E, F

These fast-moving ants may be confused with *Dolichoderus* but have a thinner cuticle and bear the characteristic wavy anterior clypeal margin. A specimen of *Iridomyrmex anceps* (Roger, 1863) was recently collected from Marinduque Island by Joanaviva Caceres-Plopenio (P. S. Ward, pers. comm). There is a specimen of one unidentified species, collected from Baguio, Benguet Province, Luzon Island, in the MCZ ant collection. Other unidentified specimens have been collected from the provinces of Pangasinan, Isabela, and Camarines Sur on Luzon Island, and the islands of Camiguin and Semirara. They are ground-dwelling and usually form large colonies. These ants may be collected by sifting leaf litter, pitfall trapping, and baiting on the ground. Key to species: Heterick and Shattuck (2011) (World revision).

### Genus *Lepisiota* Santschi, 1926

Formicinae: 131 spp., 2 known from PH.

Figs. 20 A, B

These small, yellow or brown arboreal ants are characterized with a distinct angulate propodeum. They have been collected in the islands of Luzon, Negros, and Palawan, but rarely. These ants may be collected by beating low vegetation over a white sheet and inspecting cavities in living wood and dead branches in the canopy.

**Figure 20.**
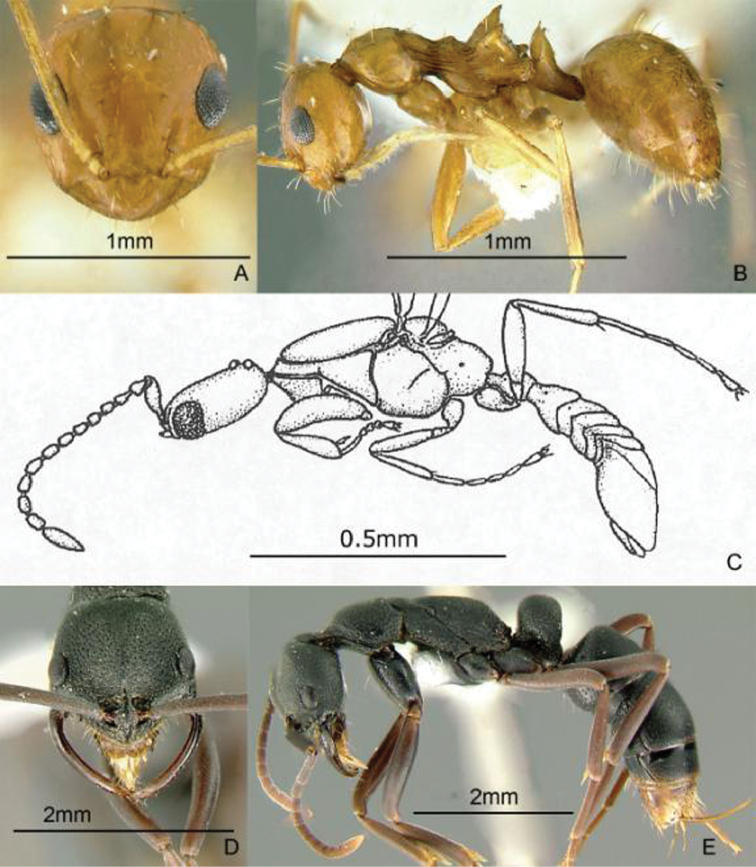
Full-face and profile images of Philippine ant genera. *Lepisiota chapmani* Wheeler, 1935 **A,  B** *Leptanilla astylina* Petersen, 1968 (line drawing reprinted from Entomologiske Meddelelser, with permission) (C). *Leptogenys maxillosa* (F. Smith, 1858) **D, E**.

### Genus *Leptanilla* Emery, 1870

Leptanillinae: 43 spp., 1 known from PH.

Fig. 20 C

Only *Leptanilla astylina* is known from the Philippines (Palawan). It was described from the male alate. These tiny, blind, hypogaeic ants have a slim, elongated body, fully exposed antennal socket and a swollen postpetiole. They are extremely difficult to collect, perhaps because they live deep in the soil. Soil cores 10 cm deep during transect studies at Mt. Isarog failed to turn up any leptanilline ants (Alpert and General in prep.). These ants may be collected by sifting soil cores up to 30 cm deep and underground baiting with centipede carcasses. Key to species: Baroni Urbani (1977b) (World), Ogata et al. (1995).

### Genus *Leptogenys* Roger, 1861

Ponerinae: 248 spp., 11 known from PH.

Figs. 20 D, E

These long-bodied and slender ants bear the characteristic clypeus extending forward to form a rounded triangle. They also exhibit army ant-like behavior with large raiding columns and are known to prey on termites. They usually have worker-like, or ergatoid, queens and nest in rotten wood, under rocks, and in the ground. These ants may be collected by sifting leaf litter, flipping over rocks, pitfall trapping, and searching for raiding columns. Key to species: Wilson (1958) (Melanesian and New Caledonia).

### Genus *Leptomyrmex* Mayr, 1862

Dolichoderinae: 41 spp., 1 known from PH.

Figs. 21 A, B

This genus occurs mostly in Australia, New Guinea, New Caledonia, and Aru Island, Indonesia. *Leptomyrmex* is unique in the subfamily in having an elongated head, mesosoma, and legs. In addition, most of the queens are wingless. A single specimen of *Leptomyrmex fragilis* (F. Smith, 1859) has been found in the Bernice P. Bishop Museum collection. The specimen was collected by L.W. Quate in 1959 in San Francisco, Agusan del Sur Province, Mindanao Island. Lucky and Ward (2010) consider this specimen a doubtful record, however it is validated by the many legitimate records of other insects in the Quate collection. These ants may be collected by sifting leaf litter, pitfall trapping, baiting on the ground, and searching for nests at the base of trees and in rotten wood. Key to species: Lucky and Ward (2010). Generic review: Shattuck (1992c).

**Figure 21.**
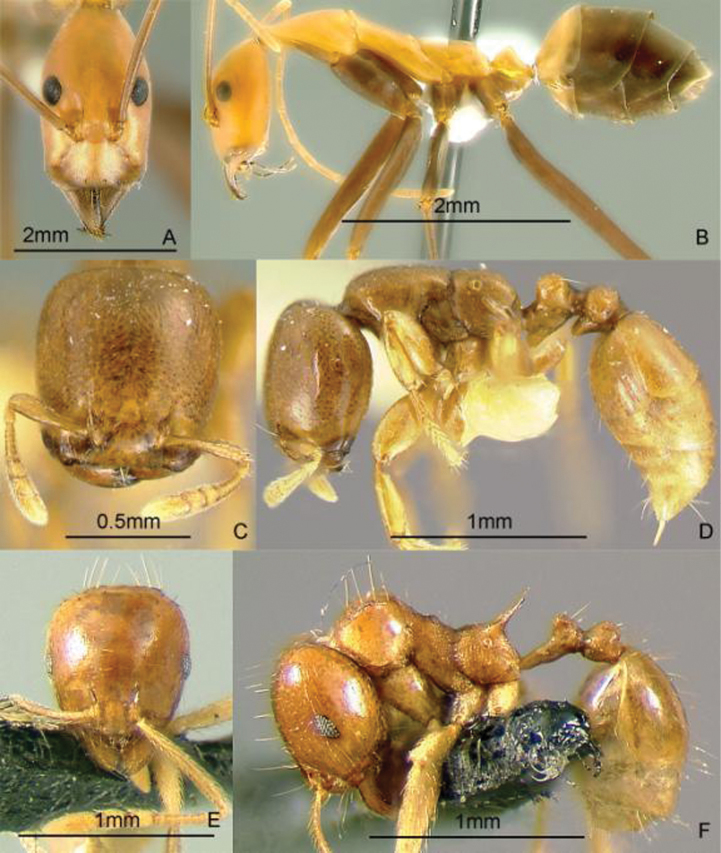
Full-face and profile images of Philippine ant genera. *Leptomyrmex fragilis* **A, B** *Liomyrmex gestroi* **C, D** *Lophomyrmex bedoti* Emery, 1893b **E, F**.

### Genus *Liomyrmex* Mayr, 1865

Myrmicinae: 1 sp., 1 known from PH.

Figs. 21 C, D

This is a monotypic tropical genus, with only one widespread species. *Liomyrmex gestroi* (Emery, 1887b) is known from throughout Southeast Asia. In the Philippines, it has been recorded from the islands of Luzon, Mindanao and Negros. This small, blind species has a smooth, almost hairless, body and bear large ventral processes on the petiole and postpetiole. It is assumed to be a kleptoparasite of mound-building termites, having been found inside live termite mounds and in forest leaf litter with termites. It may be collected by sifting leaf litter, pitfall trapping, and breaking into termite mounds to find nests. Rigato and Bolton (2001) (World revision).

### Genus *Lophomyrmex* Emery, 1892a

Myrmicinae: 12 spp., 1 known from PH.

Figs. 21 E, F

*Lophomyrmex bedoti* was collected by general collecting in Palawan. These monomorphic ants are hunter/scavengers, preying on various arthropods on the forest floor. They nest near or at the base of trees and form persistent soil-walled trails (similar to the trails of *Pheidologeton* ants), which may also run underground. These ants may be collected by baiting with sugar or protein bait and searching for nests at the base of trees and for conspicuous trails on the forest floor. Key to species: Rigato (1994) (World revision), Bharti and Kumar (2012) key to *bedoti* species-group.

### Genus *Lordomyrma* Emery, 1897a

Myrmicinae: 20 spp., 4 known from PH.

Figs. 33 A, B

Taylor (2012) recently described four new species from the Philippines:one species from Leyte Island and three sympatric species from Mt. Isarog Natural Park, Luzon Island. A specimen of an unidentified species was collected in a transect study in Bulacan Province, Luzon Island. These small monomorphic ants are variable in morphology but usually have a prominently convex pronotum. They forage in the foliage and on the ground. Little is known of their biology. These ants may be collected by beating low vegetation, sifting leaf litter, flipping rocks and breaking into rotten wood for nests. Taylor (2012) (descriptions and images of Philippine species).

### Genus *Mayriella* Forel, 1902c

Myrmicinae: 7 spp, 1 known from PH.

Figs. 22 C, D

The species known from the Philippines, *Mayriella transfuga* Baroni Urbani, 1977a, is wide-ranging in the Asian tropics and was originally described from Nepal. Ants of this genus are tiny, hard-bodied ants with a clypeus that extends as two lobes over the mandibles. The head and mesosoma are usually heavily sculptured while the gaster is smooth. They forage on the ground and nest in rotten wood and under stones. These ants may be collected by sifting leaf litter, pitfall trapping, and flipping stones and breaking into rotten wood for nests. Key to species: Shattuck and Barnett (2007) (World revision).

### Genus *Meranoplus* F. Smith, 1853

Myrmicinae: 62 spp., 2 known from PH.

Figs. 22 E, F

**Figure 22.**
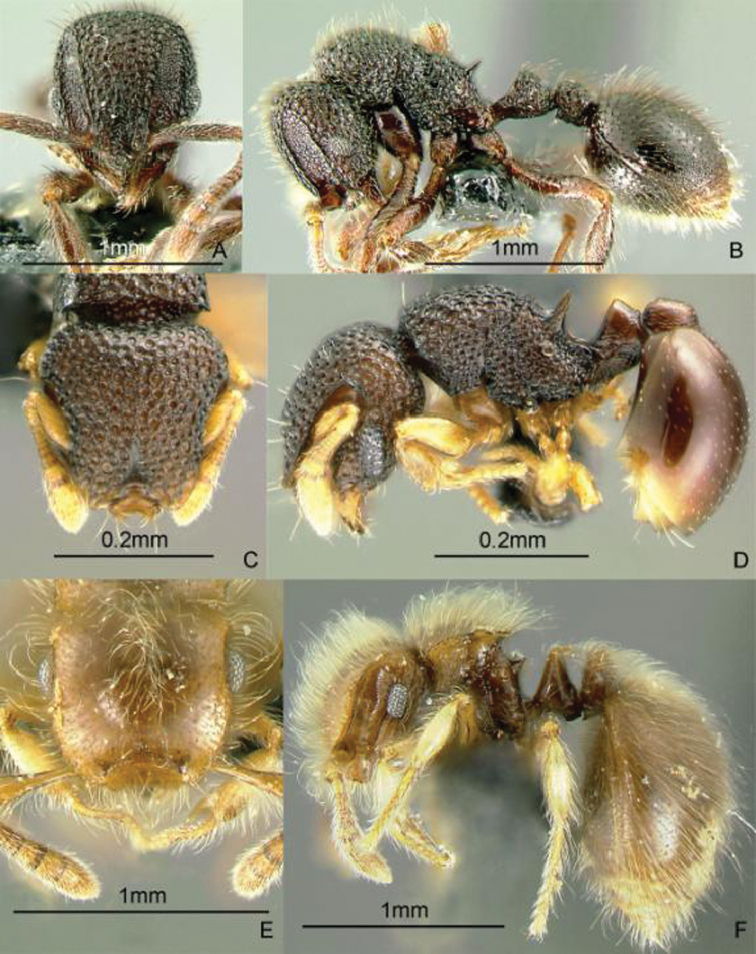
Full-face and profile images of Philippine ant genera. *Lordomyrma emarginata* Taylor 2012 **A, B** *Mayriella transfuga* **C, D** *Meranoplus* species PH01 **E, F**.

Only one species, *Meranoplus biliran* Schödl, 1998, is known from Biliran Island. There are specimens of another, possibly undescribed, species from the nearby island of Samar (Figs. 22 E,F). These slow-moving ants have the characteristic shield-like upper surface of the mesosoma, which is actually an extension of the pronotum. When disturbed, they curl up and remain motionless, similar to *Basiceros* and *Calyptomyrmex* ants. They are ground-dwelling generalist scavengers or seed predators and forage on the ground an in the foliage. They may be collected by beating low vegetation, sifting leaf litter, pitfall trapping, baiting with tuna or honey on trees, and searching for a ring of seed hulls around the nests. Key to species: Schödl (1998) (Oriental).

### Genus *Metapone* Forel, 1911d

Myrmicinae: 17 spp., 2 known from PH.

Figs. 23 A, B

The two Philippine species are known only from winged reproductives. An unidentified species is present in the Bernice P. Bishop Museum ant collection. These hard-bodied cryptic ants are commonly mistaken for ponerine ants because of their large, broadly attached postpetiole. They have deep antennal scrobes and the clypeus projects forward as a square lobe. They are often found feeding on hardwood termites in dead logs and are very rarely collected. These ants may be collected by breaking into the heartwood of hardwood logs that are suspended off the ground. Key to species: Wheeler (1919c) (World), Alpert (2007) (Madagascar).

**Figure 23.**
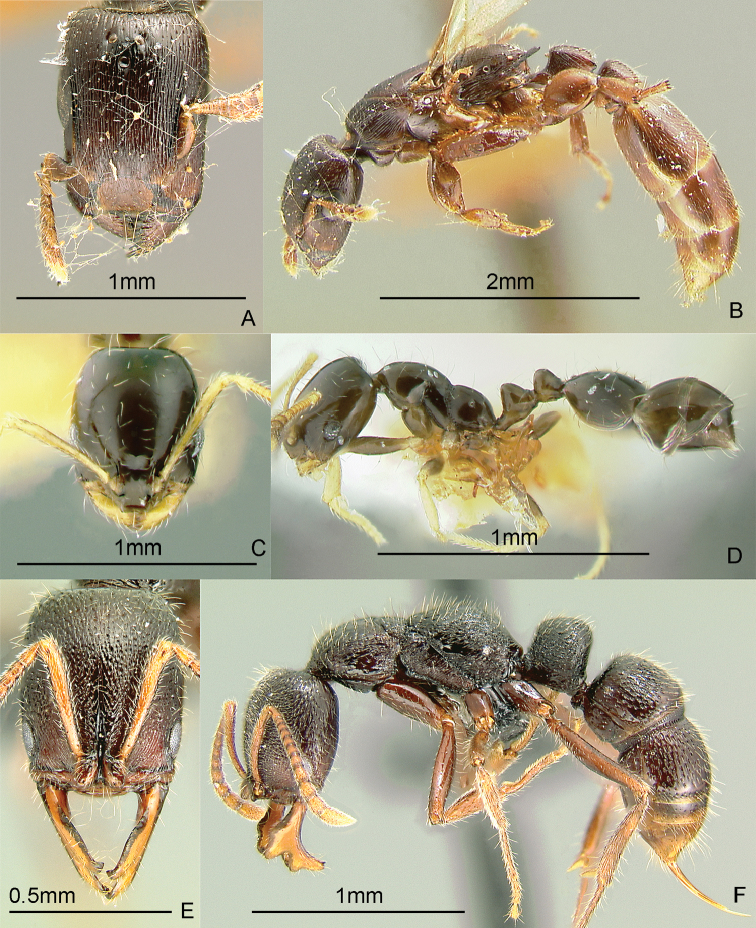
Full-face and profile images of Philippine ant genera. *Metapone gracilis* Wheeler, 1935 **A, B** *Monomorium* species PH01 **C, D** *Myopias lobosa* Willey and Brown, 1983 **E, F**.

### Genus *Monomorium* Mayr, 1855

Myrmicinae: 399 spp., 5 known from PH.

Figs. 23 C, D

These tiny, smooth and slender ants have a single strong seta on the anterior clypeal margin. This genus includes worldwide invasive species such as *Monomorium pharaonis* (Linnaeus, 1758) and *Monomorium floricola* (Jerdon, 1851). They are typically opportunistic predators and scavengers. Their ground-nests are usually marked by a small crater of excavated soil. They may be collected by sifting leaf litter and pitfall trapping and searching for their nests on the ground. Key to species: Heterick (2001) (Australian).

### Genus *Myopias* Roger, 1861

Ponerinae: 36 spp., 5 known from PH.

Figs. 23 E, F

These cryptic ants have eyes that are very close to the base of the mandibles, and usually have a clypeal extension visible in the large gap between the mandibles. They nest in rotten twigs and logs and forage in the leaf litter and on the ground. They are known to prey on springtails, millipedes, and even other ants. These ants may be collected by sifting leaf litter and breaking into rotten wood. Willey and Brown (1983) (Australasian).

### Genus *Myopopone* Roger, 1861

Amblyoponinae: 1 sp., 1 known from PH.

Figs. 24 A, B

The sole species, *Myopopone castanea* (F. Smith 1860b), is widespread in the Philippines. This large-headed species has the characteristic flattening of the antennal flagella. It nests in Zorapteran-stage rotten wood, apparently preying on beetle larvae. It may be collected by breaking open rotten logs that you can dig into with a garden trowel. Brown (1960) (World revision).

**Figure 24.**
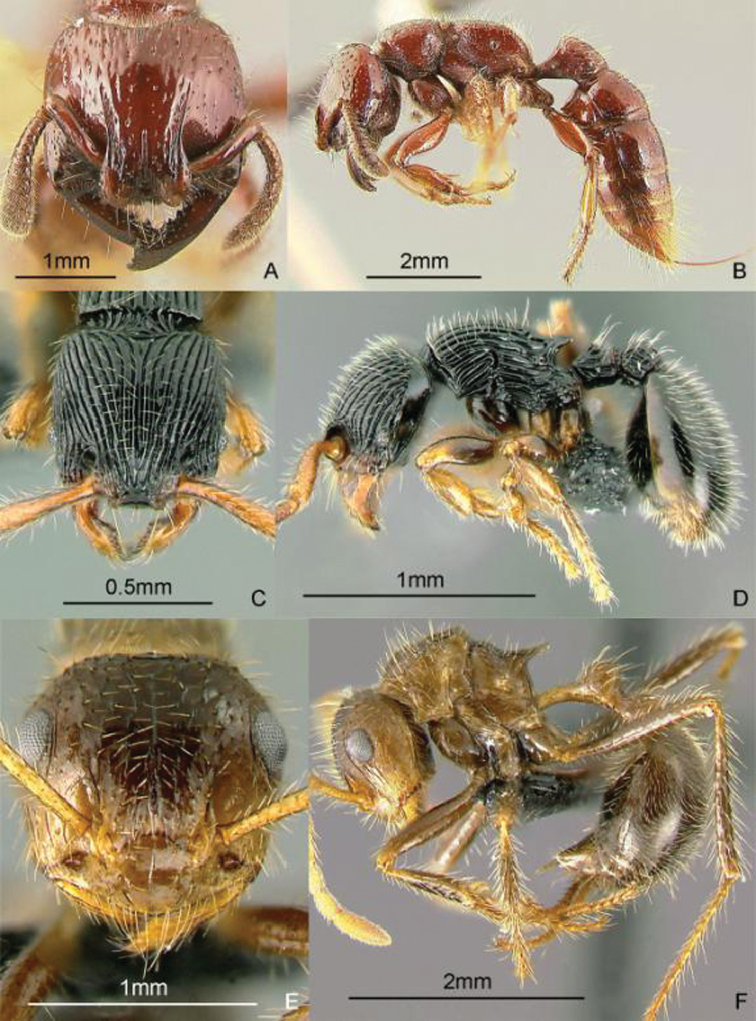
Full-face and profile images of Philippine ant genera. *Myopopone castanea* **A,  B** *Myrmecina* species PH01 **C, D** *Myrmicaria brunnea subcarinata* (F. Smith, 1857)**E, F**.

### Genus *Myrmecina* Curtis, 1829

Myrmicinae: 37 spp., 3 known from PH.

Figs. 24 C, D

This genus is widespread throughout the Philippines. There are specimens of several unidentified species from different transect studies on Mt. Isarog, Bicol Region, Luzon Island (Samson et al. 1997, Caceres in prep., Alpert and General in prep.). One of these species is smooth and reddish-orange and can be mistaken as *Pristomyrmex* ants. There are also specimens of another species from transect studies in the provinces of Bulacan and Nueva Vizcaya, Luzon Island. Specimens of yet another unidentified species were collected in Palawan Island. These hard-bodied ants have the characteristic ventral ridge of the head, running from the back of the head to the base of the mandibles, and the distinctive barrel-shaped petiole. They nest in twigs and rotten wood or under rocks and forage in the leaf litter. These ants may be collected by sifting leaf litter, pitfall trapping, and flipping over rocks.

### Genus *Myrmicaria* Saunders, 1842

Myrmicinae: 67 spp., 3 known from PH.

Figs. 24 E, F

These relatively large ants bear the characteristic 7-segmented antennae and long anterior peduncle of the petiole. They form conspicuous columns, forage on the ground and in the foliage, and are locally dominant where they occur. They are fairly easy to collect by baiting with tuna or honey, pitfall trapping, beating low vegetation, and hand collecting. Santschi (1925) (African; out of date); Bakhtiar et al. (2009) (morphological and behavioral notes of Southeast Asian species).

### Genus *Myrmoteras* Forel, 1893b

Formicinae: 32 spp., 4 known from PH

Figs. 25 A, B

These small trap-jawed ants have long mandibles that look like ripsaws and have very large eyes. They nest and hunt soft-bodied arthropods in the leaf litter. These ants may be collected by inspecting dead leaves that stick together then sifting the leaf litter. Keys to species: Moffett (1985) (World revision), Agosti (1992) (World revision), Zettel and Sorger (2011) (Philippine species).

**Figure 25.**
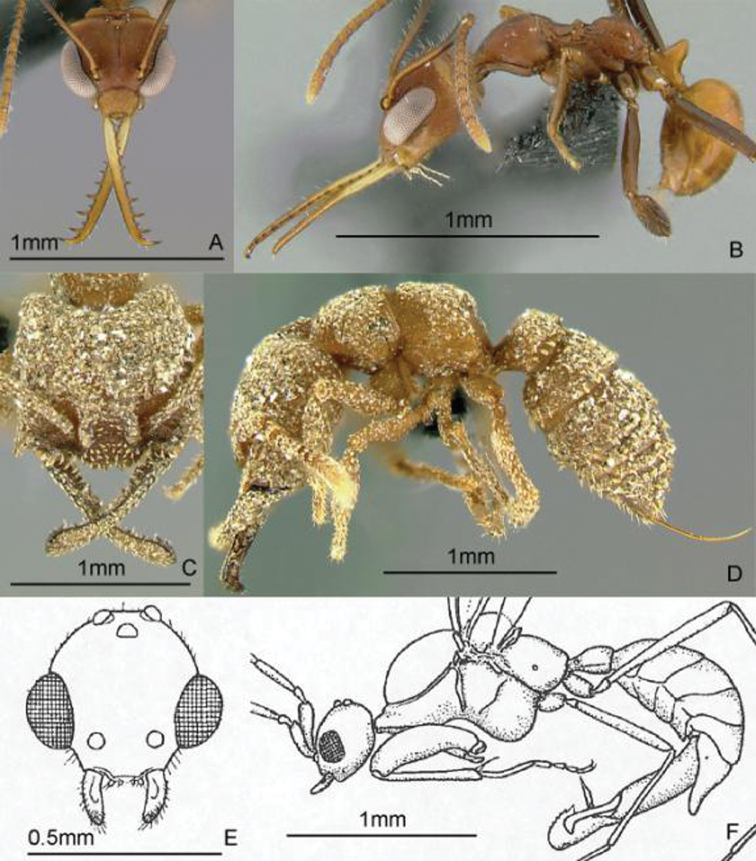
Full-face and profile images of Philippine ant genera. *Myrmoteras wlliamsi* Wheeler, 1919 **A, B** *Mystrium camillae* Emery, 1889 **C, D** *Noonilla copiosa* (line drawings reprinted from Entomologiske Meddelelser, with permission) **E, F**.

### Genus *Mystrium* Roger, 1862

Amblyoponinae: 9 spp., 1 known from PH.

Figs. 25 C, D

These cryptic ants have long, linear mandibles, inserted at the sides of the head, with blunt ends and a snaggle-tooth arrangement. These ground-foraging ants usually have soil particles stuck to their body, providing perfect camouflage. When disturbed, adults lie motionless. They nest under rocks and in rotten wood. These ants may be collected by sifting leaf litter, flipping over rocks, and breaking into rotten logs. Brown (1960) (World revision), Bihn and Verhaagh (2007) (Indo-Australian, with tabular key).

### Genus *Noonilla* Petersen, 1968

Leptanillinae: 1 sp., 2 known from PH.

Figs. 25 E, F

Described from an alate male reproductive, collected in southern Palawan Island, *Noonilla copiosa* Petersen, 1968 is the only described species of this genus. Ogata et al. (1995) examined a male specimen from Misamis Oriental Province, Mindanao Island and considered this genus *incerta sedis* in the subfamily. Bolton (2003) excluded the genus from Formicidae. However, Marek Borowiec (pers. comm.), who is currently studying the subfamily, is convinced that *Noonilla* belongs to Leptanillinae. These ants may be collected by sifting soil cores up to 30 cm deep, underground baiting for workers, and Malaise trapping for alates. Ogata et al. 1995, Bolton (2003) (synopsis of Formicidae).

### Genus *Nylanderia* Emery, 1906

Formicinae: 133 spp., 3 known from PH.

Figs. 26 A, B

This genus was recently revived from synonymy by LaPolla et al. 2010. There are at least 3 unidentified species from a transect study of Mt. Isarog, Bicol Region, Luzon Island (Alpert and General in prep.), including a morphological mimic of *Tapinoma melanocephalum*. These ants typically forage in the foliage, on the ground and in the leaf litter. They feed on honeydew from scale insects and aphids, and scavenge dead insects. These ants may be collected by beating of low vegetation, sifting leaf litter, pitfall trapping, and hand collecting. LaPolla et al. 2010 (Generic key to *Prenolepis* genus-group).

**Figure 26.**
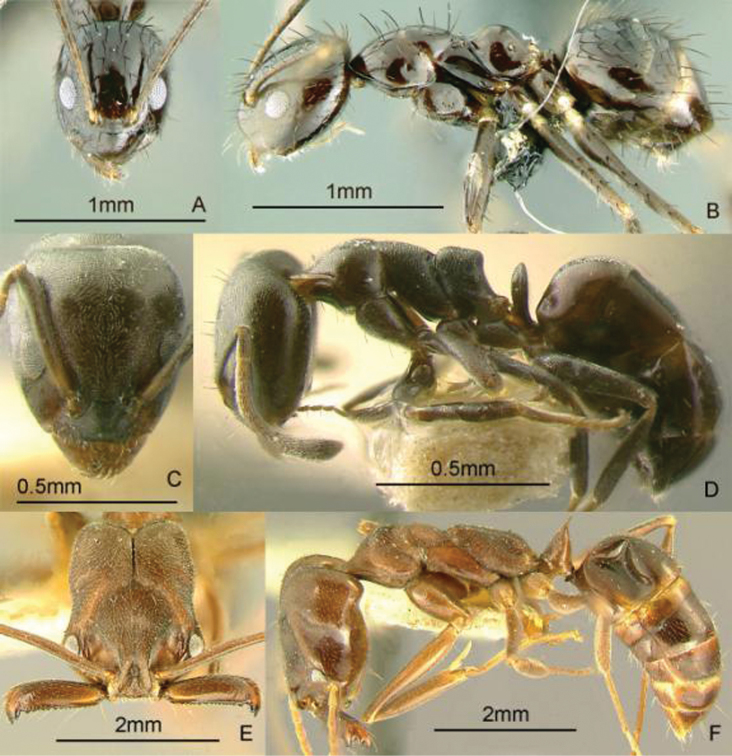
Full-face and profile images of Philippine ant genera. *Nylanderia* species PH01 **A, B** *Ochetellus glaber* **C, D** *Odontomachus simillimus* F. Smith, 1858 **E, F**.

### Genus *Ochetellus* Shattuck, 1992a

Dolichoderinae: 10 spp., 1 known from PH.

Figs. 26 C, D

The only species known from the Philippines, *Ochetellus glaber* (Mayr, 1862), is widespread in the archipelago. These fast-moving ants have a concave anterior clypeal margin and a concave propodeum. They may be arboreal or ground-foraging, sometimes forming conspicuous columns. These ants may be collected by beating of low vegetation, sifting leaf litter, and pitfall trapping. Shattuck (1992a) (generic revision).

### Genus *Odontomachus* Latreille, 1804

Ponerinae: 65 spp., 11 known from PH.

Figs. 26 E, F

These large, big-headed, trap-jawed ants are commonly referred to as “*hantik*” in many Philippine dialects. They are aggressive, locally dominant and ground-dwelling but may also climb the foliage to hunt for prey. One species, *Odontomachus malignus* F. Smith, 1859, is known to nest in rock crevices that are regularly inundated during high tide, whence they emerge and forage for animals trapped in the intertidal zone. Ants of this genus may be collected by baiting with tuna, beating of low vegetation, pitfall trapping, and hand collecting. Keys to species: Brown (1976) (World), Sorger and Zettel (2011) (Philippine species).

### Genus *Odontoponera* Mayr, 1862

Ponerinae: 2 spp., 1 known from PH.

Figs. 27 A, B

*Odontoponera denticulata* (F. Smith, 1858) is widespread in the Philippines. This moderately large, hard-bodied species has large blunt teeth on the sides of the pronotum and a crenulate anterior clypeal margin. It nests under bare ground and hunts singly. The nest entrance is a simple hole just large enough for one worker to pass through. This ant may be collected by sifting leaf litter, pitfall trapping, and hand collecting. Wheeler and Chapman (1925), Creighton (1929) (out of date), Yamane (2009).

**Figure 27.**
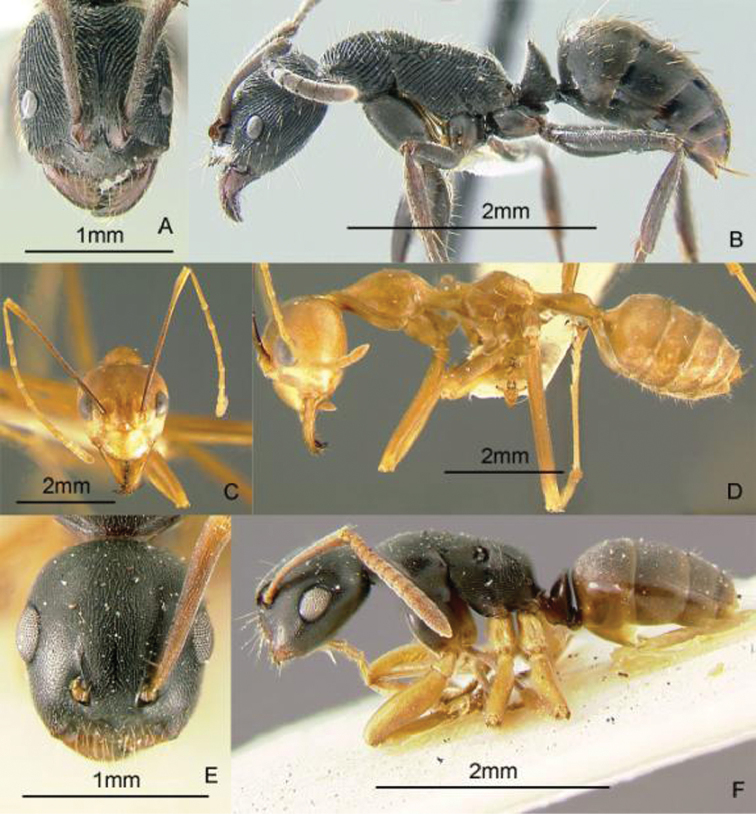
Full-face and profile images of Philippine ant genera. *Odontoponera denticulata* **A, B** *Oecophylla smaragdina* (Fabricius, 1775) **C, D** *Overbeckia subclavata* Viehmeyer, 1916a (N.B. This is a specimen from Singapore. The Philippine specimen has its head crushed on its right side and the mesosoma is damaged as well.) **E, F**.

### Genus *Oecophylla* F. Smith, 1860b

Formicinae: 2 spp., 1 known from PH.

Figs. 27 C, D

*Oecophylla smaragdina* is widespread in the Philippines. This large yellow-green species weaves silken nests among the leaves of mango (Anacardiaceae: *Mangifera indica* L.) and other trees. It is aggressive and dominant where they occur, effectively excluding other ant species in the trees and on the ground as well. A single colony may construct many nests among several trees, with the outer nests serving as defensive bivouacs for older workers and the inner nests containing the queen and brood. *Ilokanos*, many Southeast Asian tribes, and south Chinese tribes harvest the pupae as a delicacy. This species is usually found at elevations below 500 meters. It may be collected by searching for nests in the canopy and hand collecting from the tree trunks.

### Genus *Overbeckia* Viehmeyer, 1916a

Formicinae: 1 sp., 1 known from PH.

Figs. 27 E, F

**(New record).** There is only one valid species in this genus, *Overbeckia subclavata*. This species has an antenna that gradually widens to a relatively broad terminal segment, and has a mesosoma that is flat and pinched at the metapleural spiracles. It was previously known only from Singapore, but a specimen in the MCZ Ant Collection is labeled "Bu of Sci, PI" and was collected by a certain M. Ramos (probably Maximo Ramos, who collected botanical specimens for the Bureau of Science from 1904 to 1932) (Van Steenis-Kruseman 2006). “PI” is the abbreviation for Philippine Islands, the old name used during the American colonial period. *Overbeckia subclavata* is a very rare ant, and its rediscovery will be an important event in Philippine myrmecology. This ant species is assumed to be arboreal because of its relatively large compound eyes. It may be collected by beating low vegetation over a white sheet and inspecting dead branches of living trees.

### Genus *Pachycondyla* F. Smith, 1858

Ponerinae: 289 spp., 16 known from PH.

Figs. 28 A, B

This large genus is also in dire need of taxonomic revision. These are small or large ants with two tibial spurs, a large pectinate spur behind a small simple one. They are abundant in the leaf litter and on the ground, hunting singly. They may be collected by sifting leaf litter, pitfall trapping, and baiting with honey or tuna bait. The genus *Cryptopone* was recently synonymized under *Pachycondyla* by MacKay and MacKay (2010).

**Figure 28.**
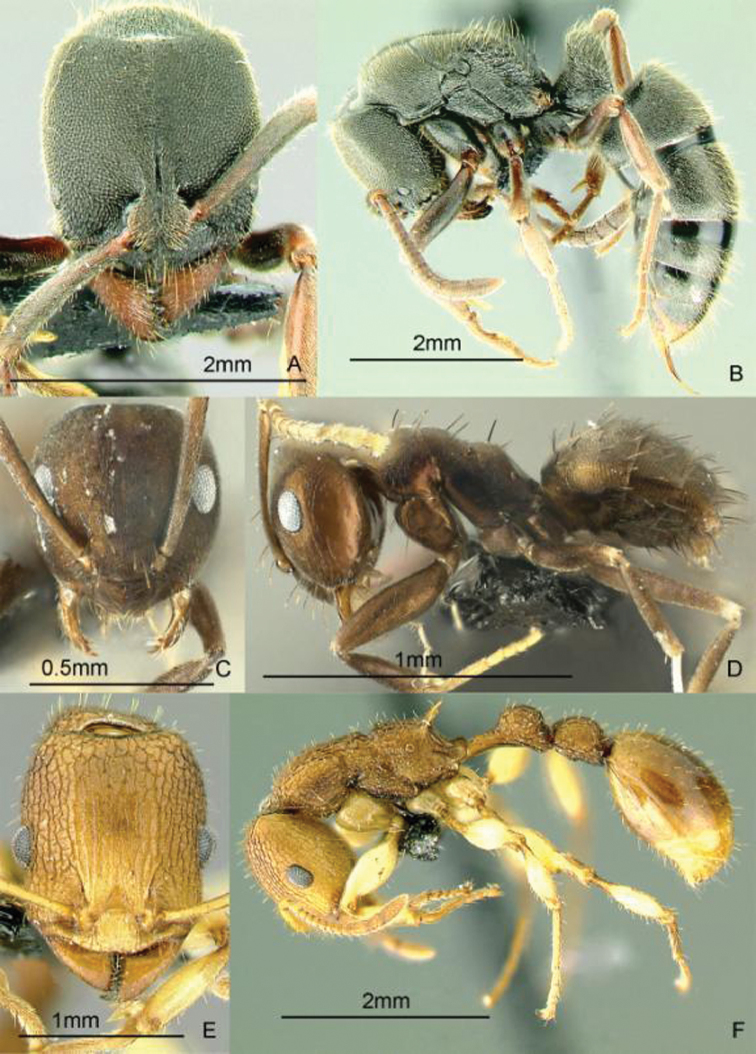
Full-face and profile images of Philippine ant genera. *Pachycondyla claudata* (Menozzi, 1926) **A, B** *Paraparatrechina iridescens* (Donisthorpe, 1942) **C, D** *Paratopula macta* Bolton, 1988 **E, F**.

### Genus *Paraparatrechina* Donisthorpe, 1947

Formicinae: 28 spp, 2 known from PH.

Figs. 28 C, D

This genus was recently revived from synonymy by LaPolla et al. 2010, who split the genus *Paratrechina* into 3 separate genera. Among these 3 genera, only *Paraparatrechina* ants have a pair of erect setae on the propodeum and erect setae on the pronotum and mesonotum arranged in neat pairs. *Paraparatrechina iridescens* and an unidentified species are known from several locations on Luzon Island. These ants may be collected by sifting leaf litter, pitfall trapping, and baiting with honey or tuna bait. LaPolla et al. 2010 (Generic key to *Prenolepis* genus-group).

### Genus *Paratopula* Wheeler, 1919a

Myrmicinae: 10 spp., 3 known from PH.

Figs. 28 E, F

There are ten valid species in this Oriental and Indo-Australian genus, with three species known from the Philippines. These large arboreal ants have a pronotum with a flat dorsal outline. They are rare, and may be collected by beating low vegetation over a white sheet and inspecting tree hollows and dead branches in the canopy. Key to species: Bolton (1988) (World).

### Genus *Paratrechina* Motschoulsky, 1863

Formicinae: 1 sp., 1 known from PH.

Figs. 29 A, B

As revised by LaPolla et al. (2010), this genus now contains only one species, the invasive species, *Paratrechina longicornis* (Latreille, 1802), widespread throughout the Philippines. The species readily invade households, farms and other highly-disturbed areas, recruiting large numbers of nestmates to scavenge dead insects and small animals and even table scraps. It may be collected by sifting leaf litter, pitfall trapping, and baiting with honey or tuna bait. LaPolla et al. 2010 (Generic key to *Prenolepis* genus-group).

**Figure 29.**
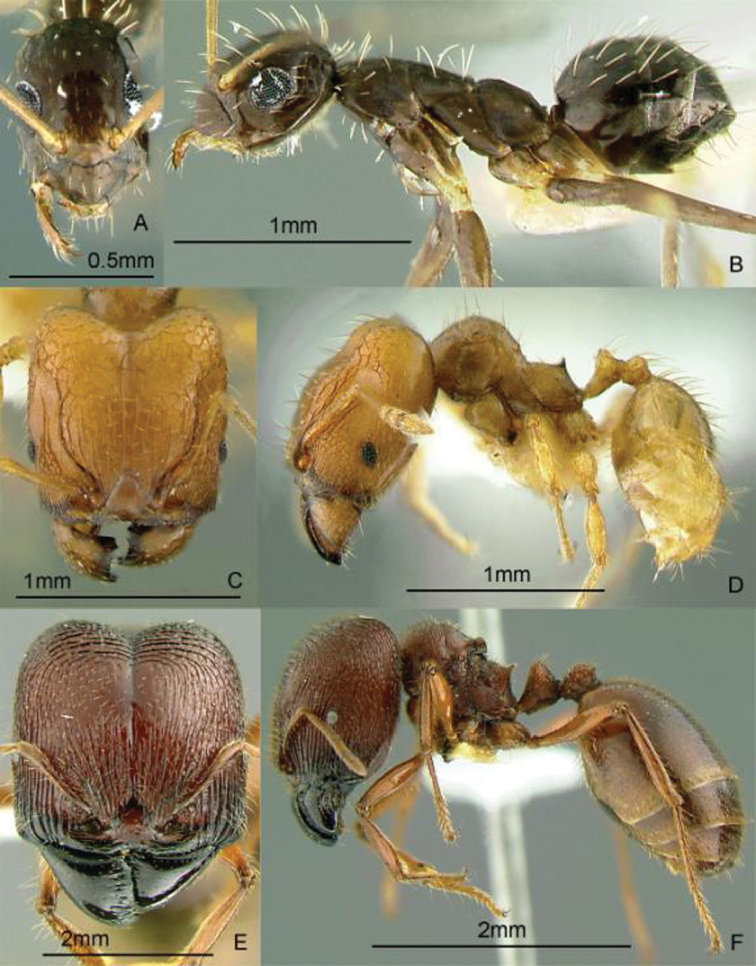
Full-face and profile images of Philippine ant genera. *Paratrechina longicornis* **A, B** *Pheidole hortensis* Forel, 1913, major worker **C, D** *Pheidologeton maccus* Wheeler, 1929, major worker **E, F**.

### Genus *Pheidole* Westwood, 1839

Myrmicinae: 1,121 spp., 21 known from PH.

Figs. 29 C, D

There are specimens of several unidentified species from a transect study of Mt. Isarog, Bicol Region, Luzon Island (Alpert and General in prep.). There are also unidentified species from transect studies in Isabela Province, Luzon Island and the islands of Samar and Mindanao. These tiny to small ants have the following character states: dimorphic, with large-headed major workers possessing usually edentate mandibles; pronotum strongly humped; and antennal club usually 3-segmented. They are ground-dwelling and forage on the ground and in the leaf litter, and may be collected by sifting leaf litter, pitfall trapping, and baiting with cookie crumbs. Eguchi (2001a) (Asian), (2001b) (Bornean), (2003) (morphology of male genitalia), (2004) (revision of *Pheidole fervens* and *Pheidole indica*).

### Genus *Pheidologeton* Mayr, 1862

Myrmicinae: 49 spp., 5 spp. and 4 subspp. known from PH.

Figs. 29 E, F

This is another genus which needs taxonomic revision. These tiny to small ants form conspicuous raiding columns, often protected by low walls of soil. They are extremely polymorphic with a continuous series of intermediates. Superficially similar to *Pheidole*, they can be distinguished by their 2-segmented antennal club and their polymorphism. And unlike *Pheidole*, the major and supermajor workers join the foraging column. They are ground-dwelling, and may be collected by sifting leaf litter, pitfall trapping, baiting with honey or tuna bait, and hand collecting. Ettershank (1966).

### Genus *Philidris* Shattuck, 1992a

Dolichoderinae: 16 spp., 2 known from PH.

Figs. 30 A, B

*Philidris myrmecodiae* (Emery, 1887) was reported from Mt. Isarog, Camarines Sur, Luzon Island by Samson et al. (1997). There are specimens of an unidentified species, collected from Zamboanga, Mindanao Island, in the MCZ Ant Collection. There are also specimens from a transect study conducted in Isabela, Luzon Island. An entire nest series of an unidentified species was recently collected from a small island off eastern Mindanao (DMG, unpubl. notes). These small polymorphic ants are superficially similar to *Iridomyrmex* but have their eyes very low on the head. They nest in rotten wood or carton above the ground and in swollen specialized plant structures called domatia, which workers defend vigorously (Shattuck and Barnett 2010). They may be collected by beating low vegetation over a white sheet and inspecting the swollen bases of epiphytic plants. Shattuck (1992a, 1992b).

**Figure 30.**
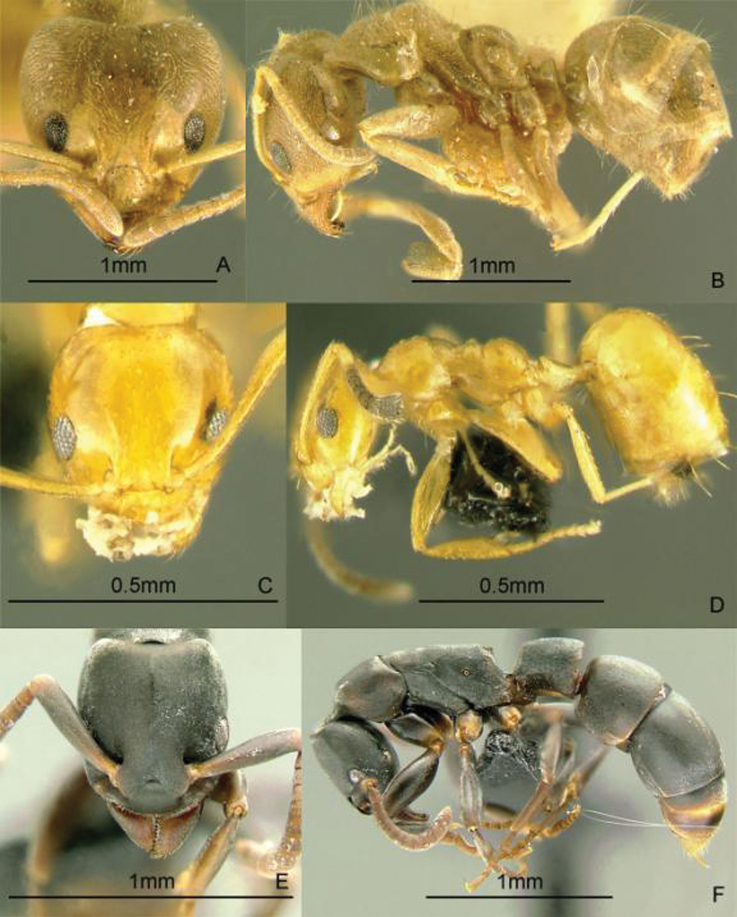
Full-face and profile images of Philippine ant genera. *Philidris* species PH01 **A, B** *Plagiolepis* species PH01 **C, D** *Platythyrea parallela* (F. Smith, 1859) **E, F**.

### Genus *Plagiolepis* Mayr, 1861

Formicinae: 86 spp., 2 known from PH.

Figs. 30 C, D

**(New record).** There are specimens of an unidentified species from a transect study of Mt. Isarog, Bicol Region, Luzon Island (Alpert and General in prep.) and of other species from transect studies conducted by Perry Buenavente in the provinces of Isabela and Nueva Vizcaya, Luzon Island. These tiny, cryptic ants have 11-segmented antennae and long palps. They nest in the ground under rocks and in rotten wood and forage in the leaf litter, on the ground, and in the foliage. These ants may be collected by sifting leaf litter, pitfall trapping, and beating low vegetation over a white sheet. Brown (1973).

### Genus *Platythyrea* Roger, 1863

Ponerinae: 46 spp., 4 known from PH.

Figs. 30 E, F

There are 46 valid species, including three from amber, in this cosmotropical genus. There are four valid species known from the Philippines. These ants have a shagreened or dull body surface and two pectinate tibial spurs on the hind leg. They may be ground-dwelling or nesting in trees, and are often found hunting individually. They may be collected by sifting leaf litter, pitfall trapping, and hand collecting on tree trunks. Key to species: Brown (1975) (World).

### Genus *Polyrhachis* F. Smith, 1857

Formicinae: 603 spp., 75 sp. and 10 subspp. known from PH.

Figs. 31 A, B

This is the largest ant genus in the Philippines. There are specimens of several unidentified species from a transect study of Mt. Isarog, Bicol Region, Luzon Island (Alpert and General in prep.). These small to large ants have spines on the pronotum, mesonotum, propodeum, or petiole or a combination of locations. They may nest in the ground, rotten logs or tree hollows. Some species are also known as weaver ants because they build nests in the foliage from larval silk and chewed-up plant fibers. These ants may be collected by beating low vegetation over a white sheet, pitfall trapping, and inspecting tree hollows and dead branches in the canopy. Keys: Hung (1967), Dorow (1995), (World, subgenera); Bolton (1975), Kohout (1987) (*sexspinosa* species-group), Dorow and Kohout (1995), Kohout (1998), Kohout (2006a) (*cryptoceroides* species-group), (2006b) (*parabiotica* species-group). Natural history: Dorow et al. 1990.

**Figure 31.**
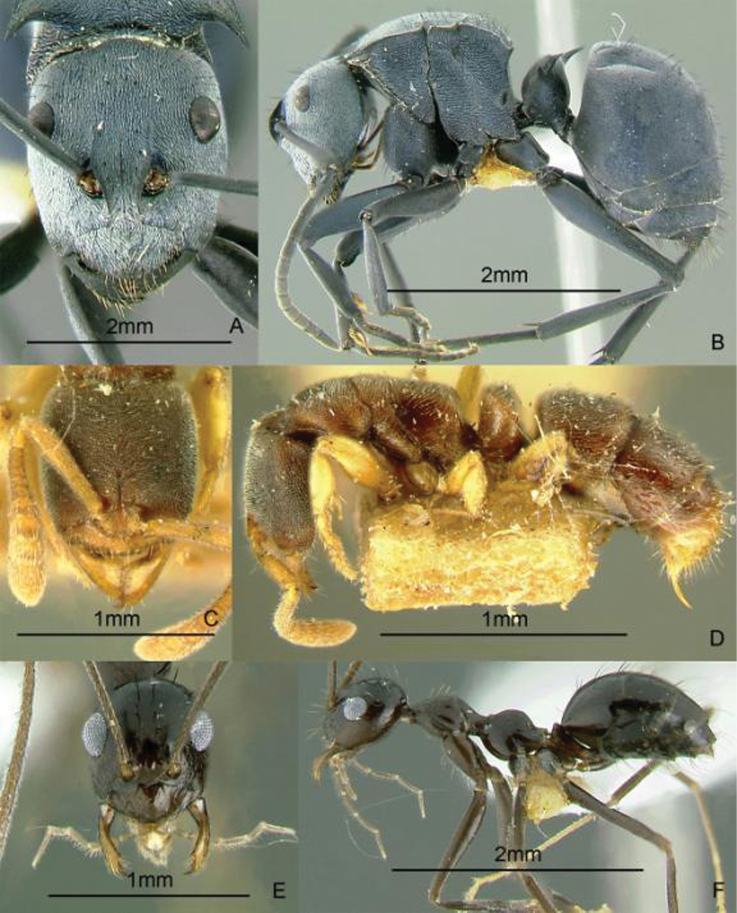
Full-face and profile images of Philippine ant genera. *Polyrhachis cyaniventris* F. Smith, 1858 **A, B** *Ponera oreas* (Wheeler, 1933) **C, D** *Prenolepis* species PH01 **E, F**.

### Genus *Ponera* Latreille, 1804

Ponerinae: 55 spp., 2 known from PH.

Figs. 31 C, D

These tiny cryptic ants may be confused with *Hypoponera* or *Pachycondyla*, but have a fenestra or translucent window in the ventral petiolar process. They forage in the leaf litter and on the ground and nest in rotten wood and under rocks. These ants may be collected by sifting leaf litter, pitfall trapping, breaking into rotten wood, and flipping over rocks. Key to species: Taylor (1967) (World, revision).

### Genus *Prenolepis* Mayr, 1861

Formicinae: 25 spp., 1 known from PH.

Figs. 31 E, F

There are specimens of an unidentified species from a transect study of Mt. Isarog, Bicol Region, Luzon Island (Alpert and General in prep.). These ants have the following character states: very long antennal scapes, at least half the length extending beyond the back of the head; mandibles not strongly curved, so that the apical tooth points to the side; and mesosoma elongated and constricted at midlength. They are ground-foraging. These ants may be collected by sifting leaf litter and pitfall trapping. LaPolla et al. 2010 (Generic key to *Prenolepis* genus-group).

### Genus *Prionopelta* Mayr, 1866a

Amblyoponinae: 13 spp., 1 known from PH.

Figs. 32 A, B

*Prionopelta kraepelini* Forel, 1905 was collected from a botanical transect study on Samar Island. This species is widespread, also known from several locations on the islands of Luzon, Negros and Palawan. Specimens are deposited in the National Museum of the Philippines in Manila, the UPLB-MNH, and the MCZ Ant Collection. These tiny, cryptic ants have mandibles with only 3 teeth. The workers forage in leaf litter and on the ground. These ants may be collected by sifting leaf litter and pitfall trapping. Keys to species: Brown (1960) (Indo-Australian, Neotropical), Shattuck (2008a) (Indo-Pacific).

**Figure 32.**
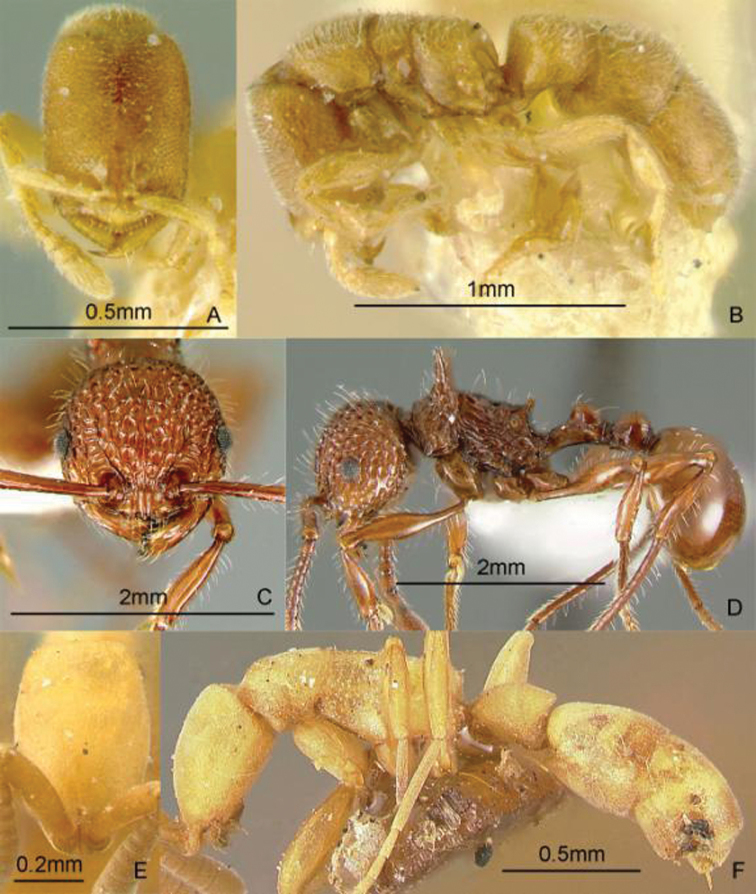
Full-face and profile images of Philippine ant genera. *Prionopelta kraepelini* **A, B** *Pristomyrmex bicolor* Emery, 1900 **C, D** *Probolomyrmex dammermani* Wheeler, 1928 **E, F**.

### Genus *Pristomyrmex* Mayr, 1866b

Myrmicinae: 56 spp., 18 known from PH.

Figs. 32 C, D

There are specimens of an unidentified species from a transect study of Mt. Isarog, Bicol Region, Luzon Island (Alpert and General in prep.). These small attractive ants have the following character states: mandibles that are twisted so that the edges oppose each other; and exposed antennal sockets. They nest in rotten wood on the ground or under rocks and forage in the leaf litter and on the ground. These ants may be collected by sifting leaf litter, pitfall trapping, breaking into rotten logs and flipping over rocks. Keys to species: Wang (2003) (World revision), Zettel (2006, 2007) (Philippine species).

### Genus *Probolomyrmex* Mayr, 1901

Proceratiinae: 16 spp., 2 known from PH.

Figs. 32 E, F

One species is known from Negros Island, *Probolomyrmex dammermani*.There are specimens of an unidentified species collected by Joanaviva Caceres-Plopenio from a transect study of Mt. Isarog, Bicol Region, Luzon Island (Eguchi, pers. comm.; DMG, unpubl. notes). These tiny, cryptic ants have no eyes and bear their thick antennae on a shelf projecting forward from the head. They forage in the leaf litter and presumably also in the soil. These are among the rarest ants in the world. These ants may be collected by sifting leaf litter. Status of genus: Brown (1975). Keys to species: Taylor (1965), Eguchi et al. 2006.

### Genus *Proceratium* Roger, 1863

Proceratiinae: 80 spp., 1 known from PH.

Figs. 33 A, B

The species known from the Philippines, *Proceratium papuanum* Emery, 1897a, was collected from a transect study of Mt. Isarog, Bicol Region, Luzon Island (Alpert and General in prep.). These tiny ants have the following character states: apical segment of the antenna not extremely large or bulbous; and the petiole narrowly attached to the gaster. The workers forage in the leaf litter and on the ground. They may be collected by pitfall trapping and sifting leaf litter. Key to species: Baroni Urbani and de Andrade (2003) (World revision, including fossils).

**Figure 33.**
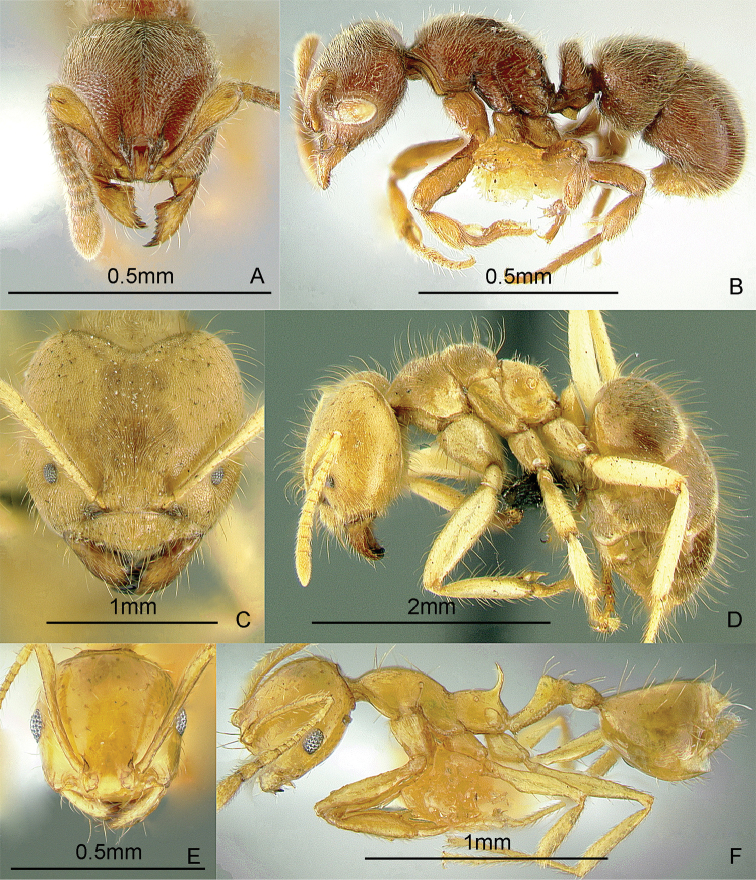
Full-face and profile images of Philippine ant genera. *Proceratium papuanum* **A, B** *Pseudolasius typhlops*, major worker **C, D** *Recurvidris* species PH01 **E, F**.

### Genus *Pseudolasius* Emery, 1887b

Formicinae: 64 spp., 2 known from PH.

Figs. 33 C, D

This genus can be found in all the Old World tropics except Madagascar. *Pseudolasius typhlops* Wheeler, 1935b is known only from the Philippines. There are historical specimens of a different, unidentified species in the ant collection of the UPLB-MNH, and at least one unidentified species reported by Samson et al. 1997. Other unidentified species were recently collected from other parts of Luzon and from Mindanao Island. These are tiny to small, yellow polymorphic ants. They nest in rotten wood on the ground or underground, where they tend root-feeding coccids. These ants may be collected by sifting leaf litter and soil cores and underground baiting.

### Genus *Recurvidris* Bolton, 1992

Myrmicinae: 9 spp., 2 known from PH.

Figs. 33 E, F

There are specimens of at least 2 unidentified species from a transect study in Mt. Isarog, Bicol Region, Luzon Island (Alpert and General in prep.). There are also unidentified specimens from Mindanao Island. Zettel (2008) recently described *Recurvidris nigrans* from Negros Island. These small, slender ants have propodeal spines that curve upward and forward. They are ground-dwelling and forage in the leaf litter, and may be collected by sifting leaf litter, underground baiting and pitfall trapping. Keys to species: Bolton (1992), Zettel (2008).

### Genus *Rhopalomastix* Forel, 1900a

Myrmicinae: 7 spp., 1 known from PH.

Figs. 34 A, B

**(New record).** There are specimens of an unidentified species from Dumaguete, Negros Island in the MCZ Ant Collection. This species has a longitudinally striate head and mesosoma, and a postpetiole broadly attached to the gaster. It nests and forages in tunnels under bark. These ants are rare and may be collected by searching under the bark of living trees (DMG, GDA, unpubl. notes). Wheeler (1929c) (Review of genus).

**Figure 34.**
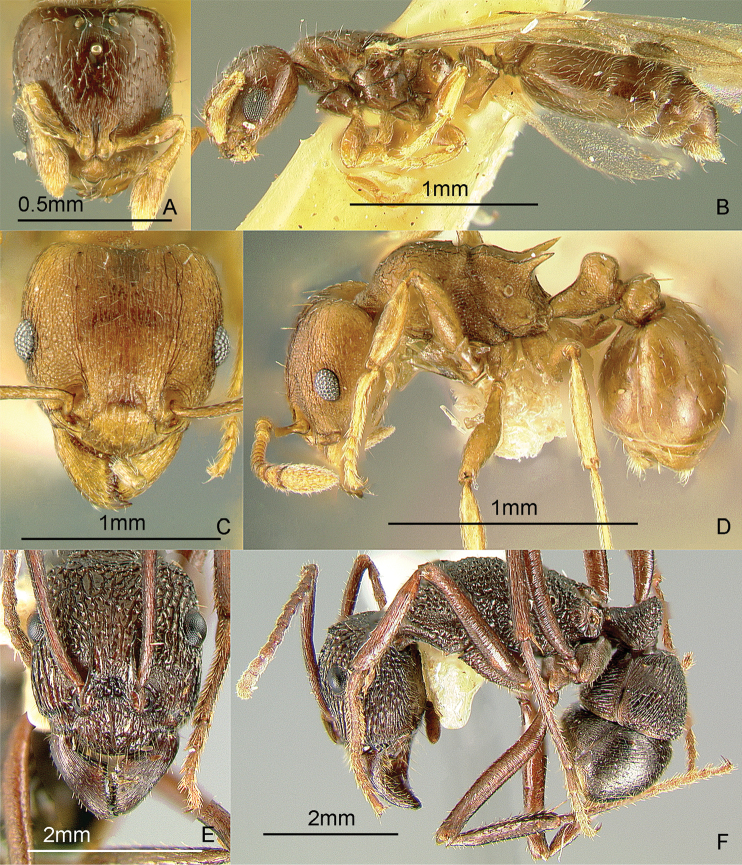
Full-face and profile images of Philippine ant genera. *Rhopalomastix* species PH01, queen **A, B** *Rhoptromyrmex wroughtonii* **C, D** *Rhytidoponera* species PH01 **E, F**.

### Genus *Rhoptromyrmex* Mayr, 1901

Myrmicinae: 10 spp., 1 known from PH.

Figs. 34 C, D

There is one specimen, from “Boguio” [=Baguio City, Benguet Province, Luzon Island], of *Rhoptromyrmex wroughtonii* Forel, 1902a in the MCZ Ant Collection. This species was recently found to be abundant by Joanaviva Caceres-Plopenio during a transect study of an abandoned farm on Mt. Isarog, Camarines Sur, Luzon Island and was also collected by Perry Buenavente from a transect study on Mt. Palali, Nueva Vizcaya, Luzon Island (DMG, unpubl. notes). These small ants share similar morphological features with *Tetramorium* spp., e.g., a sharp clypeal ridge in front of antennal sockets and a lamella at the tip of the sting, but have a heart-shaped head, a broader, convex clypeus, and a keel-like petiolar venter. They are known to form large, ground-dwelling colonies nesting in rotten logs or in the ground and may be collected by sifting leaf litter and pitfall trapping. Keys to species: Brown (1964) (World revision); Bolton (1976) (World tribal revision), (1986) (World revision).

### Genus *Rhytidoponera* Mayr, 1862

Ectatomminae: 105 spp., 3 known from PH.

Figs. 34 E, F

Brown (1958) reports one species from the Philippines, *Rhytidoponera araneoides* (Le Guillou, 1842). There are specimens of an unidentified species from “San Francisco, Agusan”, Mindanao Island, in the MCZ Ant Collection. These hard-bodied ants are superficially similar to *Gnamptogenys* spp. but have a tooth at the anterolateral edge of the pronotum, and a triangular petiole in lateral view. They are generalist predators or scavengers and nest in the ground under rocks but may also be arboreal, and may be collected by sifting leaf litter, pitfall trapping, beating low vegetation over a white sheet and inspecting tree hollows and dead branches in the canopy.

### Genus *Romblonella* Wheeler, 1935a

Myrmicinae: 9 spp., 1 known from PH.

Figs. 35 A, B

There is one valid species known from the Philippines, *Romblonella opaca* (F. Smith, 1861). It is a widespread species, having been collected in the far-apart islands of Romblon, Rapu-rapu, and Palawan (DMG, unpubl. notes). This species has a large, bulbous, sessile petiole with a tiny denticle on the lower surface. It seems to be tolerant of human disturbance because the Rapu-rapu specimen was collected on a concrete sidewalk. Ants of this genus are ground-dwelling and may be collected by sifting leaf litter, pitfall trapping, and hand collecting. Keys to species: Bolton (1976) (World revision), Taylor (1991a) (Australian).

**Figure 35.**
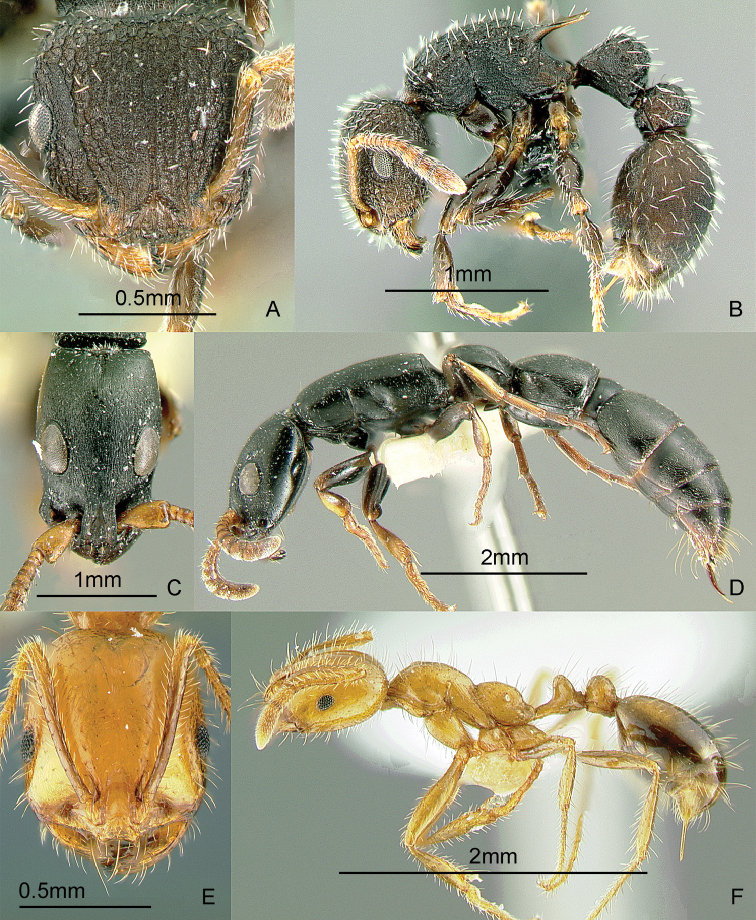
Full-face and profile images of Philippine ant genera. *Romblonella opaca* **A, B** *Simopone conradti* Emery, 1899 (N.B. Not from the Philippines, the unique holotype of *Simopone chapmani* is badly broken and cannot help the student visualize this rare genus) **C, D** *Solenopsis geminata* minor worker **E, F**.

### Genus *Simopone* Forel, 1891

Cerapachyinae: 16 spp., 2 known from PH.

Figs. 35 C, D

The unique holotype specimen of *Simopone chapmani* Taylor, 1966, is deposited in the MCZ Ant Collection. These small ants bear a long barrel-shaped petiole and no tibial spurs on the middle legs. They are often arboreal, and presumed to be predators on other ants, and may be collected by beating low vegetation over a white sheet and examining dead twigs or branches in the canopy.

### Genus *Solenopsis* Westwood, 1840

Myrmicinae: 285 spp., 2 known from PH.

Figs. 35 E, F

Ants of this genus exhibit two main lifeways: the fire ants live independently in familiar ant mounds while the tiny thief ants nest beside other ants or termites. The species known in the Philippines, *Solenopsis geminata*, also called fire ants, is a common pest in households and agricultural areas. It is highly polymorphic, with a wide range of sizes between major and minor workers. There are unidentified specimens of *Solenopsis* subgenus *Diplorhoptrum*, which are tiny, monomorphic ants, collected by Perry Buenavente from a transect studies on Mt. Palali, Nueva Vizcaya Province, Luzon Island and 2 mountains in Mindanao Island (DMG, unpubl. notes). There are also specimens of an unidentified species from a transect study of Mt. Isarog, Bicol Region, Luzon Island (Alpert and General in prep.). These ants may be collected by sifting leaf litter, pitfall trapping, and hand collecting. Key to species: Trager (1991) (World, *geminata-*group).

### Genus *Stigmatomma* Roger, 1859

Amblyoponinae: 66 spp., 3 known from PH.

Figs. 10 A, B

Yoshimura and Fisher (2012) recently revived the status of this genus from synonymy. Philippine ants formerly referable to *Amblyopone* Erichson, 1842 are now transferred to this genus. These cryptic ants have characteristic elongate mandibles and a denticulate anterior clypeal border. In these ants, the gaster is broadly attached to the petiole. They are found under the bark of logs, in rotten wood, leaf litter, or soil and are known to prey on centipedes and beetle larvae. Key to species (as *Amblyopone*): Taylor (1979) (Melanesia).

### Genus *Strumigenys* F. Smith, 1860c

Myrmicinae: 774 spp., 30 spp. and 1 subsp. known from PH.

Figs. 36 A, B

There are specimens of an unidentified species from a transect studies of Mt. Isarog, Bicol Region, Luzon Island (Alpert and General in prep., Caceres-Plopenio, unpubl. M.S. thesis). There are also specimens of unidentified species from Eastern Samar Province, Samar Island and Polillo Island. These tiny to small, cryptic ants have spongy lobes at the sides of the propodeum, petiole, and postpetiole. They hunt for soft-bodied arthropod prey, usually *Collembola* (springtails), and nest in the leaf litter. These ants may be collected by inspecting rotten woody debris and dead leaves that are stuck together for nests before sifting leaf litter. Key to species: Bolton (2000) (World revision), Baroni Urbani and de Andrade 2007 (synonymy of *Pyramica* under *Strumigenys*).

### Genus *Tapinoma* Foerster, 1850

Dolichoderinae: 93 spp., 4 known from PH.

Figs. 36 C, D

The species known from the Philippines include the widespread invasive species, *Tapinoma melanocephalum* (Fabricius, 1793), which commonly invades households in search of sweet food. These small ants have a small, flat petiole and reflexed apex of the gaster, such that only four segments are visible in dorsal view. They may forage in the foliage or on the ground for dead insects but also tend coccids and aphids for honeydew. They nest in just about any available cavity in soil, under stones and bark, in living plants, and houses, and may be collected by sifting leaf litter, beating low vegetation over a white sheet, and baiting with sugar. Shattuck (1999), Fisher and Bolton (2007).

**Figure 36.**
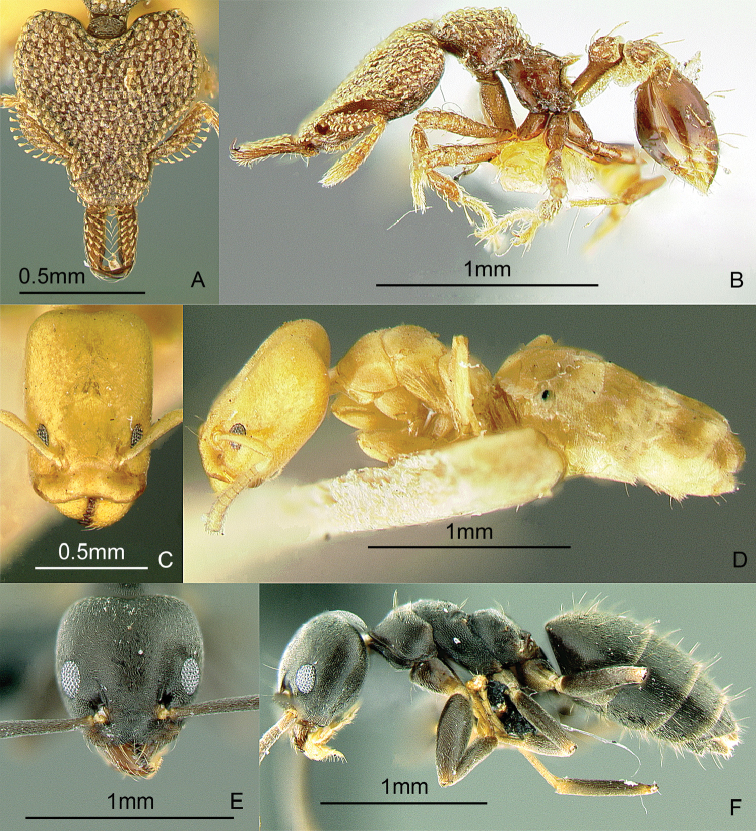
Full-face and profile images of Philippine ant genera. *Strumigenys chapmani* Brown, 1954 **A, B** *Tapinoma williamsi* **C, D** *Technomyrmex albipes* (F. Smith, 1861) **E, F**.

### Genus *Technomyrmex* Mayr, 1872

Dolichoderinae: 90 spp., 10 known from PH.

Figs. 36 E, F

The species known from the Philippines include the widespread invasive species, *Technomyrmex albipes* (F. Smith, 1861), which is very common in highly disturbed or agricultural areas. These ants also have a small, flat petiole but with five visible gastral segments in dorsal view. It can be difficult to distinguish them from *Tapinoma*, especially in shrunken or deformed specimens. They are scavengers and forage in the foliage and on the ground. They nest in the soil, in rotten wood, and under bark and rocks. These ants may be collected by sifting leaf litter, pitfall trapping, beating low vegetation over a white sheet, hand collecting, and flipping over rocks and loose bark. Key to species: Bolton (2007) (World revision).

### Genus *Tetramorium* Mayr, 1855

Myrmicinae: 459 spp., 28 from PH.

Figs. 37 A, B

The Philippine species include widespread invasive species such as *Tetramorium lanuginosum* Mayr, 1870, which are common in highly disturbed or agricultural areas (Wetterer, 2010). Schlick-Steiner et al. 2006 revived *Tetramorium manobo* (Calilung, 2000) from synonymy. There are specimens of several undescribed species (including a species misidentified as *Leptothorax* by Samson et al. 1997) from a transect study on Mt. Isarog, Bicol Region, Luzon Island (Alpert and General, in prep.). These small ants have a sharp clypeal ridge in front of the antennal sockets and a lamella at the tip of the sting, but the head is roughly rectangular in shape and with a straight-edged or slightly concave anterior clypeal margin. They are variable in size, sculpture of the cuticle, and color. These ants are generalist predators and scavengers, foraging on the ground, and nest in twigs, rotten wood, under rocks or bark. They may be collected by sifting leaf litter, pitfall trapping, breaking into rotten wood and twigs, and flipping over rocks and loose bark. Keys to species: Bolton (1976, 1977) (Oriental, Indo-Australian), Schlick-Steiner et al. 2006.

**Figure 37.**
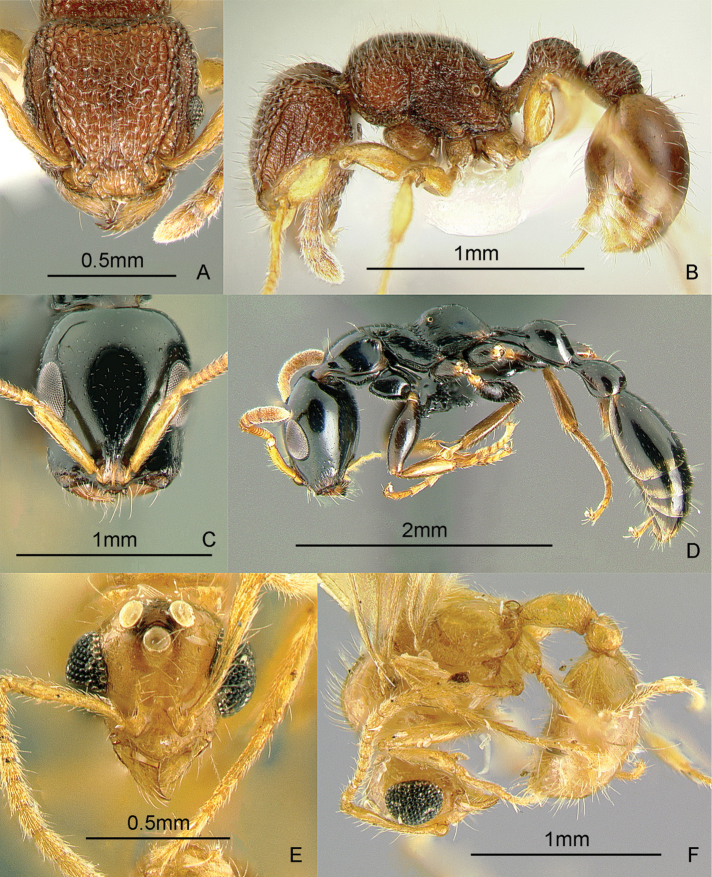
Full-face and profile images of Philippine ant genera. *Tetramorium khnum* Bolton, 1977 **A,  B** *Tetraponera allaborans* (Walker, 1859) **C, D** *Tyrannomyrmex* species PH01 **E, F**.

### Genus *Tetraponera* F. Smith, 1852

Pseudomyrmicinae: 118 spp., 9 known from PH.

Figs. 37 C, D

These long and slender arboreal ants have large, somewhat flattened eyes, and a reduced clypeus, such that the antennal sockets are near the front edge of the head. They nest in dead twigs and branches. These ants may be collected by beating low vegetation over a white sheet and inspecting dead twigs and branches in the canopy. Key to species: Ward (2001) (Oriental, Australian).

### Genus *Tyrannomyrmex* Fernandez, 2003

Myrmicinae: 3 sp., 1 known from PH.

Putative male alate: Figs. 37 E, F

This is a genus that is very rarely collected. The three known species were all described from unique worker specimens. A single male specimen, in the Philippine collection of the Bernice P. Bishop Museum of Hawaii, appears to belong to this genus because its mandible has only the apical and subapical teeth followed by a wide toothless masticatory margin and its curved petiole lacks a distinct node. If confirmed, it will be the first known male specimen of *Tyrannomyrmex*. These small ants have a curved petiole surmounted by a low node, and strongly-curved mandibles with only two teeth. They likely forage deep in the soil and only occasionally emerge in the leaf litter. These ants may be collected by underground baiting, sifting leaf litter and pitfall trapping. Fernandez (2003), Bolton et al. 2006.

### Genus *Vollenhovia* Mayr, 1865

Myrmicinae: 72 spp., 6 known from PH.

Figs. 38 A, B

These flat and slender ants have a large, keel-like ventral petiolar process. They nest in rotten wood and under rocks and forage in leaf litter. Little is known about their biology. They may be collected by sifting leaf litter, pitfall trapping, breaking into rotten wood and flipping over rocks. Bolton (2003).

**Figure 38.**
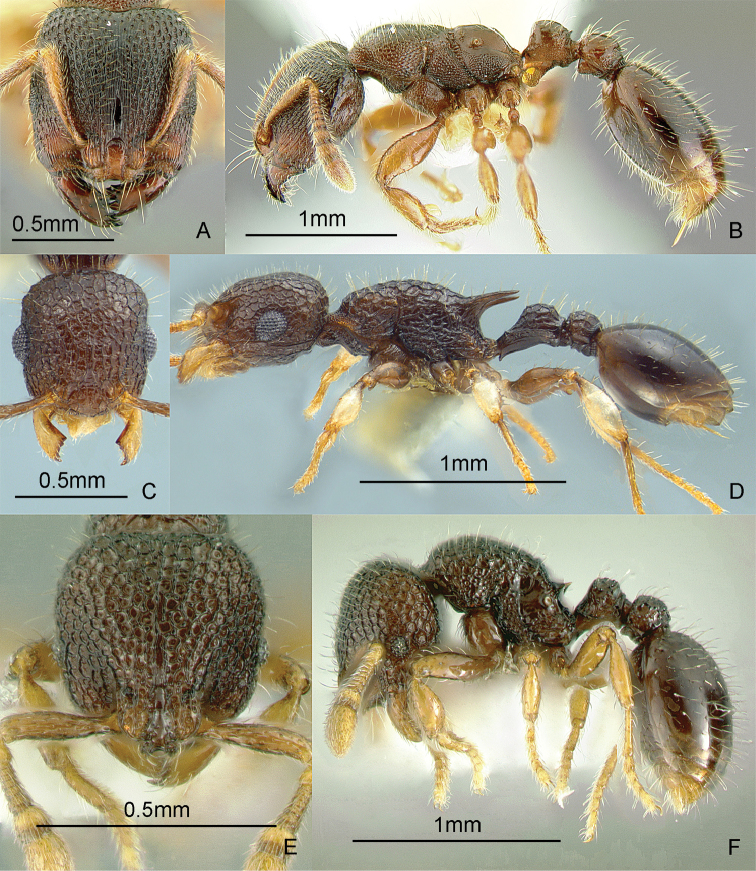
Full-face and profile images of Philippine ant genera. *Vollenhovia ambitiosa* Menozzi, 1925 **A, B** *Vombisidris philippina* Zettel and Sorger, 2010 (image reproduced with permission of NHMW and antbase.net) **C, D** Unnamed genus PH01 **E, F**.

### Genus *Vombisidris* Bolton, 1991

Myrmicinae: 16 spp., 2 known from PH.

Figs. 38 C, D

A recently described species, *V*, was collected from five locations on three different islands, representing the first record of this genus in the Philippines (Zettel and Sorger 2010a). They also report at least another species, represented by a dealate queen from yet another location. These small ants are recognizable by a sinuate subocular groove running from the mandibular insertion to the latero-occipital margin of the head. Ants of this genus are arboreal, at least one species (*Vombisidris humboldticola* Zacharias & Rajan, 2004 from southern India) nesting in domatia, which are specialized swollen plant structures for harboring ants. These ants may be collected by beating low vegetation over a white sheet and inspecting domatia of epiphytes. Zettel and Sorger (2010a).

### Unnamed genus PH01

Myrmicinae: 1 sp., 1 known from PH.

Figs. 38 E, F

**(New record).** Specimens were extracted from berlesate from three locations on Samar Island. In Bolton (1994), this ant keys out to *Mayriella*, but bears little resemblance to it. The ant more closely resembles *Tetheamyrma* with its bidentate clypeal projection (S. Cover, pers. comm.), but has 10-segmented antennae and has no spatulate or lamellate hairs on the inner margin of the mandibles. Images are available online at: http://pick4.pick.uga.edu/mp/20q?act=x_antandpath=Insecta/Hymenoptera/Formicidae/Adelomyrmex/sp_phi1andname=Adelomyrmex+sp_phi1andxml=Ants_Philippinesandauthority=unknown+species. It may be collected by sifting leaf litter and pitfall trapping.

### Unnamed genus PH02

Myrmicinae: 1 sp. (?), 1 known from PH.

Figs. 39 A, B

**(New record).** There is a single specimen of this undescribed genus in the Philippine collection of the Bernice P. Bishop Museum in Honolulu, Hawaii. The collection was loaned to GDA, who found this unique specimen. The specimen was collected by L.W. Quate from San Francisco, Agusan del Sur in 1959. In Bolton (1994), this specimen keys out to “Undescribed genus” (p. 89) (P. Ward and S. Cover, pers.comm.). The diagnostic characteristic of this genus is the presence of the petiolar spiracle in the node, rather than in the peduncle. No collection information is included with the specimen, however, applying different collecting techniques may discover this species.

**Figure 39.**
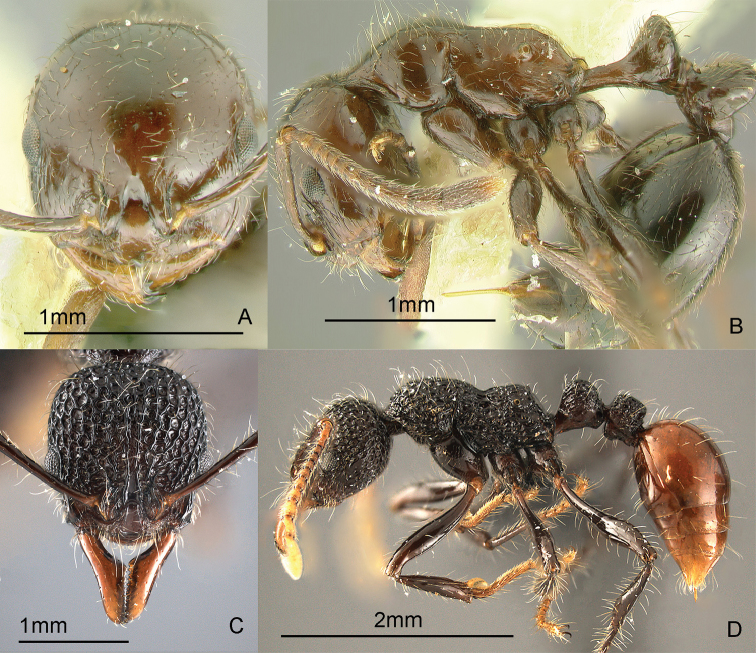
Full-face and profile images of Philippine ant genera. Unnamed genus PH02 **A, B** Unnamed genus PH03 **C, D**.

### Unnamed genus PH03

Myrmicinae: 2 spp., 2 known from PH.

Figs. 39 C, D

**(New record).** A good nest series of this undescribed genus was collected by Perry Buenavente in a transect study of Mt. Palali, Nueva Vizcaya Province, Luzon Island. A unique specimen of another species (now lost due to theft, Taylor, pers. comm.) was earlier collected by Bambet Alto from Mt. Isarog. These reddish-orange ants seem to prefer high elevations, having been collected from >1450 masl at both locations. These ants may nest in rotten wood (DMG, unpubl. notes). They are characterized by stemmed mandibles with blunt or rounded denticles and a subsessile petiole with an angle protecting the spiracle. These ants may be collected by breaking into rotten wood, sifting leaf litter and pitfall trapping.

### Key to the subfamilies of Philippine ants, based on the worker caste (adapted from Bolton 1994)

* Numbers in parentheses refer to the previous couplet in the sequence

**Table:** 

1	Postpetiole absent	2
–	Postpetiole present and petiole and pospetiole nearly equal in size, or postpetiole much larger, in which case, gaster with distinct constriction at insertion with postpetiole	7
2(1)	Sting absent	3
–	Sting present, often conspicuous and functional	4
3(2)	In profile, a circular nozzle, the acidopore, often fringed with hairs, present at apex of gaster; antennal sockets sometimes situated well behind posterior margin of clypeus	Formicinae
–	In profile, acidopore absent; apex of gaster with a transverse slit-like orifice (best seen in apical or lateral view); antennal sockets always abutting the posterior margin of clypeus	Dolichoderinae
4(2)	Petiole broadly attached to gaster, so that petiole has no posterior face in side view	Amblyoponinae
–	Petiole narrowly attached to gaster, so that petiole has a posterior face in side view	5
5(4)	Frontal lobes and clypeus absent or reduced, so that antennal sockets are fully exposed in dorsal view and situated very near or above anterior margin of the head	Proceratiinae
–	Frontal lobes and clypeus well-developed, so that antennal sockets are partially or entirely covered in dorsal view and situated posterior to the anterior margin of the head	6
6(5)	Frontal lobes elongate and roughly parallel, so that frontal carinae do not converge posteriorly; mandibles triangular	Ectatomminae
–	Frontal lobes rounded or bluntly triangular; frontal carinae, when present, appear to converge posteriorly; mandibles variable from linear to triangular	Ponerinae
7(1)	Pronotum and mesonotum separated by a conspicuous, flexible suture, allowing pronotum to move relative to mesonotum	8
–	Pronotum and mesonotum separated by an often inconspicuous, rigid suture, so that pronotum and mesonotum are fused together	9
8(7)	Eyes, present, large; ventral surface of postpetiole at most slightly bulging (*Tetraponera*) (Figs. 37 C, D)	Pseudomyrmecinae
–	Eyes absent; ventral surface of postpetiole usually swollen and bulbous	Leptanillinae
9(7)	Postpetiole barrel-shaped and much larger than petiole; gaster conspicuously constricted at insertion; upper surface of tip of gaster transversely flattened and armed with peg-like teeth	Cerapachyinae
–	Postpetiole globular and nearly equal in size to petiole; gaster with narrow constriction at insertion; upper surface of tip of gaster rounded and unarmed	10
10(9)	With all the following character states: frontal lobes absent; antennal sockets completely exposed; eyes absent; gaster narrowly constricted at insertion with postpetiole (*Aenictus*) (Figs. 9 E, F)	Aenictinae
–	Not as above: either frontal lobes present (much reduced or absent in *Pristomyrmex*), antennal sockets partially or completely concealed (except in *Pristomyrmex*); eyes present, or gaster not constricted at insertion with postpetiole	Myrmicinae

### Keys to the genera of Philippine ants, by subfamily, based on the worker caste (adapted from Bolton 1994)

#### Key to Philippine Amblyoponinae

**Table:** 

1	Frontal lobes large, extending forward beyond anterior clypeal margin; antennal funiculus flattened or compressed (Figs. 24 A, B)	*Myopopone*
–	Frontal lobes never extending beyond anterior clypeal margin; antennal funiculus more or less circular in cross-section	2
2(1)	Mandibles with only 3 teeth, with median tooth smallest; at full closure, little or no gap between mandibles and anterior clypeal margin (Figs. 32 A, B)	*Prionopelta*
–	Mandibles with more than 3 teeth; at full closure, large gap between mandibles and anterior clypeal margin	3
3(2)	Posterior margin of head strongly concave; apex of mandible blunt or rounded (Figs. 25 C, D)	*Mystrium*
–	Posterior margin of head at most weakly concave; apex of mandible acute (Figs. 10 A, B)	*Stigmatomma*

#### Key to Philippine Cerapachyinae

**Table:** 

1	Antennal sockets set close together, separated by a narrow triangular posterior extension of the clypeus; mesotibial spurs present (Figs. 14 C, D)	*Cerapachys*
–	Antennal sockets widely separated, set apart by a wide triangular posterior extension of the clypeus; mesotibial spurs absent (Figs. 35 C, D)	*Simopone*

#### Key to Philippine Dolichoderinae

**Table:** 

1	In profile, petiole overhung by anterior part of gaster and node absent	2
–	In profile, petiole with a conspicuous node, and not overhung by gaster	3
2(1)	In profile, gaster with 4 dorsal segments (tergites), the fifth segment reflexed so that anal pore is ventral, not a termination of gaster (Figs. 36 C, D)	*Tapinoma*
–	In profile, gaster with 5 dorsal segments (tergites) and anal pore is terminal (Figs. 36 E, F)	*Technomyrmex*
3(1)	Palps short and hard to see; in profile, dorsal face of propodeum much shorter than propodeal declivity, giving the mesosoma a compact appearance (Figs. 14 E, F)	*Chronoxenus*
–	Palps long and conspicuous; dorsal face and declivity of propodeum about equal in length so that the mesosoma appears elongated	4
4(3)	Head and mesosoma much longer than broad; legs extremely elongated (Figs. 21 A, B)	*Leptomyrmex*
–	Head roughly triangular and mesosoma not elongated; legs not elongated	5
5(4)	Mesosoma with thick and sculptured integument (Figs. 16 E, F)	*Dolichoderus*
–	Mesosoma with thin integument and generally smooth or shagreened	6
6(5)	In profile, rear face of propodeum concave; in side view, metanotal groove a narrow, distinct notch (Figs. 26 C, D)	*Ochetellus*
–	In profile, rear face of propodeum convex, rarely flat; in side view, metanotal groove a broad impression	*7*
7(6)	In frontal view, posterior margin of head strongly concave and eyes located anterior to midline of of head; petiolar node inclined forward; polymorphic workers (Figs. 30 A, B)	*Philidris*
–	In frontal view, posterior margin of head at most weakly concave, usually flat or convex; eyes posterior to midline of head; petiolar node more or less vertical; monomorphic workers (Figs. 19 E, F)	*Iridomyrmex*

#### Key to Philippine Ectatomminae

**Table:** 

1	In profile, anteroventral margin of pronotum with distinct tooth; hind pretarsal claw with median tooth (Figs. 34 E, F)	*Rhytidoponera*
–	In profile, anteroventral margin of pronotum rounded or bluntly angulate; hind pretarsal claw without median tooth (Figs. 18 E, F)	*Gnamptogenys*

#### Key to Philippine Formicinae

**Table:** 

1	Antenna 8-segmented, folding back below eye; eyes always large (Figs. 18 C, D)	*Gesomyrmex*
–	Antenna with 9- to 12-segmented, folding back above eye; eyes variable in size	2
2(1)	Antenna 9- to 11-segmented	3
–	Antenna 12-segmented	6
3(2)	Propodeum and petiole armed with a pair of spines or teeth (Figs. 20 A, B)	*Lepisiota*
–	Propodeum and petiole without spines or teeth	4
4(3)	Palps very short and extremely difficult to see; eyes minute (Figs. 9 C, D)	*Acropyga*
–	Palps long and prominent; eyes usually well-developed, often large	5
5(4)	Antennal scapes extremely long, extending more than half their length beyond posterior margin of head; erect hairs absent on dorsum of mesosoma (Figs. 11 C, D)	*Anoplolepis*
–	Antennal scape, notably shorter, seldom extending much beyond posterior margin of head; erect hairs sometimes present on dorsum of mesosoma (Figs. 30 C, D)	*Plagiolepis*
6(2)	Mandibles with 10 or more teeth or denticles	7
–	Mandibles with fewer than 10 teeth or denticles	8
7(6)	Mandibles linear and longer than head length, with sharp teeth along most of their length and crossing at apices when closed; eyes enormous (Figs. 25 A, B)	*Myrmoteras*
–	Mandibles triangular and shorter than head length; eyes large but not taking up most of the sides of head (Figs. 27 C, D)	*oecophylla*
8(6)	Antennal sockets almost abutting posterior clypeal margin; ring of hairs present around acidopore	9
–	Antennal sockets well posterior to posterior clypeal margin; ring of hairs often absent around acidopore	15
9(8)	Maxillary palp short and inconspicuous, with 2–4 segments	10
–	Maxillary palp long and conspicuous, with 6 segments	11
10(9)	In side view, mesonotal constriction present; mandibles strongly curved (Figs. 17 C, D)	*Euprenolepis*
–	In side view, mesonotal constriction absent; mandibles not strongly curved (Figs. 33 C, D)	*Pseudolasius*
11(9)	Mesosoma and head without coarse erect hairs (Figs. 27 E, F)	*Overbeckia*
–	Mesosoma and head with coarse erect hairs	12
12(11)	In side view, mesosoma long and slender, with or without constriction of mesonotum	13
–	In side view, mesosoma short and compact, without constriction of mesonotum	14
13(12)	In side view, pronotum only slightly convex; erect setae on head randomly scattered on surface (Figs. 29 A, B)	*Paratrechina*
–	In side view, pronotum convex; erect setae on head form 2 parallel rows (Figs. 31 E, F)	*Prenolepis*
14(12)	Propodeum with 1 pair of erect setae (Figs. 28 C, D)	*Paraparatrechina*
–	Propodeum without a pair of erect setae (Figs. 26 A, B)	*Nylanderia*
15(8)	In side view, metathoracic spiracles forming turbercles that are the highest prominences of the mesosoma (Figs. 17 E, F)	*Forelophilus*
–	In side view, metathoracic spiracles not forming tubercles that are the highest prominences of the mesosoma	16
16(15)	Petiole node lacking teeth or spines; first gastral tergite distinctly less than half total length of gaster (Figs. 12 E, F)	*Camponotus*
–	Petiole node armed with spines, teeth, or denticles; first gastral tergite large, at least half of total length of gaster	17
17(16)	Spines or teeth usually present on pronotum, propodeum, or both; body usually covered with short appressed hairs and some erect hairs (Figs. 31 A, B)	*Polyrhachis*
–	Spines or teeth absent from pronotum and propodeum, often present on petiole; body usually densely covered with long erect hairs (Figs. 17 A, B)	*Echinopla*

#### Key to Philippine Leptanillinae (These ants are tiny, requiring higher magnification for observation of these character states.) The genus *Protanilla*is included because of unconfirmed reports that it has been collected in Palawan Island.

**Table:** 

1	Metanotal groove absent (Fig. 20 C)	*Leptanilla*
–	Metanotal groove present and distinct	2
2	Petiole with a posterior face	*Protanilla*
–	Petiole without a posterior face (Figs. 11 A, B)	*Anomalomyrma*

#### Key to Philippine Myrmicinae

**Table:** 

1	In side view, antennal scrobe present below eye	2
–	In side view, antennal scrobe absent or present above eye	3
2(1)	Antenna 7-segmented; antennal scape triangular, widest near insertion and tapering distally; petiole pedunculate; propodeum armed with thin longitudinal flanges (Figs. 12 A, B)	*Basiceros*
–	Antenna 11-segmented; antennal scape rod-like; petiole sessile; propodeum armed with 2 thick spines (Figs. 13 E, F)	*Cataulacus*
3(1)	Petiole lacking a distinct node	4
–	Petiole with a distinct node	6
4(3)	Petiole transversely flattened; in dorsal view, in dorsal view, gaster roughly heart-shaped; (Figs. 15 A, B)	*Crematogaster*
–	Petiole roughly barrel-shaped; in dorsal view, gaster not heart-shaped	5
5(4)	Side of head with a longitudinal ridge running below the eye, from rear border to mandibular insertion; posterolateral corner of the head not forming a blunt point (Figs. 24 C, D)	*Myrmecina*
–	Side of head lacking a ridge below the eye; posterolateral corner of the head forming a blunt point (Figs. 16 A, B)	*Dilobocondyla*
6(3)	Mandibles bidentate (with 2 teeth); mesosoma without a metanotal groove (Figs. 37 E, F)	*Tyrannomyrmex*
–	Mandibles with more than 2 teeth; mesosoma usually with a metanotal groove	7
7(6)	Petiole and postpetiole with at least some light-colored sponge-like tissue	*8*
–	Petiole and postpetiole never with sponge-like tissue	9
8(7)	Sponge-like tissue only on ventral surface of petiole, postpetiole, and the 4^th^ abdominal segment; antenna 11-segmented (Figs. 15 C, D)	*Dacetinops*
–	Sponge-like tissue on lateral, as well as ventral, surfaces of petiole, postpetiole, and 4^th^ abdominal segment; antenna 4–to 6-segmented (Figs. 36 A, B)	*Strumigenys*
9(7)	Antenna with a distal club of 2 segments	10
–	Antenna with a distal club of 3 or 4 segments, or with an indistinct club	15
10(9)	Antennal scrobe present; head and mesosoma punctuate (Figs. 22 C, D)	*Mayriella*
–	Antennal scrobe absent; head and mesosoma smooth, rugose, or reticulate	11
11(10)	Head and mesosoma sculptured; petiole at most with a short peduncle	12
–	Head and mesosoma smooth between sparse punctures; petiole pedunculate	13
12(11)	Head and mesosoma longitudinally rugose; propodeum unarmed; gaster broadly attached to postpetiole (Figs. 34 A, B)	*Rhopalomastix*
–	Head and mesosoma strongly reticulate; propodeum bidentate; gaster narrowly attached to postpetiole (Figs. 38 E, F)	Unnamed genus PH01
13(11)	Propodeum unarmed; anterior clypeal margin with two angles, or teeth, bearing hairs (Figs. 35 E, F)	*Solenopsis*
–	Propodeum with a pair of spines or teeth; anterior clypeal margin simple	14
14(13)	Clypeus with 2 longitudinal carinae immediately anterior to antennal sockets; workers dimorphic (Figs. 13 C, D)	*Carebara*
–	Clypeus smooth and without longitudinal carinae; workers polymorphic (Figs. 29 E, F)	*Pheidologeton*
15(9)	Antenna with 7–9 segments	16
–	Antenna with 10–12 segments	17
16(15)	Antenna7-segmented; mesosoma strongly reticulate; propodeal spines long; anterior peduncle of petiole as long as petiolar node height (Figs. 24 E, F)	*Myrmicaria*
–	Antenna 9-segmented; mesosoma weakly reticulate and shield-like; propodeal spines short; anterior peduncle shorter than petiolar node height (Figs. 22 E, F)	*Meranoplus*
17(15)	Eyes absent	18
–	Eyes present, even if only one ommatidium	19
18(17)	Petiole and postpetiole with prominent anteroventral processes; integument firm and sclerotized (Figs. 21 C, D)	*Liomyrmex*
–	Petiole and postpetiole lacking anteroventral processes; integument fragile and poorly sclerotized (Figs. 10 C, D)	*Anillomyrma*
19(17)	In dorsal view, postpetiole swollen and at least twice as wide as petiole; antenna 12-segmented (Figs. 13 A, B)	*Cardiocondyla*
–	In dorsal view, postpetiole as wide as, or slightly wider than, petiole; antenna 10–to 12-segmented	20
20(19)	Clypeus with an anterior forked extension overhanging the mandibles; body covered with evenly-spaced clavate hairs (Figs. 12 C, D)	*Calyptomyrmex*
–	Clypeus without an anterior forked extension; body hairs not clavate	21
21(20)	Sides of clypeus immediately anterior to antennal sockets produced into a sharp ridge	22
–	Sides of clypeus immediately anterior to antennal sockets not produced into a sharp ridge	23
22(21)	In frontal view, head roughly heart-shaped, with posterior margin of the head strongly concave; antenna 12-segmented (Figs. 34 C, D)	*Rhoptromyrmex*
–	In frontal view, head roughly rectangular, with posterior margin of the head at most weakly concave; antenna 10–to 12-segmented (Figs. 37 A, B)	*Tetramorium*
23(21)	Anterior clypeal margin with a single median seta extending over the mandibles; propodeum at most with blunt angles; (Figs. 23 C, D)	*Monomorium*
–	Anterior clypeal margin with a pair of setae, or a series of long, strong setae, or hairless; propodeum usually armed with spines	24
24(23)	Antenna 11-segmented	25
–	Antenna 12-segmented	30
25(24)	Antennal sockets completely exposed; anterior clypeal margin denticulate (Figs. 32 C, D)	*Pristomyrmex*
–	Antennal sockets partially or completely covered by frontal lobes; anterior clypeal margin usually simple	26
26(25)	Antennal scrobes deep and narrow, overhung by expanded frontal carinae (Figs. 23 A, B)	*Metapone*
–	Antennal scrobes absent or very shallow	*27*
27(26)	Propodeum unarmed; in profile, petiole lacking peduncle and usually with a large subpetiolar process (Figs. 38 A, B)	*Vollenhovia* (part)
–	Propodeum spinose; in profile, petiole pedunculate	28
28(27)	In profile, propodeal spines curving upward and forward (Figs. 33 E, F)	*Recurvidris*
–	In profile, propodeal spines straight	29
29(28)	Propodeal spines long and sharp; in side view, pronotum convex, much higher than propodeum (Figs. 21 E, F)	*Lophomyrmex*
–	Propodeal spines short and blunt; in side view, pronotum flat, almost level with propodeum (Figs. 18 A, B)	*Gauromyrmex*
30(24)	Antenna gently expanding distally, with a weak to indistinct 4-segmented club; head strongly narrowed posteriorly (Figs. 11 E, F)	*Aphaenogaster*
–	Antenna abruptly expanding distally, with a distinct 3-segmented club; head not narrow posteriorly	31
31(30)	Petiole lacking a peduncle	32
–	Petiole pedunculate	34
32(31)	Propodeum armed with stout spines (curved in dorsal view) (Figs. 35 A, B)	*Romblonella*
–	Propodeum unarmed or at most with denticles	33
33(32)	Petiole with a conspicuous ventral process; mandibles curved mesad and without a stem at insertion (Figs. 38 A, B)	*Vollenhovia* (part)
–	Petiole without a conspicuous ventral process; mandibles linear-triangular and with a stem at insertion (see diagnosis in “Brief Generic Accounts”) (Figs. 39 E, F)	Unnamed genus PH03
34(32)	Anterior clypeal margin overhanging mandibles and armed with blunt denticles or a median rectangular extension; in the major worker, head enormous, obscuring pronotum in dorsal view (Figs. 9 A, B)	*Acanthomyrmex*
–	Anterior clypeal margin not overhanging mandibles and simple; in the major worker, if present, head does not obscure pronotum in dorsal view	35
35(34)	In profile, mesosomal dorsum with a flat or weakly convex outline; petiolar node blocky or roughly rectangular (Figs. 28 E, F)	*Paratopula*
–	In profile, dorsal mesosomal dorsum with a strongly convex outline; petiolar node globular or rounded	36
36(35)	In profile, petiolar spiracle located on node, not on peduncle (see diagnosis in “Brief Generic Accounts”) (Figs. 39 A, B)	*Unnamed genus PH02*
–	In profile, petiolar spiracle located on peduncle	37
37(36)	Mandible with 5 teeth with a long diastema (toothless section) between 3^rd^ and 4^th^ tooth; side of head usually with a sinuate longitudinal groove running from the posterior margin to the mandibular insertion (Figs. 38 C, D)	*Vombisidris*
–	Mandibular dentition not as above; sinuate longitudinal groove at side of head absent	38
38(37)	In profile, petiole with a short anterior peduncle; workers monomorphic (Figs. 22 A, B)	*Lordomyrma*
–	In profile, petiole with a long anterior peduncle; workers dimorphic (Figs. 29 C, D)	*Pheidole*

#### Key to Philippine Ponerinae

**Table:** 

1	Mandibles long and linear, inserted at middle of the anterior margin of head	2
–	Mandibles triangular or linear, inserted at sides of head	3
2(1)	Frons with a conspicuous groove running along the midline; in profile, petiole node produced into a dorsal sharp point (Figs. 26 E, F)	*Odontomachus*
–	Frons simple, at most with shallow striations; in profile, petiole node blunt (Figs. 10 E, F)	*Anochetus*
3(1)	Antennal insertions well separated; hind tibia with two pectinate spurs (Figs. 30 E, F)	*Platythyrea*
–	Antennal insertions closely approximated; hind tibia with one pectinate spur, but may have a smaller simple spur in front of it	4
4(3)	Hind tibia with only one pectinate spur, without a smaller, simple anterior spur	5
–	Hind tibia with two spurs with the smaller spur simple	8
5(4)	Side of mandible near insertion with a small circular pit (Figs. 28 A, B)	*Pachycondyla* (part)
–	Side of mandible near insertion without a pit	6
6(5)	Outer surface of middle tibia and middle and hind tarsi with strong, peg-like teeth; pronotum flat and shelf-like in profile (Figs. 14 A, B)	*Centromyrmex*
–	Outer surface of middle tibia and middle and hind tarsi with hairs, but never with strong, peg-like teeth; pronotum convex in profile	7
7(6)	Subpetiolar process with an oval or circular translucent window, and with a sharp posterior angle (Figs. 31 C, D)	*Ponera*
–	Subpetiolar process without an oval or circular translucent window, and usually blunt or rounded posteriorly (Figs. 19 C, D)	*Hypoponera*
8(4)	Tarsal claws on hind leg either pectinate or with one or more teeth on inner surface	9
–	Tarsal claws on hind leg simple, never with teeth on inner surface	10
9(8)	Ocelli present; mandibles long and forceps-like with a triangular flange beneath (Figs. 19 A, B)	*Harpegnathos*
–	Ocelli absent; mandibles variable but never long and forceps-like with a triangular flange beneath (Figs. 20 D, E)	*Leptogenys*
10(8)	Petiole node with a pair of spines (Figs. 15 C, D)	*Diacamma*
–	Petiole node simple	11
11(10)	Side of pronotum with a pair of large blunt angles; anterior clypeal margin with small blunt teeth or denticles (Figs. 27 A, B)	*Odontoponera*
–	Side of pronotum and anterior clypeal margin simple	12
12(11)	Mandibles, when fully closed, with a large gap between them; eyes situated very near base of mandibles (Figs. 23 E, F)	*Myopias*
–	Mandibles, when fully closed, slightly overlap along the inner margin; eyes situated away from base of mandibles (Figs. 28 A, B)	*Pachycondyla* (part)

#### Key to Philippine Proceratiinae

**Table:** 

1	Fourth abdominal segment straight or slightly curved, so that apex of gaster is directed posteriorly (Figs. 32 E, F)	*Probolomyrmex*
–	Fourth abdominal segment strongly curved, so that apex of gaster is directed anteriorly	2
2(1)	Apical segment of antenna extremely large and bulbous; antennal sockets on shelf protruding over mandibles (Figs. 16 C, D)	*Discothyrea*
–	Apical segment moderately enlarged, but not bulbous; antennal sockets not protruding over mandibles (Figs. 33 A, B)	*Proceratium*

## Glossary of terms

**Abdominal segments** – morphologically, segment I of the ant abdomen is the propodeum (see below), followed by the petiole (see below), which is segment II. The postpetiole (see below), when present, is segment III. Otherwise, the first segment of the gaster (see below) is segment III.

**Acidopore** – at the apex of the gaster (see below), a round, somewhat raised, orifice, often fringed with hairs at the tip; diagnostic of ants belonging to subfamily Formicinae.

**Angle** – a triangular, broad-based, tooth-like extension of the cuticle.

**Antennal club** – describes an antennal funiculus that enlarges apically, to form a distinct club of 2-4 segments.

**Antennal funiculus** – the portion of the antenna distal to the antennal scape; composed of 3-12 segments of varying size.

**Antennal scape** – the elongated basal segment of the antenna.

**Antennal scrobe** – a groove above or below the eye, which accepts the folded antenna.

**Anterior clypeal margin** – the leading edge of the clypeus (see below).

**Bidentate** – armed with 2 teeth (mandibles) or short spines (propodeum).

**Clavate** – refers to hairs that are blunt and club-shaped.

**Clypeus** – the anterior sclerite (see below) of the dorsal head, which consists of narrow lateral portions and a shield-like median portion.

**Coxa** – see Leg segments.

**Declivity** – downward slope, eg. as of the propodeum.

**Dentate** – describes mandibles armed with teeth.

**Denticle** – a small triangular tooth, much reduced in size.

**Denticulate** – armed with denticles.

**Edentate** – describes mandibles without teeth or denticles on the inner margin.

**Epigaeic** – describes ants that live above ground (compare with hypogaeic).

**Filiform** – describes an antennal funiculus with segments of approximately the same size (compare with antennal club).

**Frons** – the region of the head extending from behind the clypeus, and between the eyes to the posterior margin of the head.

**Frontal carina** – (pl. carinae) thin ridges of cuticle on the front of the head, which may form the dorsal margins of the antennal scrobes (see above).

**Frontal lobe** – projection of the frontal carina (see above), which may partially or completely cover the antennal sockets.

**Gaster** – old name for the third main body division of the ant body.

**Head** – the first main body division of the ant body.

**Hypogaeic** – describes ants that live underground (compare with epigaeic).

**Labial palps** – the segmented sensory appendages attached to the labium, found on the anteroventral surface of the head, with a maximum of four segments. These are the inner pair of palps.

**Leg segments** – Each leg consists of the basal coxa, a small trochanter, a usually long and swollen femur, a tibia, anda 5-segmented tarsus, ending in a pair of tarsal claws. Pro-, meso-, and meta- are prefixes that indicate the leg segment of the particular thoracic segment.

**Mandibles** – anterior appendages of the head, with which the ant manipulates its environment. They are variable in shape, dentition, and function, and extremely important in ant taxonomy.

**Maxillary palps** – the segmented sensory appendages attached to the maxillae, found on the anteroventral surface of the head. With a maximum of six segments, they are usually the longer and larger of the palps and are the outer pair of palps.

**Mesosoma** – (= alitrunk) the second main body division of the ant body. Morphologically, it is composed of the three thoracic segments (pro-, meso-, and metathorax) to which is fused the propodeum (see below).

**Mesonotum** – the second tergite (see below) of the mesosoma (see above).

**Mesotibia** – see Leg segments.

**Metanotal groove** – a transverse depression between the mesonotum (see above) and the propodeum (see below).

**Metapleural gland** – an exocrine gland found on the posteroventral side of the mesosoma (see above), just above the metacoxa (see Leg segments). This is one of the defining features of ants, although it has been secondarily lost or reduced in some taxa.

**Node** – a prominent bulge on the dorsal surface of the petiole.

**Ocellus** – (pl. ocelli) a simple eye found on the frons (see above) of the head, usually in reproductives but may also be found in the workers of some taxa.

**Ommatidium** – an individual facet of the compound eye.

**Palp formula** – a standardized method of giving the number of segments of the maxillary and labial palps. The number of maxillary palp (see above) segments is given first, followed by the number of labial palp (see above) segments; thus “PF 6,4” indicates that there are six maxillary palp segments and four labial palp segments.

**Pectinate** – describes tarsal claws or tarsal spurs that are comb-like.

**Pedunculate** – bearing a stem-like projection, or peduncle, that is anterior to the petiolar node (compare with sessile and sub-sessile).

**Petiole** – the isolated segment separating the mesosoma (see above) and the gaster (see above). This is one of the defining features of ants. Morphologically, it is the second segment of the abdomen.

**Postpetiole** – the second isolated and reduced segment separating the mesosoma (see above) and the gaster (see above), in ants with a 2-segmented waist. Morphologically, it is the third abdominal segment.

**Pronotum** – the first tergite (see below) of the mesosoma (see above).

**Propodeum** – (= epinotum) the dorsal posterior plate of the mesosoma (see above). Morphologically, it is the first segment of the abdomen, fused to the thorax. It may have specializations such as spines, teeth, or lobes.

**Punctate** – describes surface sculpturing composed of round pits which may be shallow or deep.

**Reticulate** – describes surface sculpturing composed of an irregular network of thin ridges of cuticle.

**Rugose** – describes surface sculpturing of raised ridges without cross-ridges.

**Sclerite** – a hardened or stiffened plate of the integument.

**Sculpturing** – features on the body surface which may be pits, grooves, striae, ridges, punctures, or a combination.

**Sessile** – describes a petiole without a peduncle, such that the petiolar node is very close to the propodeum (compare with pedunculate).

**Shagreened** – describes body surface that has a dull, light-absorbing quality.

**Spinose** – armed with spines.

**Sternite** – the lower or ventral sclerite (see above) of a segment.

**Striate** – describes sculpturing composed of shallow, parallel grooves or lines.

**Tergite** – the upper or dorsal sclerite (see above) of a segment.

## Figures and Tables

**Table:** 

**Species**	**Distribution**	**Notes**
*Acanthomyrmex mindanao* Moffett, 1986	Md	
*Acropyga acutiventris* Roger, 1862	Md	
*Acropyga nipponensis* Terayama, 1985	Pl	
*Acropyga pallida* (Donisthorpe, 1938)	Lz	A/G – new record
*Aenictus alticola* Wheeler & Chapman (in Wheeler, 1930c)	Lz	
*Aenictus bakeri* Menozzi, 1925	Md	
*Aenictus camposi* Wheeler & Chapman, 1925	Lz, Ng	
*Aenictus carolianus* Zettel & Sorger, 2010b	Cb	
*Aenictus ceylonicus* (Mayr, 1866a)	Lz, Ng	
*Aenictus gracilis* Emery, 1893b	Lz, Md, Mr, Ng	
*Aenictus laeviceps* (F. Smith, 1857)	Ly, Lz, Md, Ng	
*Aenictus luzoni* Wheeler & Chapman, 1925	Lz, Ng	
*Aenictus nesiotis* Wheeler & Chapman (in Wheeler, 1930c)	Lz, Ng, Pl	
*Aenictus pangantihoni* Zettel & Sorger, 2010b	Cm	
*Aenictus philippinensis* Chapman, 1963	Ng	
*Aenictus piercei* Wheeler & Chapman (in Wheeler, 1930c)	Md, Ng	
*Aenictus powersi* Wheeler & Chapman (in Wheeler, 1930c)	Ng	
*Aenictus rabori* Chapman, 1963	Ng	
*Aenictus reyesi* Chapman, 1963	Ng	
*Anillomyrma decamera* (Emery, 1901b)	Lz	
*Anochetus brevis* Brown, 1978	Md	
*Anochetus cato* Forel, 1901		
*Anochetus graeffei* Mayr, 1870	Lz, Ms, Ng, Pl	
*Anochetus incultus* Brown, 1978	Lz	
*Anochetus isolatus* Mann, 1919	Lz, Ng	
*Anochetus leyticus* Zettel, 2012	Ly	
*Anochetus modicus* Brown, 1978	Lz, Ng	
*Anochetus pangantihoni* Zettel, 2012	Ly	
*Anochetus princeps* Emery, 1884	Lz	
*Anochetus ruginotis* Stitz, 1925	Lz, Md	
*Anochetus schoedli* Zettel, 2012	Lz	
*Anochetus turneri* Forel, 1900b	Lz	
*Anochetus werneri* Zettel, 2012	Md	
*Anomalomyrma helenae* Borowiec, Schulz, Alpert & Baňař, 2011	Pl	
*Anoplolepis gracilipes* (F. Smith, 1857)	W	
*Basiceros chapmani* (Taylor, 1990b)	Lz	
*Basiceros philippinum* (Brown & Kempf, 1960)	Lz, Ng	
*Basiceros procerum* (Emery, 1897a)	W	
*Calyptomyrmex beccarii* Emery, 1887b	Lz, Ng	
*Calyptomyrmex loweryi* Shattuck, 2011	Lz, Md	
*Camponotus albocinctus* (Ashmead, 1905)	Lz	
*Camponotus barbatus* Roger, 1863		
*Camponotus barbosus* Baroni Urbani, 1971	Lz, Md	
*Camponotus bellus leucodiscus* Wheeler, 1919a	Lz	
*Camponotus carin* Emery, 1889	Lz	
*Camponotus castanicola* Donisthorpe, 1943b	Lz, Md	
*Camponotus cinerascens* (Fabricius, 1787)		
*Camponotus compressus* (Fabricius, 1787)	Pl	
*Camponotus corallinus* (Roger, 1863)	Lz, Md	
*Camponotus gigas* (Latreille, 1802)	Lz	
*Camponotus horrens* Forel, 1910	Lz	
*Camponotus irritans pallidus* (F. Smith, 1857)	Lz	
*Camponotus japonicus* Mayr, 1866b	Md	
*Camponotus karawaiewi* Menozzi, 1926	Md	
*Camponotus leonardi* Emery, 1889	Lz, Ng	
*Camponotus leonardi gracilentus* Stitz, 1925	Lz	
*Camponotus maculatus foveolatus* Stitz, 1925		
*Camponotus maculatus subnudus* Stitz, 1925	Lz, Pl	
*Camponotus nicobarensis monticola* Bingham, 1903	Lz	
*Camponotus nigricans* Roger, 1863	Jo, Lz	
*Camponotus platypus* Roger, 1863	Lz, Md	
*Camponotus pressipes* Emery, 1893c	Md	
*Camponotus quadrisectus* (F. Smith, 1858)	Md, Pl	
*Camponotus reticulatus jagori* Stitz, 1925	Lz	
*Camponotus rothneyi makilingi* Viehmeyer, 1916b	Lz	
*Camponotus rufifemur obscurus* Stitz, 1925	Lz	
*Camponotus solenobius* Menozzi, 1926	Md	
*Camponotus trietericus* Menozzi, 1926	Lz	
*Camponotus variegatus crassinodis* Forel, 1892a	Ng	
*Camponotus vitreus* (F. Smith, 1860b)	W	
*Cardiocondyla kagutsuchi* Terayama, 1999	By, Lz	
*Cardiocondyla mauritanica* (Forel, 1890)	Lz	
*Cardiocondyla sima* Wheeler, 1935b	Lz, Md	
*Cardiocondyla tjibodana* Karavaiev, 1935	By, Lz	
*Cardiocondyla wroughtonii* (Forel, 1890)	Lz	
*Carebara alperti* Fernandez, 2010	Ng	
*Cataulacus catuvolcus* Bolton, 1974	Lz, Rm	
*Cataulacus chapmani* Bolton, 1974	Lz, Ng	
*Cataulacus setosus* F. Smith, 1860b	Md	
*Centromyrmex feae* (Emery, 1889)	Bs, Lz	
*Cerapachys bakeri* (Wheeler & Chapman, 1925)	Bs	
*Cerapachys biroi* Forel, 1907		
*Cerapachys crawleyi* Wheeler, 1924b	Lz, Ng	
*Cerapachys jacobsoni* Forel, 1912a	Ng	
*Cerapachys longitarsus* (Mayr, 1879)	Lz	
*Cerapachys luzuriagae* (Wheeler & Chapman, 1925)	Ng	
*Cerapachys muiri* Wheeler & Chapman, 1925	Lz, Md	
*Cerapachys pruinosus* Brown, 1975	Ng	
*Cerapachys rufithorax* Wheeler & Chapman, 1925	Lz, Ng	
*Cerapachys sulcinodis* Emery, 1889	Lz, Ng	
*Cerapachys suscitatus* (Viehmeyer, 1913)	Ng	
*Crematogaster ampullaris* F. Smith, 1861	Ly, Lz	
*Crematogaster bakeri* Menozzi, 1925	Md	
*Crematogaster bicolor imbellis* Emery, 1893c	Lz, Ng	
*Crematogaster brunnea ruginota* Santschi, 1928a		
*Crematogaster crassicornis* Emery, 1893c	Lz, Md	
*Crematogaster cylindriceps* Wheeler, 1927		
*Crematogaster difformis* F. Smith, 1857	Lz	
*Crematogaster inflata* F. Smith, 1857	Lz	
*Crematogaster longiclava* Emery, 1893c	Lz	
*Crematogaster modiglianii* Emery, 1900b	Md, Ng	
*Crematogaster ochracea* Mayr, 1862	Lz, Ng	
*Crematogaster onusta* Stitz, 1925	Pl, SC	
*Crematogaster rogenhoferi* Mayr, 1879	Lz	
*Crematogaster semperi* Emery, 1893c	Lz	
*Crematogaster simoni* Emery, 1893c	Lz	
*Crematogaster vitalisi* Menozzi, 1925	Md	
*Dacetinops cirrosus* Taylor, 1985	Ly	HZW – new record
*Diacamma baguiense* Wheeler & Chapman, 1925	Lz	
*Diacamma palawanicum concentricum* Wheeler & Chapman, 1925	Pl	
*Diacamma palawanicum* Emery, 1900b	Pl	
*Diacamma panayense* Wheeler & Chapman, 1925	Pn	
*Diacamma rugosum* (Le Guillou, 1842)	W	
*Diacamma rugosum viridipurpureum* Emery, 1893c	Lz	
*Diacamma sericeiventre* Stitz, 1925	Pl	
*Dilobocondyla chapmani rufobrunnea* Wheeler, 1935b	Ng	
*Dilobocondyla chapmani* Wheeler, 1924a	Ng	
*Discothyrea bryanti* (Wheeler, 1917)	Lz	
*Discothyrea clavicornis* Emery, 1897a	Lz	
*Dolichoderus affinis* Emery, 1889		
*Dolichoderus patens pubiventris* Emery, 1900b	Pl	
*Dolichoderus thoracicus* (F. Smith, 1860a)	W	
*Echinopla pallipes* F. Smith, 1857	Lz	
*Echinopla pseudostriata* Donisthorpe, 1943a	Lz	
*Echinopla striata aciculata* (F. Smith, 1858)		
*Echinopla striata* F. Smith, 1857		
*Echinopla vermiculata* Emery, 1898	Lz	
*Euprenolepis negrosensis* (Wheeler, 1930b)	Ng	
*Forelophilus philippinensis* Zettel & Zimmerman, 2007	Lz, Md	
*Forelophilus stefanschoedli* Zettel & Zimmerman, 2007	Ly, Lz, Md	
*Gauromyrmex acanthinus* (Karavaiev, 1935)	MCZ – new record	
*Gesomyrmex luzonensis* (Wheeler, 1916)	Lz, Ng	
*Gnamptogenys binghamii* (Forel, 1900d)	Lz, Ng	
*Gnamptogenys bulbopila* Lattke, 2004	Md	
*Gnamptogenys chapmani* Brown, 1958	Lz, Ng	
*Gnamptogenys costata* (Emery, 1889)	Lz, Md	
*Gnamptogenys cribrata* (Emery, 1900a)	Lz, Ng	
*Gnamptogenys fistulosa* Lattke, 2004	Lz	
*Gnamptogenys laevior* (Forel, 1905)	Lz	
*Gnamptogenys leiolabia* Lattke, 2004	Ng	
*Gnamptogenys luzonensis* (Wheeler, 1929d)	Lz, Ng	
*Gnamptogenys menadensis* (Mayr, 1887)	Lz	
*Gnamptogenys posteropsis* (Gregg, 1951)	Lz, Md, Ng	
*Harpegnathos empesoi* Chapman, 1963	Md	
*Harpegnathos macgregori* Wheeler & Chapman, 1925	Bi	
*Harpegnathos medioniger* Donisthorpe, 1942	Lz	
*Harpegnathos venator chapmani* Donisthorpe, 1937	Lz	
*Hypoponera confinis javana* (Forel, 1905)	Lz, Ng	
*Hypoponera opaciceps* (Mayr, 1887)		
*Hypoponera pruinosa* (Emery, 1900a)	Lz	
*Hypoponera punctatissima* (Roger, 1859)	Lz	
*Hypoponera ragusai* (Emery, 1894)	Lz	
*Hypoponera sabronae* (Donisthorpe, 1941b)		
*Iridomyrmex anceps* (Roger, 1863)	Mq	
*Iridomyrmex angusticeps* Forel, 1901	Md	
*Lepisiota aurea punctaticeps* Wheeler, 1935b	Lz, Ng	
*Lepisiota chapmani* Wheeler, 1935b	Ng	
*Leptanilla astylina* Petersen, 1968	Pl	
*Leptogenys chinensis* (Mayr, 1870)	Ng	
*Leptogenys diminuta* (F. Smith, 1857)	Lz, Md, Ng, Pl	
*Leptogenys falcigera* Roger, 1861	Ng	
*Leptogenys iridescens* (F. Smith, 1857)	Pl	
*Leptogenys maxillosa* (F. Smith, 1858)	Pn	
*Leptogenys mutabilis* (F. Smith, 1861)	Pl	
*Leptogenys peuqueti* (Andre, 1887)	Lz, Ng	
*Leptogenys pruinosa* Forel, 1900d	Lz, Ng	
*Leptogenys punctiventris* (Mayr, 1879)	Lz, Ng	
*Leptogenys varicosa* (Stitz, 1925)	Pl	
*Leptogenys watsoni* Forel, 1900d	Lz, Ng, Pn	
*Leptomyrmex fragilis* (F. Smith, 1859)	Md	BPBM – new record
*Liomyrmex gestroi* (Emery, 1887b)	W	
*Lophomyrmex bedoti* Emery, 1893b	Pl	
*Lordomyrma diwata* Taylor, 2012	Lz	
*Lordomyrma emarginata* Taylor, 2012	Lz	
*Lordomyrma idianale* Taylor, 2012	Lz	
*Lordomyrma limatula* Taylor, 2012	Ly	
*Mayriella transfuga* Baroni Urbani, 1977a	Lz	A/G – new record
*Meranoplus biliran* Schödl, 1998	Bi	
*Metapone bakeri* Wheeler, 1916	Lz	
*Metapone gracilis* Wheeler, 1935b	Md	
*Monomorium banksi* Forel, 1910	Ng	
*Monomorium destructor* (Jerdon, 1851)	Lz	
*Monomorium floricola* (Jerdon, 1851)	Cm, Lz, Md, Tb	
*Monomorium orientale* Mayr, 1879		
*Monomorium pharaonis* (Linnaeus, 1758)	W	
*Myopias bidens polita* (Stitz, 1925)	Ng, Pl	
*Myopias breviloba* (Wheeler, 1919a)	Bs	
*Myopias lobosa* Willey & Brown, 1983	Lz	
*Myopias modiglianii* (Emery 1900b)	Ng	
*Myopias philippinensis* (Menozzi, 1925)	Lz, Md	
*Myopopone castanea* (F. Smith 1860b)	Ly, Lz, Md, Pl	
*Myrmicaria aphidicola* Calilung, 2000	Md	
*Myrmicaria brunnea subcarinata* (F. Smith, 1857)	W	
*Myrmoteras glabrum* Zettel & Sorger, 2011	Cm	
*Myrmoteras insulcatum* Moffett, 1985	Lz, Ng	
*Myrmoteras mcarthuri* Zettel & Sorger, 2011	Ly	
*Myrmoteras williamsi* Wheeler, 1919a	Lz, Ng	
*Mystrium camillae* Emery, 1889	Lz, Pl	
*Noonilla copiosa* Petersen, 1968	Pl	
*Ochetellus glaber* (Mayr, 1862)	Lz, Ng	MCZ – new record
*Odontomachus alius* Sorger & Zettel, 2011	Bh, Bi, Cb, Ct, Ly, Sm	
*Odontomachus banksi* Forel, 1910	Cb, Lz	
*Odontomachus infandus* F. Smith, 1858	Lz, Mr, Ng, Rm	
*Odontomachus malignus* F. Smith, 1859	Ba, Bh, Jo, Tw	
*Odontomachus papuanus* Emery, 1887b	Ng, Pl, Pn	
*Odontomachus philippinus* Emery, 1893b	Ng, Pn, Sq	
*Odontomachus rixosus* F. Smith, 1857	Md, Pl	
*Odontomachus saevissimus* F. Smith, 1858	Lz, Rm	
*Odontomachus schoedli* Sorger & Zettel, 2011	Lz	
*Odontomachus scifictus* Sorger & Zettel, 2011	Cm	
*Odontomachus simillimus* F. Smith, 1858	W	
*Odontoponera denticulata* Wheeler & Chapman, 1925	W	
*Oecophylla smaragdina* (Fabricius, 1775)	W	
*Overbeckia subclavata* Viehmeyer, 1916a	MCZ – new record	
*Pachycondyla annamita* (Andre, 1892)	Lz	
*Pachycondyla chinensis* (Emery, 1895b)	Lz	
*Pachycondyla claudata* (Menozzi, 1926)	Lz, Sm	
*Pachycondyla croceicornis* (Emery, 1900a)		
*Pachycondyla darwinii indica* (Emery, 1899)	Lz, Ng	
*Pachycondyla glabripes* (Emery, 1893c)	Md	
*Pachycondyla javana* (Mayr 1867)		
*Pachycondyla leeuwenhoeki* (Forel, 1886a)	Ng	MCZ – new record
*Pachycondyla luteipes* (Mayr, 1862)	Lz, Ng, Pl	
*Pachycondyla melancholica* F. Smith, 1865		
*Pachycondyla myropola* (Menozzi, 1925)	Lz	
*Pachycondyla obscurans* (Walker, 1859)	Lz	
*Pachycondyla stigma* (Fabricius, 1804)	Bs, Lz, Md, Mr	
*Pachycondyla testacea* (Emery, 1893a)	Lz	A/G – new record
*Pachycondyla tridentata* F. Smith, 1858	Jo, Tw	
*Pachycondyla williamsi* (Wheeler & Chapman, 1925)	Lz	
*Paraparatrechina iridescens* (Donisthorpe, 1942)	Lz, Pl	
*Paratopula ceylonica* (Emery, 1901)		
*Paratopula macta* Bolton, 1988	Lz	
*Paratopula sumatrensis* (Forel, 1913)	Lz	
*Paratrechina longicornis* (Latreille, 1802)	W	
*Pheidole aglae* Forel, 1913	Lz	
*Pheidole aristotelis* Forel, 1911a	Ng, Pl	
*Pheidole cariniceps* Eguchi, 2001a	Lz	A/G – new record
*Pheidole clypeocornis* Eguchi, 2001a	Lz	
*Pheidole fantasia* Chapman, 1963	Lz, Ng	
*Pheidole fervens* F. Smith, 1858	Lz, Ng	
*Pheidole guineensis* Fabricius, 1793	Pl	
*Pheidole hortensis* Forel, 1913	Lz, Sm	
*Pheidole inscrobiculata* Viehmeyer, 1916a	Lz	
*Pheidole jacobsoni* Forel, 1911b	Lz, Md, Sm	
*Pheidole longipes* (F. Smith, 1857)	Md	
*Pheidole maculifrons* Wheeler, 1929d	Lz	
*Pheidole makilingi* Viehmeyer, 1916b	Lz	
*Pheidole megacephala* (Fabricius, 1793)		
*Pheidole plagiaria palawanica* Stitz, 1925	Ly, Pl	
*Pheidole quadricuspis* Emery, 1900a	Lz	
*Pheidole sarawakana* Forel, 1911a	Lz	
*Pheidole sauberi* Forel, 1905	Lz	
*Pheidole sayapensis* Eguchi, 2001a	Lz	
*Pheidole simoni* Emery, 1893c	Lz	
*Pheidole tjibodana* Forel, 1905	Lz	
*Pheidologeton affinis* (Jerdon, 1851)	Md, Pl	
*Pheidologeton diversus* (Jerdon, 1851)	Lz	
*Pheidologeton diversus macgregori* Wheeler, 1929	Sm	
*Pheidologeton diversus philippinus* Wheeler, 1929	Cb, Lz, Ng	
*Pheidologeton diversus tenuirugosus* Wheeler, 1929	Lz	
*Pheidologeton diversus williamsi* Wheeler, 1929	Lz, Md	
*Pheidologeton maccus* Wheeler, 1929d	Lz, Sm	
*Pheidologeton pygmaeus albipes* Emery, 1893c	Lz	
*Pheidologeton silvestrii* Wheeler, 1929d	Lz	
*Philidris myrmecodiae* (Emery, 1887a)	Lz	
*Platythyrea bidentata* Brown, 1975	Ng	
*Platythyrea inermis* Forel, 1910	Lz, Ng, Sm	
*Platythyrea parallela* (F. Smith, 1859)	Lz, Md, Ng, Rm	
*Platythyrea quadridenta* Donisthorpe, 1941b	Pl	
*Polyrhachis abdominalis* F. Smith, 1858	Lz, Pl	
*Polyrhachis aculeata* Mayr, 1879	Ng	
*Polyrhachis aequalis* Forel, 1910	Lz, Ng, Pl	
*Polyrhachis arcuata* (Le Guillou, 1842)		
*Polyrhachis armata* (Le Guillou, 1842)	Lz, Md, Ng, Pl	
*Polyrhachis armata defensa* F. Smith, 1857	Pl	
*Polyrhachis aureovestita* Donisthorpe, 1937b	Md	
*Polyrhachis baca* Sorger & Zettel, 2010	Lz	
*Polyrhachis bakeri* Viehmeyer, 1916b	Lz	
*Polyrhachis banghaasi* Viehmeyer, 1922		
*Polyrhachis bellicosa* F. Smith, 1859	Lz, Ng	
*Polyrhachis bicolor* F. Smith, 1858	Lz, Pl	
*Polyrhachis bicolor aurinasis* Forel, 1901b		
*Polyrhachis bicolor exflavicornis* Baltazar, 1966		
*Polyrhachis bicolor fumata* Stitz, 1925	Lz	
*Polyrhachis bihamata* (Drury, 1773)	Lz, Md, Ng	
*Polyrhachis calypso* Forel, 1911c	Tw	
*Polyrhachis carbonaria* F. Smith, 1857	Pl	
*Polyrhachis chapmani* Kohout, 2006b	Ng	
*Polyrhachis creusa* Emery, 1897b	Ng	
*Polyrhachis cryptoceroides* Emery, 1887a	Lz	
*Polyrhachis cyaniventris* F. Smith, 1858	Cb, Ct, Ly, Lz, Mr, Sm	
*Polyrhachis diana* Wheeler, 1909	Md	
*Polyrhachis dives* F. Smith, 1857	Lz, Pl	
*Polyrhachis empesoi* Kohout, 2006b	Ng	
*Polyrhachis esuriens* Emery, 1897b	Ng	
*Polyrhachis etheli* Chapman, 1963	Ng	
*Polyrhachis exotica* Kohout, 1987	Md	
*Polyrhachis follicula* Menozzi, 1926	Lz	
*Polyrhachis fruhstorferi* Emery, 1898		
*Polyrhachis hector* F. Smith, 1857	Ng	
*Polyrhachis hippomanes boettcheri* Stitz, 1925	Lz, Md, Ng	
*Polyrhachis ignota* Kohout, 1987	Lz	
*Polyrhachis illaudata* Walker, 1859	Lz, Pl	
*Polyrhachis inermis* F. Smith, 1858	Ng, Pl	
*Polyrhachis javanica* Mayr, 1867		
*Polyrhachis lama* Kohout, 1994	Lz	
*Polyrhachis magnifica* Menozzi, 1926	Lz, Ms, Ng, Sy, Tb	
*Polyrhachis manni* Kohout, 2008		
*Polyrhachis marginata* F. Smith, 1859		
*Polyrhachis mindanaensis* Emery, 1923	Lz, Md	
*Polyrhachis mitrata* Menozzi, 1932	Md	
*Polyrhachis mucronata* F. Smith, 1859		
*Polyrhachis muelleri* Forel, 1893a	Md, Ng	
*Polyrhachis murina* Emery, 1893b	Jo, Lz, Sm	
*Polyrhachis neptunus* F. Smith, 1865	Lz	
*Polyrhachis nigripes* Emery, 1897b	Lz	
*Polyrhachis nigropilosa* Mayr, 1872	Ng, Pl	
*Polyrhachis nigropilosa polluta* Menozzi, 1926	Md	
*Polyrhachis noesaensis* Forel, 1915	Ng, Pl, Si	
*Polyrhachis obesior* Viehmeyer, 1916a		
*Polyrhachis oedocantha* Wheeler, 1919a	Ng	
*Polyrhachis olybria* Forel, 1912b	Lz, Md, Ng	
*Polyrhachis orpheus* Forel, 1911b		
*Polyrhachis osiris* Bolton, 1975	Lz, Ng	
*Polyrhachis parabiotica* Chapman, 1963	Lz, Ng	
*Polyrhachis pellita* Menozzi, 1922	Lz, Ng, Sm	
*Polyrhachis phalerata* Menozzi, 1926	Lz	
*Polyrhachis philippinensis* F. Smith, 1858	Lz	
*Polyrhachis pirata* Sorger and Zettel, 2009	Lz, Mr, Sm	
*Polyrhachis pressa* Mayr, 1862	Md	
*Polyrhachis pubescens* Mayr, 1879	Lz	
*Polyrhachis rixosa* F. Smith, 1858	Ng	
*Polyrhachis rufipes* F. Smith, 1858	Lz	
*Polyrhachis saevissima* F. Smith, 1860a	Lz, Md, Ng, Pl	
*Polyrhachis saevissima argentea* Mayr, 1862	Lz, Pl	
*Polyrhachis saigonensis* Forel, 1886b	Lz	
*Polyrhachis scabra* Kohout, 1987	Md	
*Polyrhachis schang* Forel, 1879	Ly, Sm	
*Polyrhachis sculpturata* F. Smith, 1860a	Lz, Pl	
*Polyrhachis sculpturata javaniana* Santschi, 1928b	Pl	
*Polyrhachis semiinermis* Donisthorpe, 1941c	Lz, Md	
*Polyrhachis sexspinosa* (Latreille, 1802)	Sm	
*Polyrhachis solivaga* Menozzi, 1926	Lz	
*Polyrhachis striata* Mayr, 1862	Pl	
*Polyrhachis thrinax* Roger, 1863	Lz	
*Polyrhachis thrinax castanea* Stitz, 1925	Lz	
*Polyrhachis thrinax overbecki* Dorow, 1995		
*Polyrhachis tragos* Stitz, 1925	Lz, Md	
*Polyrhachis tubifex* Karavaiev, 1926	Lz	
*Polyrhachis venus* Forel, 1893a	Lz, Md	
*Polyrhachis villipes* F. Smith, 1857	Md	
*Polyrhachis vindex* F. Smith, 1857	Lz, Ng	
*Polyrhachis ypsilon* Emery, 1887a	Lz, Ng	
*Polyrhachis zopyra* F. Smith, 1861	Lz, Ng	
*Ponera chapmani* Taylor, 1967	Lz, Md, Sm	
*Ponera oreas* (Wheeler, 1933)	Ng	
*Prionopelta kraepelini* Forel, 1905	Lz, Ng, Sm, Pl	UPLB
*Pristomyrmex bicolor* Emery, 1900b	Lz, Pl	
*Pristomyrmex brevispinosus* Emery, 1887b	Md	
*Pristomyrmex cebuensis* Zettel, 2007	Cb	
*Pristomyrmex collinus* Wang, 2003	Lz, Md, Ng, Pn, Tb	
*Pristomyrmex costatus* Wang, 2003	Md	
*Pristomyrmex curvulus* Wang, 2003	Ng	
*Pristomyrmex distinguendus* Zettel, 2006	Ly, Lz	
*Pristomyrmex divisus* Wang, 2003	Ng	
*Pristomyrmex flatus* Wang, 2003	Lz	
*Pristomyrmex hirsutus* Wang, 2003	Md	
*Pristomyrmex levigatus* Emery, 1897a	Lz	
*Pristomyrmex longispinus* Wang, 2003	Ng	
*Pristomyrmex picteti* Emery, 1893b	Ly, Lz, Md, Ng, Pl	
*Pristomyrmex punctatus* (F. Smith, 1860b)	Ly, Md, Mq, Mr, Pc, Sm	
*Pristomyrmex quadridens* Emery, 1897a	Ly	
*Pristomyrmex rugosus* Zettel, 2006	Ly	
*Pristomyrmex schoedli* Zettel, 2006	Ly	
*Pristomyrmex simplex* Wang, 2003	Lz	
*Probolomyrmex dammermani* Wheeler, 1928	Ng	MCZ – new record
*Proceratium papuanum* Emery, 1897a	Lz	A/G – new record
*Pseudolasius typhlops* Wheeler, 1935b	Lz, Pl	
*Recurvidris nigrans* Zettel, 2008	Ng	
*Rhoptromyrmex wroughtonii* Forel, 1902a	Lz	MCZ, A/G
*Rhytidoponera araneoides* (Le Guillou, 1842)	Ng	BPBM– new record
*Rhytidoponera croesus* Emery 1901a	Lz	
*Romblonella opaca* (F. Smith, 1861)	Pl, Rm, Rr	
*Simopone chapmani* Taylor, 1966	Ng	
*Solenopsis geminata* (Fabricius, 1804)	W	
*Stigmatomma luzonicum* Wheeler & Chapman, 1925	Lz	
*Stigmatomma reclinatum* (Mayr, 1879)	Lz, Pl	
*Stigmatomma rothneyi* Forel, 1900c	Lz	
*Strumigenys arrogantia* Bolton, 2000	Lz, Pl	
*Strumigenys chapmani* Brown, 1954	Lz, Ng	
*Strumigenys dohertyi* Emery, 1897a	Lz	
*Strumigenys emmae* (Emery, 1890)	Lz	
*Strumigenys esrossi* Brown, 1957	Lz	
*Strumigenys godeffroyi geococci* Calilung, 2000	Lz	
*Strumigenys godeffroyi* Mayr, 1866a	Lz, Md, Pl	
*Strumigenys imantodes* Bolton, 2000	Lz	
*Strumigenys indigatrix*Wheeler, 1919a	Pl	
*Strumigenys inhonesta* Bolton, 2000	Lz	
*Strumigenys jacobsoni* Menozzi, 1939	Pl	
*Strumigenys juliae* Forel, 1905	Pl	
*Strumigenys karawajewi* Brown, 1948	Lz	
*Strumigenys koningsbergeri* Forel, 1905	Lz, Pl	
*Strumigenys lewisi* Cameron, 1886	Lz, Plw	A/G – new record
*Strumigenys mirifica* Bolton, 2000	Lz	
*Strumigenys mitis* (Brown, 2000)	Lz	
*Strumigenys mododonta* Bolton, 2000	Lz	
*Strumigenys morphica* Bolton, 2000	Lz	A/G – new record
*Strumigenys pedunculata* (Brown, 1953)	Lz, Ng	
*Strumigenys perplexa* (F. Smith, 1876)	Lz	A/G – new record
*Strumigenys phytibia* Brown, 1957		
*Strumigenys rantan* Bolton, 2000	Md	
*Strumigenys rogeri* Emery, 1890		
*Strumigenys scylla* (Bolton, 2000)	Lz	
*Strumigenys serradens* (Bolton, 2000)	Lz	
*Strumigenys signeae* Forel, 1905	Pl	
*Strumigenys synchysis* Bolton, 2000	Lz	A/G – new record
*Strumigenys szalayi* Emery, 1897a	Lz, Md, Mr, Ng	
*Strumigenys tenitecta* Bolton, 2000	Lz	
*Strumigenys uichancoi* Brown, 1957		
*Tapinoma emeryi* (Ashmead, 1905)	Lz	SI
*Tapinoma melanocephalum* (Fabricius, 1793)	W	
*Tapinoma philippinense* Donisthorpe, 1942	Lz	
*Tapinoma williamsi* (Wheeler, 1935b)	Lz	
*Technomyrmex albipes* (F. Smith, 1861)	Lz, Mr	
*Technomyrmex difficilis* Forel, 1892b	By, Lz, Md	
*Technomyrmex elatior* Forel, 1902b	Lz, Md	
*Technomyrmex grandis* Emery, 1887a	Bh, Ly, Md, Ng	
*Technomyrmex kraepelini* Forel, 1905	Lz	
*Technomyrmex pratensis* (F. Smith, 1860b)	Md	
*Technomyrmex sundaicus* (Emery, 1900b)	Lz, Md, Sy	
*Technomyrmex textor* Forel, 1909	Lz, Pl	
*Technomyrmex vitiensis* Mann, 1921	Pl	
*Technomyrmex wheeleri* (Emery, 1913a)	Ly, Lz, Rm	
*Tetramorium aspersum* (F. Smith, 1865)	Lz, Md, Ng	
*Tetramorium bicarinatum* (Nylander, 1846)	W	
*Tetramorium chapmani* Bolton, 1977	Ng	
*Tetramorium cynicum* Bolton, 1977	Ng	
*Tetramorium eleates* Forel, 1913	Lz	
*Tetramorium guineense* (Bernard, 1953)	Lz, Ng, Pl, Sm	
*Tetramorium insolens* (F. Smith, 1861)	Ng	
*Tetramorium katypum* (Bolton, 1976)	Cm, Lz, Md	
*Tetramorium kheperra* (Bolton, 1976)	Md	
*Tetramorium khnum* Bolton, 1977	Ng	
*Tetramorium kraepelini* Forel, 1905	Ng	
*Tetramorium lanuginosum* Mayr, 1870	Mr, Ng	
*Tetramorium laparum* Bolton, 1977	Ng	
*Tetramorium manobo* (Calilung, 2000)	Md	
*Tetramorium obtusidens* Viehmeyer, 1916a		
*Tetramorium pacificum* Mayr, 1870	Ly, Lz, Md, Ng, Rm	
*Tetramorium parvispinum* (Emery, 1893b)	Lz	
*Tetramorium rinatum* Bolton, 1977	Ng	
*Tetramorium simillimum* (F. Smith, 1851)	Ng	
*Tetramorium smithi* Mayr, 1879		
*Tetramorium tonganum* Mayr, 1870		
*Tetramorium tortuosum* Roger, 1863		
*Tetramorium walshi* Forel 1890		
*Tetramorium zypidum* Bolton, 1977	Ng	
*Tetraponera allaborans* (Walker, 1859)	W	
*Tetraponera attenuata* F. Smith, 1877	Pl	
*Tetraponera difficilis* (Emery, 1900b)	Ly, Lz, Ng, Pl, Sm	
*Tetraponera extenuata* Ward, 2001	Lz, Md	
*Tetraponera inversinodis* Ward, 2001	Pl	
*Tetraponera modesta* (F. Smith, 1860b)	Ng, Pl, Rm, Sy, Tb	
*Tetraponera nigra* (Jerdon, 1851)	Pl	
*Tetraponera nitida* (F. Smith, 1860b)	Md, Ng, Pl	
*Tetraponera pilosa* (F. Smith, 1858)	Pl	
*Vollenhovia ambitiosa* Menozzi, 1925	Lz, Md	
*Vollenhovia banksi* Forel, 1910	Ng	
*Vollenhovia cristata* (Stitz, 1938)	Pl	
*Vollenhovia luctuosa* (Stitz, 1938)	Pl	
*Vollenhovia oblonga dispar* Forel, 1910	Lz	
*Vollenhovia soleaferrea* Donisthorpe, 1942	Lz	
*Vombisidris philippina* Zettel & Sorger, 2010a	Cb	

Key to Notes: A/G = Alpert and General Collection BPBM = Bernice P. Bishop Museum Collection HZW = Herbert Zettel (Vienna) Collection MCZ = Museum of Comparative Zoology Collection UPLB = University of the Philippines at Los Baños Museum of Natural History Collection SI = Smithsonian Institution Museum Collection Key to Island Abbreviations: Ba = Bantayan Bh = Bohol Bi = Biliran Bs = Basilan By = Bayagnan Cb = Cebu Cm = Camiguin Ct= Catanduanes Jo = Jolo Ly = Leyte Lz = Luzon Md = Mindanao Mq = Marinduque Mr = Mindoro Ms = Masbate Ng = Negros Pc = Pacijan Pl = Palawan Pn = Panay Po = Polillo Rm = Romblon Rr = Rapu-rapu SC = Sta. Cruz Si = Sibutu Sm = Samar Sy = Sibuyan Tb = Tablas Tw = Tawi-tawi W = Widespread

## Appendix

### Checklist of valid ant species known to occur in the Philippines, arranged alphabetically by genus.

There are 474 ant species and subspecies known from the Philippines and at least an additional 100 undescribed or unidentified species represented by museum specimens and recent collections. The total size of the ant fauna is no doubt much larger, almost certainly more than 1000 species are present. This tentative list contains species and subspecies names regarded as valid in Bolton (2011) and later publications. It is meant to serve as a rough guide to our current knowledge of the Philippine ant fauna. Keys to the notes and island codes are at the end of the list.
